# Evaluation of the FI-Lab, a laboratory-based, automated frailty index for acute care: A multicohort study

**DOI:** 10.1371/journal.pmed.1005004

**Published:** 2026-09-01

**Authors:** Hugh Logan Ellis, Peter Hanlon, Liam Dunnell, Martin Whyte, Daniel H. J. Davis, Josephine Bates, Adeel Jafri, Diana Shamsutdinova, James T. Teo, Zina Ibrahim, Kenneth Rockwood

**Affiliations:** 1 Department of Biostatistics and Health Informatics, King’s College London, London, United Kingdom; 2 Division of Geriatric Medicine, Department of Medicine, Dalhousie University, Halifax, Nova Scotia, Canada; 3 Department of Endocrinology, St George's Healthcare NHS Trust, London, United Kingdom; 4 School of Health and Wellbeing, University of Glasgow, Glasgow, United Kingdom; 5 Department of Clinical Gerontology, King’s College Hospital, London, United Kingdom; 6 Department of Diabetes, Kings College Hospital, London, United Kingdom; 7 School of Biosciences, University of Surrey, Guildford, United Kingdom; 8 Institute of Health Informatics, University College London, London, United Kingdom; 9 Faculty of Life Sciences & Medicine, King’s College London, London, United Kingdom; 10 Oncology Department Western General Hospital Edinburgh, United Kingdom; 11 Department of Neurology, King’s College Hospital, London, United Kingdom; 12 Cleveland Clinic Hospital London, London, United Kingdom; 13 Centre for Health Care of the Elderly, Nova Scotia Health, Halifax, Nova Scotia, Canada; Waymark, UNITED STATES OF AMERICA

## Abstract

**Background:**

Laboratory-based frailty indices (FI-Lab) have shown promise in geriatric medicine research. We aimed, in diverse samples, to determine the optimal construction of an FI-Lab for acute care and evaluate its validity as a measure of latent health status across the adult life span.

**Methods and findings:**

Our retrospective multicohort study used emergency department encounters in Boston, USA (MIMIC-IV-ED; 2011–2019) and London, UK (King’s College Hospital (KCH); 2017–2020); and a community-based cohort in the UK (UK Biobank; 2006–2010). We evaluated FI-Lab configurations by varying test selection strategies, number of tests, and minimum test thresholds. Sensitivity analyses examined performance across age, ethnicities, sexes, and model types to identify potential blind spots. The primary outcome was 1-year all-cause mortality.

We analysed 227,736 visits (113,032 patients) from MIMIC-IV-ED; 152,305 visits (96,843 patients) from KCH; and 492,703 UK Biobank. An FI-Lab calculated from 25 commonly ordered tests (minimum 15 required) demonstrated hazard ratios for 1-year mortality approaching those of chronological age and exceeding the National Early Warning Score 2 (NEWS2). In combined models, hazard ratios for FI-Lab per standard deviation increase were 2.04 (95 %CI [1.99, 2.09]; *p* < 0.001) in MIMIC-IV-ED, 2.55 (95 %CI [2.42, 2.69]; *p* < 0.001) in KCH, and 1.87 (95% CI [1.80, 1.94]; *p* < 0.001) in UK Biobank. Performance was consistent across subject groupings. Discrimination plateaued at 20–40 tests. This was a retrospective study; future work exploring the impact on everyday use requires prospective evaluation, potentially in a randomised controlled trial.

**Conclusions:**

The FI-Lab provides an automated, scalable measure of patient vulnerability that is robust across healthcare settings and populations. A core set of 20–40 commonly ordered tests is sufficient for signal capture. It offers a pragmatic complement to clinical judgement without requiring manual data entry or additional tests.

## Author summary

**Why was this study done?:**

- Healthcare providers frequently ponder the question, “How sick is this person?”. Even so, there is no simple way to summarise overall illness severity using routinely collected clinical data.
- The laboratory frailty index (FI-Lab for short)—based on the proportion of abnormal laboratory test results—is an established measure of frailty in older adults.
- We investigated whether the FI-Lab could be used as a general measure of illness severity across all adult ages and in different healthcare settings.

**What did the researchers do and find?:**

- We used already-collected healthcare data for almost 900,000 adults from three datasets: emergency department visits in Boston (MIMIC-IV-ED) and London (King’s College Hospital), and a UK community cohort (UK Biobank).
- We tested multiple FI-Lab models by varying the laboratory tests included and compared their performance with age and standard health deterioration scores.
- A simple FI-Lab built from approximately 25 commonly-ordered blood tests is as strongly associated as age with risk of dying, hospital admission, and readmission, while standard deterioration risk scores perform much worse.
- The FI-Lab works regardless of age, sex, ethnicity, or tested healthcare system.

**What do these findings mean?:**

- FI-Lab provides a fast, automatic, and transparent measure of illness severity, without needing extra testing or bedside assessment.
- The same approach could be extended beyond blood tests to vital signs and other routinely collected data that can be classified as normal or abnormal.
- An approach originally developed for older people can provide a broad indicator of health across the adult life span and could aid clinical assessment.
- Even though this retrospective study using data from two high-income countries shows the FI-Lab reliably tracks poor outcomes, more research is needed to determine whether real-time access to the FI-Lab improves clinical decision-making or patient care.

## Abstract 3

In this multicohort study, Hugh Logan Ellis and colleagues optimize the laboratory-based frailty index for use as a general measure of illness severity across all adult ages and different healthcare settings.

## Introduction

As populations age and medical care successfully extends survival with chronic illness, chronological age has become an increasingly poor proxy for patient vulnerability [1,2]. Two 85-year-olds presenting to an emergency department might share a birth year but possess vastly different physiological reserves. While medicine has adopted the language of frailty to describe this heterogeneity in older adults, clinical assessment tools have not evolved to match the data now available. Electronic health records (EHRs) capture a detailed digital profile for every patient, laboratory panels, imaging sequences, telemetry streams, and medication histories, yet decision-making relies on simpler metrics. The UK National Early Warning Score 2 (NEWS2) [3], for all its merits, uses six vital signs and underestimates risk for older and frailer patients [4,5]. With ever more complex patients, the gap widens between what we measure and what we score.

Why haven’t we closed this gap? The era of big data and machine learning (ML) has produced a proliferation of risk prediction models, yet clinicians worldwide continue to use decades-old risk scores. Though promising, learning from historical data is limited by the absence of counterfactuals: we cannot observe what might have happened with different treatment decisions or under alternative standards of care [6]. Models can only recognise patterns present in their training data. This perpetuates historical inequities and variations in care [7,8]. Beyond these fundamental constraints, practical barriers compound the problem: ML models require mature technological infrastructure and site-specific calibration [9]. Traditional scores like the Acute Physiology and Chronic Health Evaluation (APACHE) score require recalibration [10,11] and complete data collection [12], undermining widespread use. As a result, simple measures like NEWS2 remain the standard, despite their limitations.

Building on the well-established deficit accumulation model of frailty [13], the laboratory-based frailty index (FI-Lab) [14] offers an approach that differs from prediction models and is rooted in the mathematical principles describing complex systems at risk of collapse [15,16]. Rather than predicting specific outcomes, frailty indices such as FI-Lab quantify physiological reserve with a simple calculation: count abnormal results and divide by total tests performed, providing passive, data-driven measures of latent health status. Evidence in older adults shows the FI-Lab can be adapted to effectively measure both acute illness severity and chronic frailty [17], with strong associations to such key outcomes as mortality and readmissions [18,19].

Although FI-Labs have predominantly been used in frailty in older adults, the theory of deficit-accumulation is agnostic to age or setting [17–19]. The proportion of abnormal biological parameters captured in the FI-Lab is an index of underlying dysregulation, making it a latent measure of overall health. In acute settings, this will be a measure of both chronic health status (a combination of overlapping concepts such as frailty, performance status, physiological reserve and baseline) and acute illness severity. If the FI-Lab can capture this underlying construct, its prognostic signal—and with that, its ability to identify multiply- determined risk that increases with age—should generalise across the adult life span, healthcare settings, and outcomes. To realise the potential of the FI-Lab, we aimed to:

1. **Identify the optimal construction method** by determining which laboratory test selection strategy performs best across datasets and the optimal number of tests that balances discriminative ability with patient coverage;
2. **Compare performance** to established metrics including NEWS2 and patient age; and
3. **Evaluate generalisability** by assessing whether the FI-Lab works effectively across diverse populations, ages, sexes, ethnicities, socioeconomic groups, countries, and clinical contexts.

## Methods

### Study design and data sources

We conducted a retrospective multi-cohort study using routinely collected EHR data from two hospital systems (Beth Israel Deaconess Medical Center [MIMIC-IV-ED; [20]], Boston, 2011–2019; King’s College Hospital [KCH], London, 2017–2020) and one prospective research cohort (UK Biobank, 2006–2010). We selected emergency department encounters in the EHR data to capture unplanned hospital presentations with baseline laboratory testing before intervention.

### Study population

We included adults (≥18 years) from MIMIC-IV-ED and from KCH EDs where we could link same-day laboratory data by patient identifier and timestamp. UK Biobank participants (aged 37–73) were recruited from 22 assessment centres and provided blood samples.

### Exclusions

Patients without linkable same-day laboratory data (e.g., minor injuries without blood tests; outside referrals with tests not repeated) were excluded. For mortality analyses, only first visits were analysed. At KCH, additional exclusions were: nonbinary gender records, deaths recorded before ED arrival time, and impossible (negative) or implausible (>1,000 days) hospital stays and unparseable datetime records.

### Primary and secondary outcomes

The primary outcome was all-cause mortality within 1 year of ED presentation or UK Biobank baseline assessment. Secondary outcomes were in-hospital mortality, hospital length of stay, 30-day hospital readmission, 1-year hospital (re)admission, and hospital admission from the ED and in the UK Biobank mortality up to 10 years.

### Laboratory-based frailty index construction

For each patient, an FI-Lab score was calculated following the deficit-accumulation technique for laboratory data established by Howlett and colleagues [14] as the proportion of abnormal test results to the number of tests performed from a pre-specified list. We used the first test result within 24 hours for each parameter. Tests coded abnormal reflected site-specific laboratory reference ranges. As the FI-Lab accommodates varying test numbers by adjusting the denominator to each patient’s performed tests, no imputation is required.

To identify an optimal construction method, we systematically evaluated FI-Lab configurations by varying three parameters:

1. **Test Selection Strategy:** We compared lists created from the most ‘Common’ tests ordered versus lists of the most prognostically ‘Important’ tests for 1-year mortality (determined via a random forest model) and with lists created randomly.
2. **Number of Tests:** We varied the size of the test list (from 10 to 70).
3. **Feature Floor:** We varied the minimum number of tests required for a patient’s score to be calculated, from no imposed minimum to 20 tests.

The Supplementary Appendix details construction and validation methods, the full list of tests considered (Tables C–F in S1 Appendix), and the NEWS2 calculation.

### Statistical analysis

We used Cox proportional hazards models to evaluate associations between FI-Lab scores and outcomes. To compare performance, we used nested models: demographics (age/sex) alone, with NEWS2, with FI-Lab, and with both scores. Continuous variables were scaled before analysis. Hazard ratios are reported per standard deviation increase with 95% confidence intervals (CI). One standard deviation of the FI-Lab corresponds to ~0.10 in the ED cohorts and 0.06 in UK Biobank. One standard deviation of age corresponds to ~20 years in the ED cohorts and 8 years in UK Biobank. We use C-statistics to assess discrimination to validate FI-Lab as a measure, not to develop a prediction model. We used AIC to assess model fit.

For mortality and admission outcomes, time-zero was ED presentation. For readmission analyses, time-zero was hospital discharge with censoring at death or study end (30 days or 1 year). Given the exploratory nature of configuration analyses, we did not adjust for multiple comparisons.

To quantify FI-Lab reliability, recognising that similar patients can have different test combinations ordered, we assessed: (1) sampling variation, by calculating 20 random test combinations per patient at various feature set sizes and (2) measurement uncertainty, by adding realistic noise to laboratory values near cut-points.

To further evaluate generalisability, we selected 25 commonly ordered tests with a feature floor of 15 for sensitivity analyses with 1 year mortality as our outcome of interest. We examined incorporation of vital signs as binary features, stratification by 10-year age bands, stratification by sex (gender was used as a proxy in the KCH dataset as sex was not routinely recorded), stratification by socioeconomic status and ethnicity and use of a competing risk model for readmission. Finally, to assess the utility of FI-Lab alongside other measures of frailty, we examined associations between FI-Lab and mortality (1-year and 10-year) across baseline strata of two existing UK Biobank frailty measures: a cumulative deficit frailty index based on 49 self-reported diagnoses and symptoms [21] and an adaptation of the Fried Frailty Phenotype (comprising weight loss, low physical activity, exhaustion, slow-walking speed (all self-reported) and measured low grip strength with 3 or more items indicating frailty, and 1–2 items a pre-frail state [22]). Analyses used R (v4.5.0/v4.2.1).

Our reporting follows the Strengthening the Reporting of Observational Studies in Epidemiology (STROBE) guidelines [23] and the Reporting of studies Conducted using Observational Routinely-collected health Data (RECORD) extension [24]. A completed STROBE-RECORD checklist is provided in the supplementary appendix (S1 Checklist).

The research questions and conceptual framework were presented at patient involvement sessions organised through King’s College Hospital.

### Ethics approval

We utilised deidentified data from three research databases. The MIMIC-IV-ED database [20] is publicly available following completion of required training. UK Biobank analysis was conducted under application 14,151. Use of KCH data was approved by the King’s Electronic Patient Record Interface Committee (approval ID 20230411B). UK Biobank has ethical approval from the North West Multi-centre Research Ethics Committee. Formal consent was not required for this secondary use of deidentified data.

### AI statement

Claude Opus 4.0 (Anthropic) was used to refine code as necessary, primarily to improve efficiency, comprehensibility, and to streamline ggplot syntax used for visualisation. The authors manually tested the code and relevant outputs post-iteratively to ensure accuracy.

## Results

### Study population

From the US-based MIMIC-IV-ED database (2011–2019), we identified 425,011 emergency department visits; after applying eligibility criteria, we analysed 227,736 visits from 113,032 unique patients. From the UK-based King’s College Hospital database, we identified 449,060 records (2017–2020); after exclusions, 152,305 visits from 96,843 unique patients were analysed. From the UK Biobank cohort (2006–2010 baseline recruitment of 502,533 participants), 492,703 with available blood test data were included. (STROBE flow diagram Fig A in S1 Appendix).

The MIMIC-IV-ED cohort was ~8 years older than King’s, with proportionately more patients aged ≥60 years (Table 1). 1-year mortality and hospital admission rates were higher in MIMIC-IV-ED, though in-hospital mortality was lower.

**Table 1:** Baseline characteristics of patients in the MIMIC-IV-ED and King’s College Hospital Emergency Department Cohorts.

Characteristic	MIMIC-IV-ED cohort	KCH cohort	UK Biobank
**Cohort size**			
Total ED Visits, *n*	227,736	152,305	
Unique patients, *n*	113,032	96,832	492,703
**Patient demographics**			
Age, years, Mean (SD)	52.07 (20.94)	43.8 (18.8)	56.5 (8.1)
Age group, *n* (%)			
18–39 years	37,312 (33.0)	37,734 (39.0)	
40–59 years	31,609 (28.0)	31,195 (32.2)	285,036 (57.9)
60–79 years	30,881 (27.3)	19,215 (19.8)	217,497 (43.3)
≥ 80 years	13,230 (11.7)	8,703 (9.0)	
Female, *n* (%)	62,087 (54.9)	52,254 (54.0)	273,401 (54.4)
Ethnic group, *n* (%)			
White	71,513 (63.3)	43,867 (45.3)	464,105 (94.2)
Black or Black British/ African American	17,851 (15.8)	27,891 (28.8)	7,801 (1.6)
Asian or Asian British	5,654 (5.0)	3,907 (4.0)	9,620 (2.0)
Mixed		2,747 (2.8)	2,885 (0.6)
Other ethnic group	7,309 (6.5)	8,176 (8.4)	4,430 (0.9)
Prefer not to answer		9,694 (10.0)	2,335 (0.5)
Chinese		557 (0.6)	1,527 (0.3)
Unknown	3,490 (3.1)		
Latino/Hispanic	7,215 (6.4)		
NA		8 (0.0)	
Insurance category			
Medicare	27,090 (24.0)		
Medicaid	10,885 (9.6)		
Private	22,349 (19.8)		
Other/Unknown	3,824 (3.4)		
NA	48,884 (43.2)		
Index of multiple deprivation decile, Mean, (SD)		4.00 (1.95)	
Townsend score, mean (SD)			−1.29 (3.10)
**Total admissions, *n* (%)**			
1 Visit	75,166 (74.8)	71,512 (73.8)	
2 Visits	17,779 (17.7)	14,236 (14.7)	
3+ Visits	7,538 (7.5)	11,099 (11.5)	
**Presenting acuity and outcomes**			
NEWS2 score, mean (SD)	0.94 (1.32)	1.21 (1.58)	
Hospital admission, *n* (%)	51,115 (45.2)	30,221 (31.2)	
In-hospital mortality, *n* (%)	1,676 (2.6)	1,182 (3.9)	
30-day readmission, *n* (%)	8,783 (13.6)	7,721 (8.0)	
1-year all-cause mortality, *n* (%)	8,202 (7.3)	3,222 (3.3)	965 (0.2)
**Laboratory testing patterns**			
Patients with ≥10 tests, *n* (%)	107,991 (95.5)	93,133 (96.2)	490,255 (99.5)
Patients with ≥20 tests, *n* (%)	106,991 (94.6)	89,373 (92.3)	489,072 (99.3)
Patients with ≥30 tests, *n* (%)	94,996 (84.0)	66,359 (68.5)	457,646 (92.9)

Data are presented as mean (SD) for continuous variables and *n* (%) for categorical variables. KCH denotes King’s College Hospital, NEWS2 National Early Warning Score 2, and SD standard deviation.

**Fig 1:**
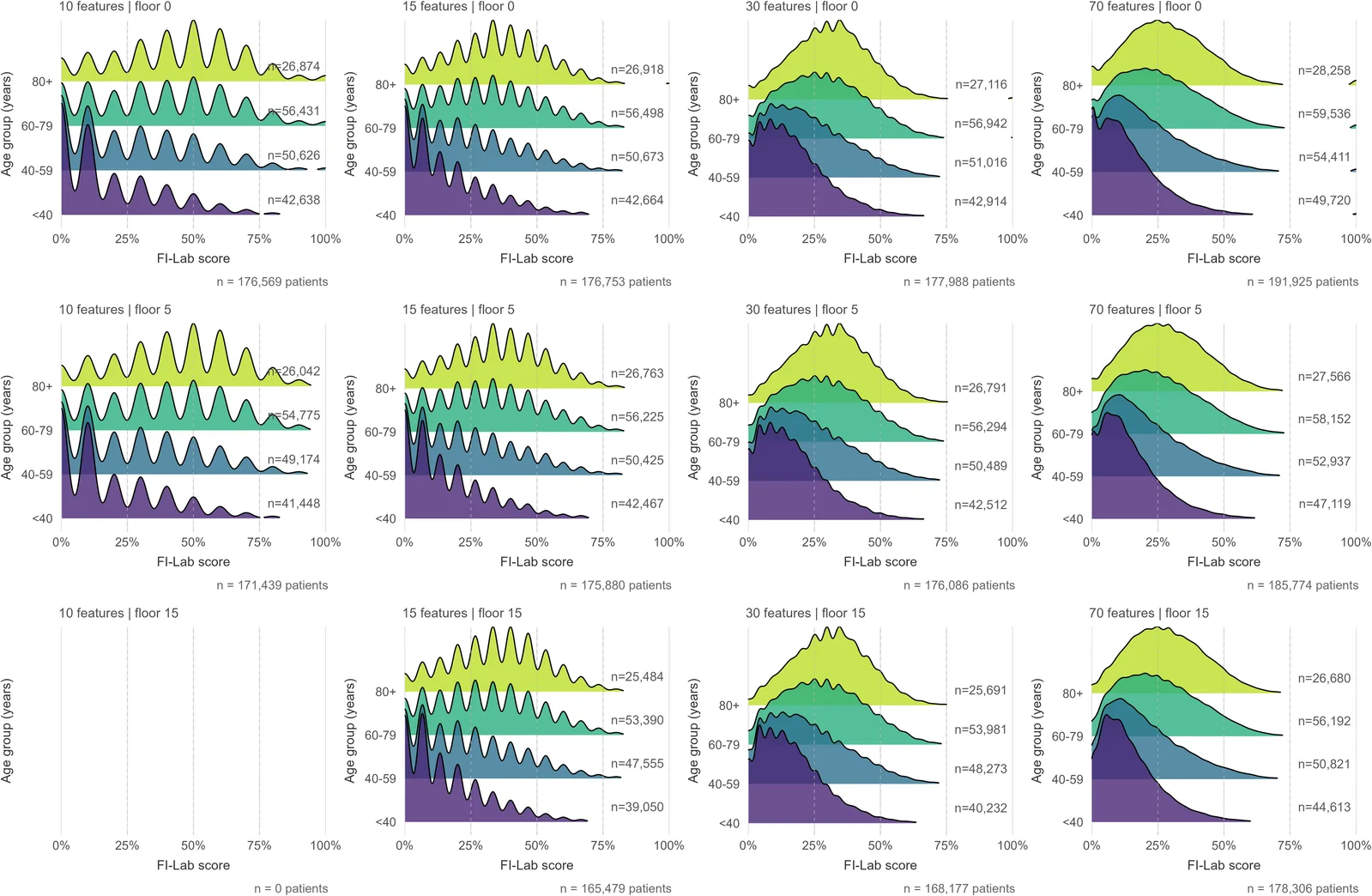
FI-Lab score distributions across age groups, feature number, and feature floor in MIMIC without observations (vital signs). Each panel shows the distribution of FI-Lab scores (proportion of abnormal laboratory tests) by age group, varying by the number of included features (columns: 10, 15, 30, and 70) and the minimum feature floor (rows: 0, 5, and 15). The feature floor defines the minimum number of available laboratory tests required for an FI-Lab score to be calculated for a given individual. Age groups are displayed on the y-axis from <40 to 80+ years. The total number of patients per panel is shown at the bottom. As both the number of features and the minimum feature floor increase, the score distributions become more tightly clustered around mid-range values, and the proportion of patients with very high FI-Lab scores (>70%) decreases substantially. This reflects expected statistical properties: as more features are required, extreme scores become less likely, and the index becomes less sensitive to single abnormal results. Panels with insufficient data to meet the minimum feature floor display no patients (e.g., 10 features with a floor of 15).

The distribution of FI-Lab scores varied by age and by the number of tests (features) used to calculate the score (Fig 1).

### Configuration analysis

Measurement stability improved progressively with increasing test numbers. Coefficients of variation decreased from 1.65 (15 tests) to 0.34 (70 tests) in MIMIC-IV-ED, with similar patterns in KCH (Figs B, C in S1 Appendix). With measurement noise added to simulate assay variability, coefficients of variation fell from 0.30 (10 tests) to 0.16 (≥40 tests) (Fig D in S1 Appendix).

**Fig 2:**
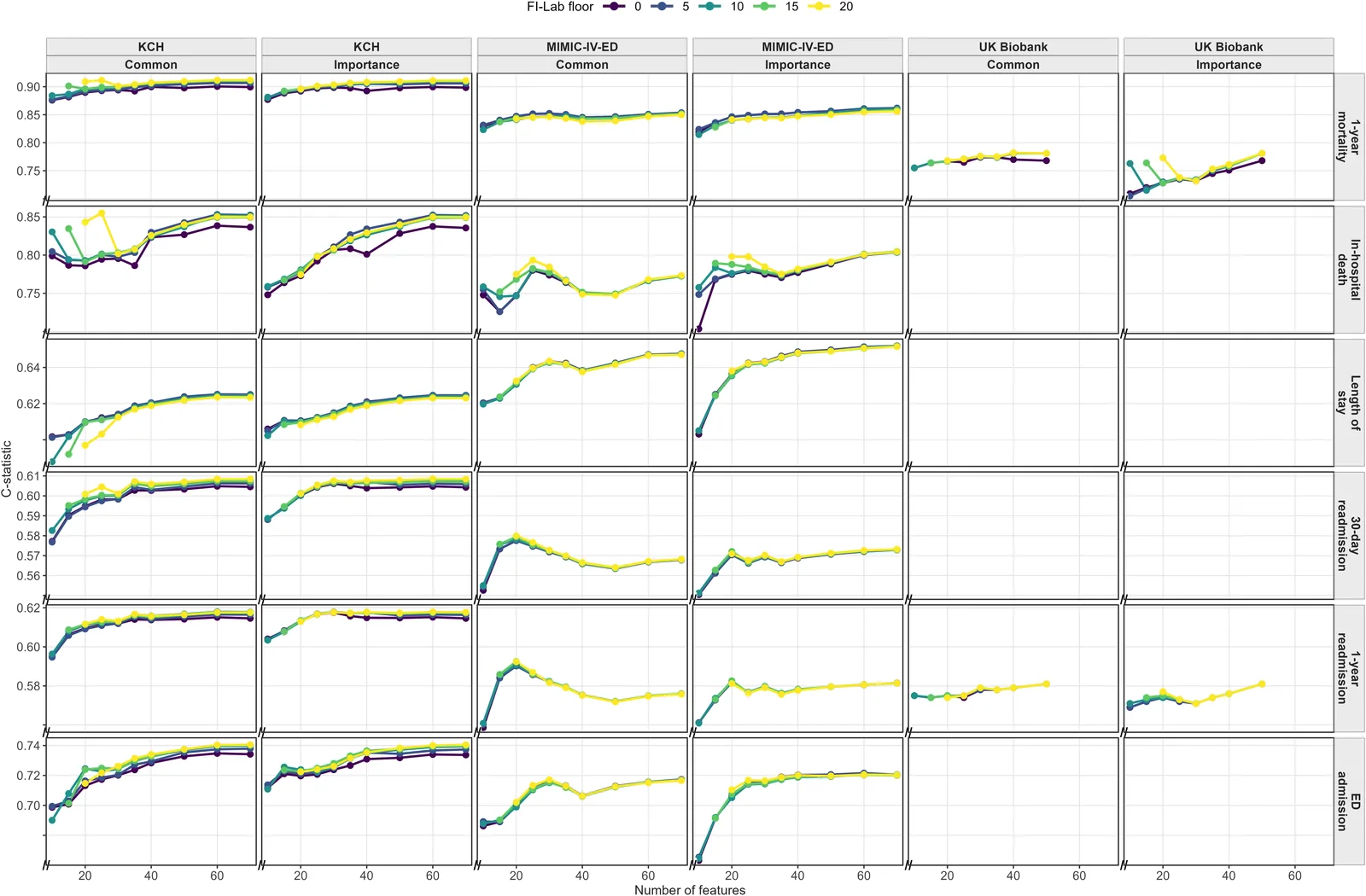
Discrimination (C-statistic) of FI-Lab models across outcomes, datasets, and selection strategies. Rows show outcomes; columns show dataset × strategy (Common, Importance). Lines are FI-Lab models coloured by the minimum-tests floor (0–20); points indicate the number of features included. The y-axis shows Harrell’s C-statistic (scales are fixed within rows but free between rows). Blank panels in UK Biobank indicate outcomes not available in that dataset. UK Biobank lacks readmission; 1-year hospital admission is used as the comparator and plotted under “1-Year Readmission.” Across datasets, the C-statistic typically rises with 10–30 features and then plateaus, with only small differences of modest influence between Common and Importance strategies.

Model discrimination showed a different pattern: performance improved with initial test inclusion but plateaued beyond 20–30 tests, with minimal further gains or occasional decrements (Figs 2 and E–I in S1 Appendix).

For 1-year mortality in KCH, C-statistics were 0.895 (20 tests), 0.901 (25 tests), and 0.902 (70 tests). In MIMIC, some secondary outcomes showed performance decrements with additional tests: 30-day readmission C-statistics decreased from 0.585 (20 tests) to 0.577 (70 tests). When sample sizes remained constant, varying the number of tests made little difference to model fit as measured by AIC (Fig J in S1 Appendix).

Tests selected by frequency of ordering achieved C-statistics within 0.002 of tests selected by importance for the 25-test configuration (MIMIC: 0.845 versus 0.843; KCH: 0.901 versus 0.901). Both strategies outperformed random selection (mean C-statistic 0.738 in MIMIC, 0.751 in KCH). A feature floor of 15 maintained >90% patient coverage while reducing score variability by 60% compared to no floor requirement (Fig K in S1 Appendix).

In UK Biobank, discrimination did not plateau with either selection strategy: for 1-year mortality, C-statistics rose from 0.776 to 0.781 for Common and from 0.732 to 0.781 for Importance when increasing from 30 to 50 features with a floor of 20.

Table 2 summarises the effects of changes to configuration parameters on performance.

**Table 2:** Configuration trade-offs in FI-Lab development.

Configuration choice	Effect on variance	Effect on patient coverage	Effect on performance
**Increasing feature floor**	Substantially reduces	Moderately reduces	Modest; reflects cohort selection (see Discussion with age - should generalise across the adult life span, healthcare settings, and outcomes)
**Adding more features**	Reduces	Moderately increases	Improves then declines or plateaus
**Including vital signs**	Minimal effect	Increases coverage	Better for short-term outcomes
**Importance vs. common selection**	Not applicable	Dramatically lower for importance in some datasets (dependent on how importance is defined)	Differences are very small

### Performance compared to established measures

**Fig 3:**
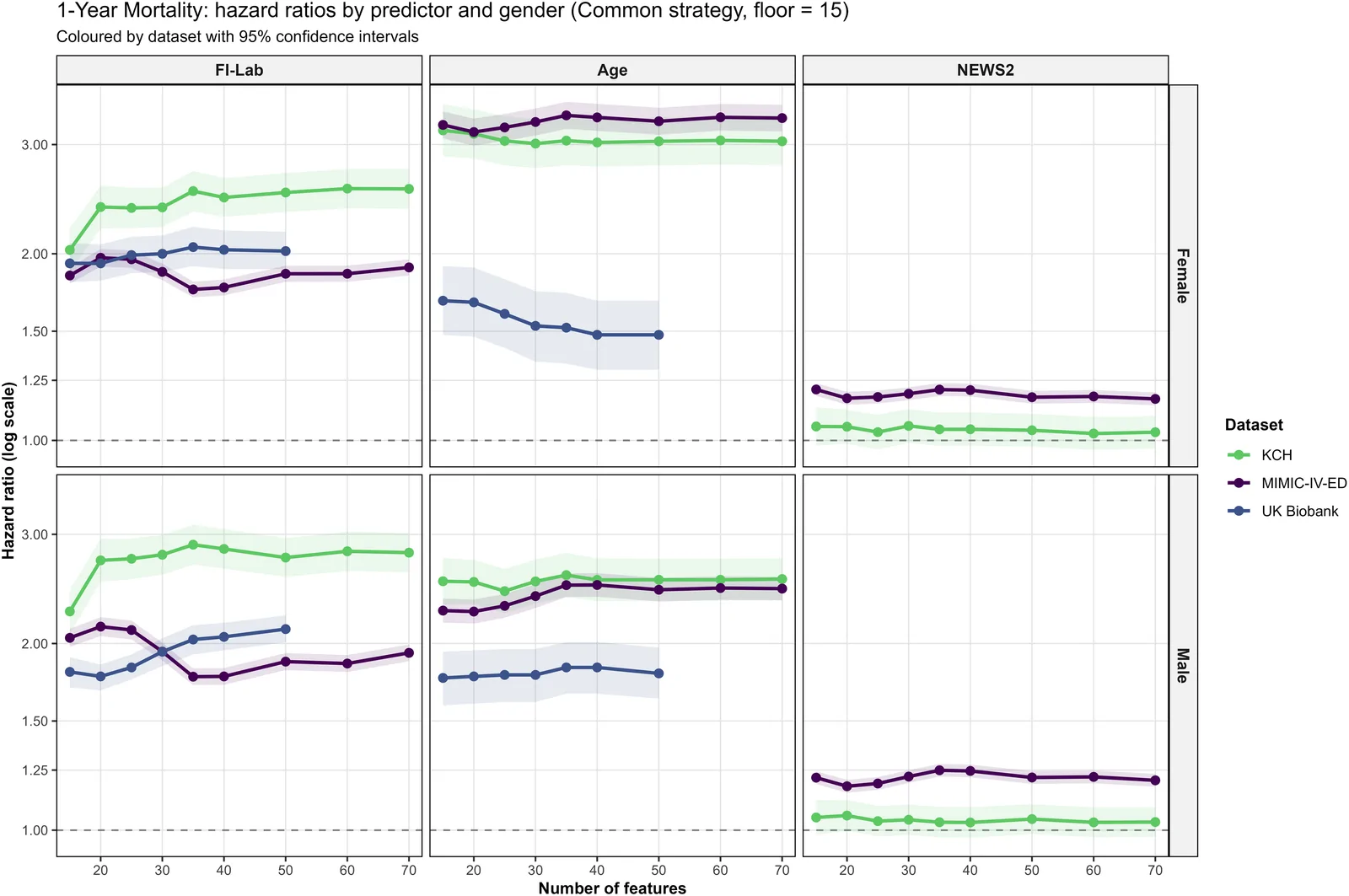
Hazard ratios for 1-year mortality by variable type and gender across three datasets. Columns show variable types (FI-Lab, Age, and NEWS2); rows show gender (Female, Male). Lines represent hazard ratios by dataset (KCH in green, MIMIC-IV-ED in purple, UK Biobank in blue) across numbers of laboratory features (used to compute the FI-Lab) with the Common feature-selection strategy (minimum-tests floor = 15). All variables are shown with solid lines, points, and shaded 95% confidence intervals. NEWS2 was not available in UK Biobank. The y-axis is log_2_-scaled; the dashed horizontal line marks a hazard ratio of 1.

We chose 25 common tests with a feature floor of 15 as our reference configuration. In combined models, the FI-Lab was associated with hazard ratios for 1-year mortality of 2.04 (95% CI [1.99, 2.09]; *p* < 0.001) in MIMIC-IV-ED, 2.55 (95% CI [2.42, 2.69]; *p* < 0.001) in KCH, and 1.87 (95% CI [1.80, 1.94]; *p* < 0.001) in UK Biobank, with C-statistics of 0.847, 0.911, and 0.771, respectively. In the same models, the hazard ratios for age were 2.69 (95% CI [2.60, 2.78]; *p* < 0.001) in MIMIC-IV-ED, 2.70 (95% CI [2.54, 2.87]; *p* < 0.001) in KCH, and 1.72 (95% CI [1.59, 1.85]; *p* < 0.001) in UK Biobank. The hazard ratios for NEWS2 were 1.08 (95% CI [1.06, 1.10]; *p* < 0.001) in MIMIC-IV-ED and 1.03 (95% CI [0.99, 1.08]; *p* = 0.13) in KCH; NEWS2 was unavailable in UK Biobank. Figure 3 demonstrates FI-Lab’s (sex-stratified) associations approach or exceed age and consistently exceed NEWS2 across all datasets.

### Secondary outcomes

For 30-day readmission FI-Lab was associated with hazard ratios of 1.32 (95% CI [1.30, 1.33]) in MIMIC and 1.35 (95% CI [1.33, 1.36]) in KCH and 1.36 (95% CI [1.35, 1.37]) in MIMIC and 1.33 (95% CI [1.32, 1.34]) in KCH for 1-year readmission. Age showed the strongest association with mortality, FI-Lab showed the strongest association for readmission, ED admission, and length of stay. NEWS2 consistently showed the weakest association across outcomes (Figs L–P in S1 Appendix).

### Sensitivity analyses

FI-Lab performance was consistent across sexes, with greater hazard ratios in males in ED cohorts (MIMIC: HR 2.10 versus 1.96; KCH: HR 2.74 versus 2.37) and in females in UK Biobank (HR 1.99 versus 1.83), with overlapping CI in all comparisons.

FI-Lab was associated with mortality across all age bands. Hazard ratios peaked at ages 30–60 years (range 2.11–3.16) and declined to 1.77–2.11 in those aged ≥80 years, but remained significant across all groups (*p* < 0.001; detailed HRs and 95% CIs in Table A in S1 Appendix).

FI-Lab was significantly associated with mortality across all ethnic groups except Chinese in UK Biobank (fewest events). Hazard ratios were broadly similar, though ethnicity interactions were statistically significant in all cohorts (*p* < 0.001, Table B and Fig Q in S1 Appendix).

Adding vital signs to the FI-Lab improved discrimination for in-hospital mortality (C-statistics increased from 0.750 to 0.782 in MIMIC, *p* < 0.001 (Fig B in S1 Appendix); and from 0.797 to 0.801 in KCH, *p* < 0.001) (Fig C in S1 Appendix) but not for 1-year mortality (C-Statistics 0.845–0.845 in MIMIC; 0.901–0.899 in KCH).

Using Fine–Gray subdistribution hazards (SHR) attenuated-hazard model for readmission on a subset of patients (MIMIC-IV-ED; 25 Common tests; feature floor 15; 50,000-patient subset), accounting for death as a competing risk, attenuated ratios by ~2%–3% relative to cause-specific estimates (30-day readmission SHR 1.29, 95% CI [1.27, 1.32]; *p* < 0.001 versus cause-specific HR 1.32; 1-year readmission SHR 1.32, 95% CI [1.31, 1.34]; *p* < 0.001 versus cause-specific HR 1.36).

When stratifying UK Biobank data by baseline levels of other frailty measures (a deficit accumulation FI based on diagnoses/symptoms, and an adapted Frailty Phenotype), a higher FI-Lab was associated with increased risk of 1- and 10-year all-cause mortality across each strata of baseline frailty status by both frailty measures (Tables G, H in S1 Appendix). When including the FI based on diagnoses/symptoms and the FI-Lab in the same model, both were associated with higher 10-year mortality with broadly comparable hazard ratios (Table I in S1 Appendix).

## Discussion

In this large, multicohort study, we found that a laboratory-based frailty index constructed from routine blood tests provided prognostic information with signal strength approaching that of chronological age and superior to NEWS2 (representative of the current paradigm for acute severity assessment). The FI-Lab demonstrated robust associations with outcomes ranging from emergency department admission to mortality. Critically, this signal remained consistent across the full adult age spectrum, all ethnic groups, and different healthcare systems, and was detectable up to 10 years after measurement. This consistency suggests that the FI-Lab captures something fundamental about patient vulnerability, independently of where, when, or in whom it is measured. An optimal FI-Lab balances performance with measurement stability and patient coverage, typically achieved with a core set of 20–40 commonly ordered laboratory tests. We are persuaded that FI-Lab captures a latent property of health, combining both elements of chronic health status and acute illness severity. In this sense, the deficit accumulation model could serve as a general-purpose instrument for measuring latent health status, extending the application of FI-Labs from geriatrics into routine clinical care.

FI-Lab performance has an early peak with 20–40 features and for some outcomes does not improve indefinitely. This provides a crucial, evidence-based answer to the question of how to best construct an FI-Lab. It resolves a key uncertainty in the literature, where FI-Lab configurations vary widely [18]. The performance plateau suggests that a core set of common tests captures sufficient physiological derangement for prognostication. The decline seen when including rare tests might reflect selection bias inherent in clinical care data [25,26]. That these tests are ordered when pathology is already suspected, gives them a high pre-test probability of being abnormal (Fig R in S1 Appendix).

The FI-Lab showed consistent performance across all adult age groups. While frailty is traditionally associated with older adults, our data show that the physiological dysregulation captured by the FI-Lab is strongly associated with adverse outcomes in younger adults as well. This aligns with other work suggesting the accumulation of deficits reflects underlying vulnerability regardless of age [22,27–33]. As shown in Fig 1, with a feature floor of 15 the score distribution broadens with increasing age while maintaining a submaximal limit well short of 1.0—the same characteristic behaviour described for frailty indices of varying composition. In this respect, the FI-Lab is a frailty index in the formal, properties-based sense. Furthermore, as in Tables B and E in S1 Appendix, we see that, in both the UK-Biobank’s two measures of frailty (index [21] and phenotype [22]), the FI-Lab is associated across grades of fitness/robustness/prefrailty/frailty with an increasing dose-response in both the 1-year and 10-year hazard ratios. In short, the FI-Lab correlates with and adds to the information provided by these two measurement approaches. We recommend a pragmatic approach to FI-Lab construction for clinical implementation: use the most commonly ordered tests at a given institution with a minimum of 15 tests required for score calculation. This strategy optimises the trade-off between associative strength, score stability, and patient coverage. Importance-based selection offered no meaningful benefit and reduced coverage. Include any routinely performed investigation with binary normal/abnormal classification that shows variation, but exclude clinician-determined variables such as diagnoses or medications that can introduce subjective bias [25,34].

The inherent variability of the FI-Lab, stemming from both binomial sampling effects and laboratory measurement noise, underscores that it should be interpreted as an estimate of general vulnerability, not a precise measurement. We recognise that, employing values from ED visits, in many cases the FI-Lab might represent the severity of acute illness rather than frailty. Note however that the UK Biobank FI-Lab remained associated with mortality at hazard ratios per SD of 1.87 (95% CI [1.80, 1.94]) and at time horizons of up to 10 years. Were the ED cohorts purely signalling acute illness severity, it should be absent in UK Biobank; it is not. It is likely a combination of both. Finally, we position the FI-Lab not to replace clinical judgment, but as a data-driven frailty (or fitness) measure to help clinicians rapidly contextualise a patient’s underlying health in light of their chronological age. In practice, this could identify the 50-year-old whose EHR data suggest the vulnerability of someone decades older, or the 80-year-old with remarkably preserved physiology. Though age remains relevant, FI-Lab captures vulnerability not reflected by age alone. It does so without requiring any additional testing, as the items that compose it can be pulled from test results as they become available.

The retrospective design establishes associations but not causation. Data from two high-income countries with mature EHR infrastructure might not generalise to other healthcare settings or resource-constrained environments. Despite adjusting for age and NEWS2, unmeasured confounders remain. We used standard Cox models rather than competing risk models potentially underestimating readmission incidence, though our sensitivity analysis suggests model choice only marginally impacts effect size. Computational cost prevents us from running these analyses across all cohorts and configurations, so cause-specific hazards are reported as primary. We tested multiple configurations without adjustment for multiple comparisons and did not test proportional hazard assumptions. Our linkage methodology likely underestimated laboratory coverage, particularly for pre-emergency department tests. We recognise that using the same cohort for feature selection and evaluation is a potential source of overfitting for the ‘Importance’ strategy in MIMIC-IV-ED. Most importantly, whether FI-Lab measurement improves clinical outcomes requires prospective evaluation, preferably extended to a range of countries. Laboratory reference ranges were standard adult ranges and were not age-stratified. The smaller effect size observed in UK Biobank could plausibly reflect, in part, a higher proportion of deaths from causes that are not physiologically predictable in a community cohort compared with patients presenting to hospital. Additional methodological considerations are discussed in the Supplementary S1 Appendix.

The laboratory-based frailty index is a simple, powerful, transparent, and scalable tool for quantifying patient vulnerability. An optimal FI-Lab can be constructed using a core set of the most commonly available laboratory results, with a minimum of 15 tests suggested for stability. As a passive, data-driven measure of latent health status, it performs robustly across the settings and populations studied. The FI-Lab thereby offers a pragmatic complement to current clinical measures and human assessment.

## Supporting information

S1 Appendix Supplementary methods, figures, and tables. (DOCX)

S1 Checklist STROBE-RECORD Checklist. **STROBE:** von Elm E, Altman DG, Egger M, Pocock SJ, Gøtzsche PC, Vandenbroucke JP, et al. (2007). The Strengthening the Reporting of Observational Studies in Epidemiology (STROBE) Statement: Guidelines for Reporting Observational Studies. PLoS Med. 4(10): e296. https://doi.org/10.1371/journal.pmed.0040296. **RECORD:** Benchimol EI, Smeeth L, Guttmann A, Harron K, Moher D, Petersen I, Sørensen HT, von Elm E, Langan SM; RECORD Working Committee. (2015). The REporting of studies Conducted using Observational Routinely-collected health Data (RECORD) statement. PLoS Med. 12(10):e1001885. https://doi.org/10.1371/journal.pmed.1001885. **Fig A:** STROBE flow diagram of the study cohort selection process. **Fig B:** FI-Lab Stability Analysis in the MIMIC-IV Database. This plot shows the median coefficient of variation (CV) of FI-Lab scores against the number of requested features, categorised by the availability of vital signs and varying feature floor thresholds (0, 5, 10, 15, and 20) within the MIMIC-IV database. The number of patients included at each specific number of features requested is indicated by the labels on the plot. **Fig C:** FI-Lab Stability Analysis in the King’s College Hospital (KCH) Database. This plot illustrates the median coefficient of variation (CV) of FI-Lab scores as the number of requested features increases, stratified by the presence or absence of vital signs and different feature floor thresholds (0, 5, 10, 15, and 20) within the KCH dataset. Labels indicate the number of patients contributing to each data point. **Fig D:** FI-Lab Score Stability Against Simulated Analytical Noise. To test robustness against laboratory measurement uncertainty, a simulation was performed on 5,000 randomly sampled patients from the KCH database. For each patient, the FI-Lab score was recalculated 20 separate times, with noise (for example, increasing or decreasing a recorded Sodium by 2 mmol/l) introduced to their laboratory values prior to each calculation. The plot shows the median coefficient of variation (CV) of these 20 scores. The results demonstrate that score stability improves (i.e., CV decreases) as the number of features increases, with the effect plateauing after 30 features. **Fig E.** Model Performance (C-Statistic) by FI-Lab Configuration Without Vital Signs in the MIMIC-IV-ED Cohort. The figure displays the predictive performance (Concordance Index/C-Statistic) of the FI-Lab across six clinical outcomes, based on laboratory data alone. Performance is shown for the ‘Common’ and ‘Importance’ test selection strategies (panels). Within each plot, the x-axis indicates the number of laboratory features used, and colours denote the minimum feature floor required to calculate a score. A key pattern, particularly for the ‘Common’ strategy, is that performance does not increase indefinitely with more features. Instead, it often peaks when using 20–40 tests and then plateaus or declines, a trend visible across outcomes like in-hospital death, ED admission, and readmissions. The ‘Importance’ strategy tends to show more consistent improvement, though performance gains often level off at higher feature counts. **Fig F.** Model Performance (C-Statistic) by FI-Lab Configuration Without Vital Signs in the UK Biobank Cohort. Predictive performance (Concordance Index/C-Statistic) of FI-Lab across four clinical outcomes, based on laboratory data alone. Performance is shown for the ‘Common’ and ‘Importance’ test-selection strategies (panels); within each, the x-axis indicates the number of laboratory features used and colours denote the minimum feature floor required to calculate a score. Unlike the ED cohorts, where the ‘Common’ strategy peaks and then plateaus or declines, performance in UK Biobank generally increases with additional features—consistent with near-universal baseline testing that is largely independent of patient acuity. **Fig G.** Predictive Performance of FI-Lab Configurations in the MIMIC-IV Database. This figure illustrates the predictive performance (Concordance Index/ C-Statistic) of various FI-Lab configurations for six clinical outcomes within the MIMIC-IV cohort. Performance is plotted against the number of features used (x-axis). Panels compare feature selection based on ‘Common’ prevalence versus ‘Importance’ ranking. Different lines represent varying minimum feature prevalence thresholds (‘Feature Floor’). The six outcomes evaluated are 1-year mortality, 1-year readmission, 30-day readmission, ED admission, in-hospital death, and length of stay. **Fig H.** Performance of FI-Lab Configurations in the King’s College Hospital (KCH) Database (with Vitals). This figure shows the predictive performance (C-Statistic) of FI-Lab configurations for six outcomes in the KCH cohort, including vital signs as potential features. Performance is shown across a range of feature counts (x-axis), stratified by the feature selection method (‘Common’ versus ‘Importance’) and the minimum feature prevalence (‘Feature Floor’). The six outcomes evaluated are 1-year mortality, 1-year readmission, 30-day readmission, ED admission, in-hospital death, and length of stay. **Fig I.** Predictive Performance of FI-Lab Configurations in the King’s College Hospital (KCH) Database (without Vitals). This figure shows the predictive performance (C-Statistic) of FI-Lab configurations for six outcomes in the KCH cohort when vital signs have been excluded from the potential feature set. Performance is shown across a range of feature counts (x-axis), stratified by the feature selection method (‘Common’ versus ‘Importance’) and the minimum feature prevalence (‘Feature Floor’). The six outcomes evaluated are 1-year mortality, 1-year readmission, 30-day readmission, ED admission, in-hospital death, and length of stay. **Fig J.** Model Fit (AIC) for FI-Lab Configurations Across Outcomes and Datasets. AIC values for FI-Lab models across outcomes and datasets. Lower AIC indicates better model fit. Rows correspond to outcomes; columns to dataset–strategy combinations. Blank UK Biobank panels indicate unavailable outcomes; UK Biobank ‘1-Year Admission’ is shown under ‘1-Year Readmission’. **Fig K.** Average Patient Coverage by FI-Lab Configuration Across Datasets. Panels show dataset and feature-selection strategy (KCH, MIMIC-IV-ED, UK Biobank; Common versus Importance). Lines show the mean proportion of eligible patients for whom an FI-Lab score could be computed (averaged across available outcomes) as the number of features increases; colours denote the minimum-tests “floor” (0, 5, 10, 15, and 20). Coverage generally increases with more features and decreases with stricter floors. With floors ≤10, coverage approaches ~95%–100% by ~30–40 features in all datasets; a 20-test floor yields lower coverage at small feature counts most notably in UK Biobank under the Importance strategy, but rises as additional features are included. **Fig L.** Hazard Ratios for 1-Year Mortality Across Datasets and Feature-Selection Strategies. Columns show datasets (KCH, MIMIC-IV-ED, UK Biobank); rows show strategies (Common, Importance). Coloured lines represent FI-Lab hazard ratios by number of laboratory features and minimum-tests “floor”; shaded bands are 95% confidence intervals (CI). Red triangles show the median Age hazard ratio with whiskers indicating the range across floors/models; grey squares show the median NEWS2 hazard ratio with range (not available in UK Biobank). The y-axis is log_2_-scaled; the dashed line marks a hazard ratio of 1. **Fig M.** Hazard Ratios for In-Hospital Death Across FI-Lab Configurations, Age, and NEWS2 in KCH and MIMIC-IV-ED. Hazard ratios for in-hospital death are shown for FI-Lab (coloured lines, shaded 95% CI) across varying numbers of features and minimum tests required, for both the common and importance-based feature selection strategies. Red triangles indicate the median hazard ratio for age with range (minimum–maximum) across feature floors; grey squares indicate the median hazard ratio for NEWS2 with range. FI-Lab estimates represent the effect within the combined model. Hazard ratios are displayed on a log_2_ scale; the dashed horizontal line indicates a hazard ratio of 1.0. **Fig N.** Hazard Ratios for Length of Stay Across FI-Lab Configurations, Age, and NEWS2 in KCH and MIMIC-IV-ED. Hazard ratios for time to hospital discharge are shown for FI-Lab (coloured lines, shaded 95% CI) across varying numbers of features and minimum tests required (“feature floor”), for both the common and importance-based feature selection strategies. Red triangles indicate the median hazard ratio for age with range (minimum–maximum) across feature floors; grey squares indicate the median hazard ratio for NEWS2 with range. FI-Lab estimates represent the effect within the combined model. Hazard ratios are displayed on a log_2_ scale; the dashed horizontal line indicates a hazard ratio of 1.0. In this Cox model, hazard ratios < 1 indicate a lower likelihood of discharge at any given time (i.e., longer stay). FI-Lab showed a stronger association with prolonged stay than age. **Fig O.** Hazard Ratios for 1-Year Readmission Across FI-Lab Configurations, Age, and NEWS2 in KCH, MIMIC-IV-ED, and UK Biobank. Hazard ratios for 1-year readmission are shown for FI-Lab (coloured lines, shaded 95% CI) across varying numbers of features and minimum tests required (“feature floor”), for both the common and importance-based feature selection strategies. In the UK Biobank dataset, the outcome “1-Year Admission” is equivalent to “1-Year Readmission” in KCH and MIMIC-IV-ED. Red triangles indicate the median hazard ratio for age with range (minimum–maximum) across feature floors; grey squares indicate the median hazard ratio for NEWS2 with range. FI-Lab estimates represent the effect within the combined model for KCH and MIMIC-IV-ED, and within the FI-Lab model for UK Biobank; NEWS2 was unavailable in UK Biobank. Hazard ratios are displayed on a log_2_ scale; the dashed horizontal line indicates a hazard ratio of 1.0. **Fig P.** Hazard Ratios for ED Admission Across FI-Lab Configurations, Age, and NEWS2 in KCH and MIMIC-IV-ED. Hazard ratios for emergency department (ED) admission are shown for FI-Lab (coloured lines, shaded 95% CI) across varying numbers of features and minimum tests required (“feature floor”), for both the common and importance-based feature selection strategies. Red triangles indicate the median hazard ratio for age with range (minimum–maximum) across feature floors; grey squares indicate the median hazard ratio for NEWS2 with range. FI-Lab estimates represent the effect within the combined model; NEWS2 was unavailable in the UK Biobank dataset. Hazard ratios are displayed on a log_2_ scale; the dashed horizontal line indicates a hazard ratio of 1.0. Higher hazard ratios indicate a greater likelihood of admission from the ED. **Table A.** FI-Lab Hazard Ratios for 1-Year Mortality by Age Group. Hazard ratios for 1-year mortality across age bands in three cohorts. Models adjusted for age (within band) and gender. FI-Lab calculated using 25 most common laboratory tests with minimum 15 tests required for score calculation. All hazard ratios *p* < 0.001. HR, hazard ratio; CI, confidence interval. **Table B.** Hazard ratios for 1 year mortality by ethnic group. Hazard ratios for 1-year mortality stratified by ethnicity and race across three cohorts. Models adjusted for age and gender. FI-Lab calculated using 25 most common laboratory tests with minimum 15 tests required for score calculation. All hazard ratios *p* < 0.001. Interaction *p*-values test for differences in FI-Lab effect across ethnic/racial groups within each cohort. HR, hazard ratio; CI, confidence interval. **Fig Q.** FI-Lab Hazard Ratios for 1-Year Mortality Stratified by Ethnicity Across Three Cohorts. Forest plot showing hazard ratios (per standard deviation increase in FI-Lab) for 1-year mortality across ethnic groups in MIMIC-IV-ED (purple), King’s College Hospital (blue), and UK Biobank (green for 1-year, yellow for 10-year mortality). Points represent hazard ratio estimates with horizontal lines indicating 95% CI. The vertical dashed line at hazard ratio = 1.0 indicates no effect. Sample sizes and event counts are displayed in the table above. Results are grouped by ethnicity (White, Asian, Black, Chinese, Mixed, Other, Hispanic/Latino, Not stated) to facilitate comparison across datasets. The x-axis is truncated at 0.68–3.7 for clarity; one confidence interval with very few events extends beyond this range and is indicated by an arrow (full numeric results are provided in the table above). UK Biobank displays both 1-year and 10-year mortality results due to low event rates in some ethnic groups at 1 year. FI-Lab demonstrates significant associations with mortality across nearly all ethnic groups, with broadly consistent hazard ratios across datasets despite variation in baseline mortality risk and population composition. The Black and Chinese ethnic groups in UK Biobank 1-year follow-up did not reach statistical significance, likely reflecting low statistical power with 8 and fewer than 5 events, respectively. **Table C.** Descriptive statistics and feature importance for the top 70 tests (including vitals) derived from the MIMIC database. Abbreviations: I, Icterus Index; H, Haemolysis Index; L, Lipaemia Index. **Table D.** Descriptive statistics and feature importance for the top 63 tests (excluding vitals) derived from the MIMIC database. Abbreviations: I, Icterus Index; H, Haemolysis Index; L, Lipaemia Index. **Table E.** Descriptive statistics and feature importance for the top 63 tests (excluding vitals) derived from UK Biobank. **Table F.** Descriptive statistics and feature importance for the top 50 tests (excluding vitals) derived from KCH. **Fig R.** Relationship between test frequency and abnormality rate in emergency department patients. Scatter plot showing the proportion of abnormal results versus total test frequency (log scale) for blood tests from emergency department attendances in the MIMIC-IV-ED cohort. Each point represents a unique laboratory test, colored by abnormality rate band (grey: no abnormal flags; dark purple to green: increasing proportions of abnormal results). Vertical dashed lines indicate frequency cutoffs for the top 30 and top 70 most commonly ordered tests. Commonly ordered tests (right side) demonstrate a range of abnormality rates, typically 10%–50%. Notably, very high-abnormality rates (>70%) are observed exclusively among infrequently ordered tests (left side), such as CD4/CD8 ratio (76% abnormal), Free Kappa (75%), and NTproBNP (70%). These high-abnormality, low-frequency tests represent investigations ordered only when clinicians have high pre-test probability of a specific condition, resulting in substantial enrichment for abnormal results compared to the baseline prevalence in the general emergency department population. **Table G.** Hazard ratios (95% CI) for mortality per SD-increase in FI-Lab within strata of the Williams and colleagues frailty index at baseline. **Table H**. Hazard ratios (95% CI) for mortality per SD-increase in FI-Lab across baseline levels of the adapted Frailty Phenotype. **Table I**. Hazard ratios (95% CI) for 10-year mortality at specified levels of FI-Lab and Williams and colleagues frailty index in UK Biobank—both variables in same model adjusted for age and sex.
(DOCX)

## Abbreviations

APACHE: Acute Physiology and Chronic Health Evaluation
CI: confidence intervals
COV: coefficient of variation
ED: emergency department
EHRs: Electronic health records
FI-Lab: laboratory-based frailty index
KCH: King’s College Hospital
ML: machine learning
NEWS2: National Early Warning Score 2
RECORD: Reporting of studies Conducted using Observational Routinely-collected health Data
STROBE: Strengthening the Reporting of Observational Studies in Epidemiology.
