# Supplementary material for: Evaluation of the FI-Lab, a laboratory-based, automated frailty index for acute care: A multicohort study
Source: PLoS Med. 2026 Sep 1;23(9):e1005004. doi: 10.1371/journal.pmed.1005004 (PMC13577487; doi:10.1371/journal.pmed.1005004)
Supplement: Supplementary file 1 [file pmed.1005004.s001.docx]

**Supplementary Figure A: STROBE flow diagram of the study cohort selection process.**

**Supplementary Figure B: FI-Lab Stability Analysis in the MIMIC-IV Database.**

*This plot shows the median coefficient of variation (CV) of FI-Lab scores against the number of requested features, categorized by the availability of vital signs and varying feature floor thresholds (0, 5, 10, 15, and 20) within the MIMIC-IV database. The number of patients included at each specific number of features requested is indicated by the labels on the plot.*

**Supplementary Figure C: FI-Lab Stability Analysis in the King's College Hospital (KCH) Database.** *This plot illustrates the median coefficient of variation (CV) of FI-Lab scores as the number of requested features increases, stratified by the presence or absence of vital signs and different feature floor thresholds (0, 5, 10, 15, and 20) within the KCH dataset. Labels indicate the number of patients contributing to each data point.*

**Supplementary Figure D: FI-Lab Score Stability Against Simulated Analytical Noise.**

*To test robustness against laboratory measurement uncertainty, a simulation was performed on 5,000 randomly sampled patients from the KCH database. For each patient, the FI-Lab score was recalculated 20 separate times, with noise (for example increasing or decreasing a recorded Sodium by 2 mmol/l) introduced to their laboratory values prior to each calculation. The plot shows the median coefficient of variation (CV) of these 20 scores. The results demonstrate that score stability improves (i.e., CV decreases) as the number of features increases, with the effect plateauing after 30 features.*

**Supplementary Figure E. Model Performance (C-Statistic) by FI-Lab Configuration Without Vital Signs in the MIMIC-IV-ED Cohort.**

*The figure displays the predictive performance (Concordance Index/C-Statistic) of the FI-Lab across six clinical outcomes, based on laboratory data alone. Performance is shown for the 'Common' and 'Importance' test selection strategies (panels). Within each plot, the x-axis indicates the number of laboratory features used, and colours denote the minimum feature floor required to calculate a score. A key pattern, particularly for the 'Common' strategy, is that performance does not increase indefinitely with more features. Instead, it often peaks when using 20-40 tests and then plateaus or declines, a trend visible across outcomes like in-hospital death, ED admission, and readmissions. The 'Importance' strategy tends to show more consistent improvement, though performance gains often level off at higher feature counts.*

**Supplementary Figure F. Model Performance (C-Statistic) by FI-Lab Configuration Without Vital Signs in the UK Biobank Cohort.**

*Predictive performance (Concordance Index/C-Statistic) of FI-Lab across four clinical outcomes, based on laboratory data alone. Performance is shown for the 'Common' and 'Importance' test-selection strategies (panels); within each, the x-axis indicates the number of laboratory features used and colours denote the minimum feature floor required to calculate a score. Unlike the ED cohorts, where the 'Common' strategy peaks and then plateaus or declines, performance in UK Biobank generally increases with additional features — consistent with near-universal baseline testing that is largely independent of patient acuity.*

**Supplementary Figure G. Predictive Performance of FI-Lab Configurations in the MIMIC-IV Database.**

*This figure illustrates the predictive performance (Concordance Index / C-Statistic) of various FI-Lab configurations for six clinical outcomes within the MIMIC-IV cohort. Performance is plotted against the number of features used (x-axis). Panels compare feature selection based on 'Common' prevalence versus 'Importance' ranking. Different lines represent varying minimum feature prevalence thresholds ('Feature Floor'). The six outcomes evaluated are 1-year mortality, 1-year readmission, 30-day readmission, ED admission, in-hospital death, and length of stay.*

**Supplementary Figure H. Performance of FI-Lab Configurations in the King's College Hospital (KCH) Database (with Vitals).**

*This figure shows the predictive performance (C-Statistic) of FI-Lab configurations for six outcomes in the KCH cohort, including vital signs as potential features. Performance is shown across a range of feature counts (x-axis), stratified by the feature selection method ('Common' vs. 'Importance') and the minimum feature prevalence ('Feature Floor'). The six outcomes evaluated are 1-year mortality, 1-year readmission, 30-day readmission, ED admission, in-hospital death, and length of stay.*

**Supplementary Figure I. Predictive Performance of FI-Lab Configurations in the King's College Hospital (KCH) Database (without Vitals).**

*This figure shows the predictive performance (C-Statistic) of FI-Lab configurations for six outcomes in the KCH cohort when vital signs have been excluded from the potential feature set. Performance is shown across a range of feature counts (x-axis), stratified by the feature selection method ('Common' vs. 'Importance') and the minimum feature prevalence ('Feature Floor'). The six outcomes evaluated are 1-year mortality, 1-year readmission, 30-day readmission, ED admission, in-hospital death, and length of stay.*

**Supplementary Figure J. Model Fit (AIC) for FI-Lab Configurations Across Outcomes and Datasets**

*AIC values for FI-Lab models across outcomes and datasets. Lower AIC indicates better model fit. Rows correspond to outcomes; columns to dataset–strategy combinations. Blank UK Biobank panels indicate unavailable outcomes; UK Biobank ‘1-Year Admission’ is shown under ‘1-Year Readmission’.*

**Supplementary Figure K.** Average Patient Coverage by FI-Lab Configuration Across Datasets.

*Panels show dataset and feature-selection strategy (KCH, MIMIC-IV-ED, UK Biobank; Common vs. Importance). Lines show the mean proportion of eligible patients for whom an FI-Lab score could be computed (averaged across available outcomes) as the number of features increases; colours denote the minimum-tests “floor” (0, 5, 10, 15, 20). Coverage generally increases with more features and decreases with stricter floors. With floors ≤10, coverage approaches ~95–100% by ~30–40 features in all datasets; a 20-test floor yields lower coverage at small feature counts most notably in UK Biobank under the Importance strategy, but rises as additional features are included.*

**Supplementary Figure L*.* Hazard Ratios for 1-Year Mortality Across Datasets and Feature-Selection Strategies.**

*Columns show datasets (KCH, MIMIC-IV-ED, UK Biobank); rows show strategies (Common, Importance). Coloured lines represent FI-Lab hazard ratios by number of laboratory features and minimum-tests “floor”; shaded bands are 95% confidence intervals. Red triangles show the median Age hazard ratio with whiskers indicating the range across floors/models; grey squares show the median NEWS2 hazard ratio with range (not available in UK Biobank). The y-axis is log₂-scaled; the dashed line marks a hazard ratio of 1.*

**Supplementary Figure M. Hazard Ratios for In-Hospital Death Across FI-Lab Configurations, Age, and NEWS2 in KCH and MIMIC-IV-ED**

*Hazard ratios for in-hospital death are shown for FI-Lab (coloured lines, shaded 95% confidence intervals) across varying numbers of features and minimum tests required, for both the common and importance-based feature selection strategies. Red triangles indicate the median hazard ratio for age with range (minimum–maximum) across feature floors; grey squares indicate the median hazard ratio for NEWS2 with range. FI-Lab estimates represent the effect within the combined model. Hazard ratios are displayed on a log₂ scale; the dashed horizontal line indicates a hazard ratio of 1.0.*

**Supplementary Figure N. Hazard Ratios for Length of Stay Across FI-Lab Configurations, Age, and NEWS2 in KCH and MIMIC-IV-ED**

*Hazard ratios for time to hospital discharge are shown for FI-Lab (coloured lines, shaded 95% confidence intervals) across varying numbers of features and minimum tests required (“feature floor”), for both the common and importance-based feature selection strategies. Red triangles indicate the median hazard ratio for age with range (minimum–maximum) across feature floors; grey squares indicate the median hazard ratio for NEWS2 with range. FI-Lab estimates represent the effect within the combined model. Hazard ratios are displayed on a log₂ scale; the dashed horizontal line indicates a hazard ratio of 1.0. In this Cox model, hazard ratios < 1 indicate a lower likelihood of discharge at any given time (i.e., longer stay). FI-Lab showed a stronger association with prolonged stay than age.*

**Supplementary Figure O.** **Hazard Ratios for 1-Year Readmission Across FI-Lab Configurations, Age, and NEWS2 in KCH, MIMIC-IV-ED, and UK Biobank**

*Hazard ratios for 1-year readmission are shown for FI-Lab (coloured lines, shaded 95% confidence intervals) across varying numbers of features and minimum tests required (“feature floor”), for both the common and importance-based feature selection strategies. In the UK Biobank dataset, the outcome “1-Year Admission” is equivalent to “1-Year Readmission” in KCH and MIMIC-IV-ED. Red triangles indicate the median hazard ratio for age with range (minimum–maximum) across feature floors; grey squares indicate the median hazard ratio for NEWS2 with range. FI-Lab estimates represent the effect within the combined model for KCH and MIMIC-IV-ED, and within the FI-Lab model for UK Biobank; NEWS2 was unavailable in UK Biobank. Hazard ratios are displayed on a log₂ scale; the dashed horizontal line indicates a hazard ratio of 1.0.*

**Supplementary Figure P.** **Hazard Ratios for ED Admission Across FI-Lab Configurations, Age, and NEWS2 in KCH and MIMIC-IV-ED**

*Hazard ratios for emergency department (ED) admission are shown for FI-Lab (coloured lines, shaded 95% confidence intervals) across varying numbers of features and minimum tests required (“feature floor”), for both the common and importance-based feature selection strategies. Red triangles indicate the median hazard ratio for age with range (minimum–maximum) across feature floors; grey squares indicate the median hazard ratio for NEWS2 with range. FI-Lab estimates represent the effect within the combined model; NEWS2 was unavailable in the UK Biobank dataset. Hazard ratios are displayed on a log₂ scale; the dashed horizontal line indicates a hazard ratio of 1.0. Higher hazard ratios indicate a greater likelihood of admission from the ED.*

| **Dataset** | **Age Band** | **N** | **Events** | **HR (95% CI)** | **C-index** |
| --- | --- | --- | --- | --- | --- |
| **MIMIC-IV-ED** |  |  |  |  |  |
|  | <30 | 27,169 | 222 | 2.26 (2.05, 2.49) | 0.821 |
|  | 30-40 | 23,836 | 437 | 2.37 (2.20, 2.55) | 0.785 |
|  | 40-50 | 25,342 | 1,027 | 2.45 (2.33, 2.58) | 0.79 |
|  | 50-60 | 35,891 | 2,754 | 2.62 (2.53, 2.71) | 0.791 |
|  | 60-70 | 36,109 | 4,549 | 2.32 (2.26, 2.39) | 0.75 |
|  | 70-80 | 29,819 | 5,272 | 2.16 (2.10, 2.22) | 0.719 |
|  | 80+ | 31,570 | 8,627 | 1.77 (1.73, 1.81) | 0.672 |
| **King's College Hospital** |  |  |  |  |  |
|  | <30 | 25,381 | 74 | 2.97 (2.57, 3.42) | 0.896 |
|  | 30-40 | 23,749 | 120 | 3.16 (2.82, 3.54) | 0.918 |
|  | 40-50 | 21,513 | 241 | 2.99 (2.75, 3.26) | 0.889 |
|  | 50-60 | 23,689 | 639 | 2.82 (2.66, 2.99) | 0.832 |
|  | 60-70 | 16,669 | 997 | 2.59 (2.46, 2.72) | 0.806 |
|  | 70-80 | 14,495 | 1,581 | 2.27 (2.17, 2.37) | 0.75 |
|  | 80+ | 15,847 | 2,572 | 2.11 (2.03, 2.19) | 0.71 |
| **UK Biobank** |  |  |  |  |  |
|  | 40-50 | 111,623 | 80 | 1.90 (1.66, 2.19) | 0.714 |
|  | 50-60 | 158,785 | 213 | 2.11 (1.96, 2.26) | 0.756 |
|  | 60-70 | 204,603 | 579 | 1.78 (1.70, 1.87) | 0.729 |
|  | N/A | 2,307 | 8 | 1.79 (1.25, 2.56) | 0.736 |

**Supplementary Table A. FI-Lab Hazard Ratios for 1-Year Mortality by Age Group**

*Hazard ratios for one-year mortality across age bands in three cohorts. Models adjusted for age (within band) and gender. FI-Lab calculated using 25 most common laboratory tests with minimum 15 tests required for score calculation. All hazard ratios p<0.001. HR, hazard ratio; CI, confidence interval.*

| **Dataset** | **Group** | **N** | **Events** | **HR (95% CI)** | **C-index** | **Interaction p** |
| --- | --- | --- | --- | --- | --- | --- |
| **MIMIC-IV-ED** |  |  |  |  |  | <0.001 |
|  | White | 67,261 | 5,993 | 1.93 (1.88, 1.98) | 0.788 |  |
|  | Black/African American | 16,195 | 669 | 2.12 (1.97, 2.29) | 0.824 |  |
|  | Hispanic/Latino | 6,530 | 154 | 2.30 (1.96, 2.72) | 0.828 |  |
|  | Asian | 5,139 | 261 | 1.95 (1.73, 2.21) | 0.832 |  |
|  | Other | 6,780 | 315 | 2.03 (1.82, 2.26) | 0.82 |  |
| **King's College Hospital** |  |  |  |  |  | <0.001 |
|  | White | 41,005 | 1,721 | 2.21 (2.13, 2.30) | 0.896 |  |
|  | Black or Black British | 26,122 | 567 | 2.27 (2.13, 2.42) | 0.901 |  |
|  | Asian or Asian British | 3,687 | 102 | 2.88 (2.42, 3.41) | 0.916 |  |
|  | Other ethnic group | 7,729 | 132 | 2.52 (2.21, 2.88) | 0.923 |  |
|  | Mixed | 2,591 | 39 | 2.51 (1.93, 3.27) | 0.93 |  |
|  | Prefer not to answer | 9,083 | 288 | 2.48 (2.25, 2.72) | 0.916 |  |
|  | Chinese | 519 | 22 | 2.16 (1.52, 3.06) | 0.907 |  |
| **UK Biobank 1 year** |  |  |  |  |  | 0.18 |
|  | White | 449,967 | 844 | 1.88 (1.80, 1.95) | 0.774 |  |
|  | Asian or Asian British | 9,216 | 11 | 1.50 (1.02, 2.20) | 0.652 |  |
|  | Mixed | 2,795 | 7 | 2.09 (1.41, 3.09) | 0.84 |  |
|  | Black or Black British | 7,377 | 8 | 1.22 (0.73, 2.05) | 0.611 |  |
|  | Other ethnic group | 4,252 | <5 | 2.17 (1.27, 3.71) | 0.856 |  |
|  | Chinese | 1,479 | <5 | 0.64 (0.12, 3.40) | 0.901 |  |
|  | Not stated | 2,232 | <5 | 2.35 (1.34, 4.12) | 0.859 |  |
| **UK Biobank 10 year** |  |  |  |  |  | <0.001 |
|  | White | 449,967 | 22034 | 1.41 (1.40, 1.43) | 0.721 |  |
|  | Asian or Asian British | 9,216 | 296 | 1.33 (1.22, 1.44) | 0.75 |  |
|  | Mixed | 2,795 | 93 | 1.39 (1.18, 1.64) | 0.764 |  |
|  | Black or Black British | 7,377 | 232 | 1.24 (1.12, 1.36) | 0.691 |  |
|  | Other ethnic group | 4,252 | 148 | 1.24 (1.10, 1.41) | 0.856 |  |
|  | Chinese | 1,479 | 36 | 1.24 (0.95, 1.63) | 0.901 |  |
|  | Not stated | 2,232 | 154 | 1.57 (1.40, 1.77) | 0.859 |  |

**Supplementary Table B. Hazard ratios for one year mortality by ethnic group**

*Hazard ratios for one-year mortality stratified by ethnicity and race across three cohorts. Models adjusted for age and gender. FI-Lab calculated using 25 most common laboratory tests with minimum 15 tests required for score calculation. All hazard ratios p<0.001. Interaction p-values test for differences in FI-Lab effect across ethnic/racial groups within each cohort. HR, hazard ratio; CI, confidence interval.*

**Supplementary Figure Q. FI-Lab Hazard Ratios for One-Year Mortality Stratified by Ethnicity Across Three Cohorts.**

*Forest plot showing hazard ratios (per standard deviation increase in FI-Lab) for one-year mortality across ethnic groups in MIMIC-IV-ED (purple), King's College Hospital (blue), and UK Biobank (green for one-year, yellow for ten-year mortality). Points represent hazard ratio estimates with horizontal lines indicating 95% confidence intervals. The vertical dashed line at hazard ratio = 1.0 indicates no effect. Sample sizes and event counts are displayed in the table above. Results are grouped by ethnicity (White, Asian, Black, Chinese, Mixed, Other, Hispanic/Latino, Not stated) to facilitate comparison across datasets. The x-axis is truncated at 0.68-3.7 for clarity; one confidence interval with very few events extends beyond this range and is indicated by an arrow (full numeric results are provided in the table above). UK Biobank displays both one-year and ten-year mortality results due to low event rates in some ethnic groups at one year. FI-Lab demonstrates significant associations with mortality across nearly all ethnic groups, with broadly consistent hazard ratios across datasets despite variation in baseline mortality risk and population composition. The Black and Chinese ethnic groups in UK Biobank one-year follow-up did not reach statistical significance, likely reflecting low statistical power with 8 and fewer than 5 events, respectively.*

| **Test Name** | **Patients tested** | **Percent of all patients** | **Percent of abnormal results** | **Mean Value** | **Importance score** |
| --- | --- | --- | --- | --- | --- |
| Systolic BP | 220737 | 96.9 | 33.8 | 133 | 0.0581 |
| Heart Rate | 220835 | 97.0 | 22.3 | 83.4 | 0.0578 |
| Respiratory Rate | 220641 | 96.9 | 8.15 | 17.7 | 0.0574 |
| Diastolic BP | 220737 | 96.9 | 28.6 | 77.6 | 0.0574 |
| Temperature | 216769 | 95.2 | 7.97 | 36.7 | 0.057 |
| SpO2 | 216024 | 94.9 | 5.31 | 98.2 | 0.0558 |
| RDW | 213005 | 93.5 | 18.4 | 14.2 | 0.0443 |
| MCV | 213000 | 93.5 | 17.9 | 90.1 | 0.0442 |
| Urea Nitrogen | 212503 | 93.3 | 29.6 | 19.5 | 0.0441 |
| MCH | 212991 | 93.5 | 24.7 | 29.6 | 0.0439 |
| White Blood Cells | 213051 | 93.6 | 31 | 9.12 | 0.043 |
| Creatinine | 212865 | 93.5 | 20.9 | 1.17 | 0.0429 |
| Anion Gap | 210362 | 92.4 | 10.1 | 15.5 | 0.0423 |
| Glucose | 210310 | 92.3 | 61 | 126 | 0.0418 |
| Hematocrit | 213130 | 93.6 | 39.9 | 37.8 | 0.0417 |
| Platelet Count | 213007 | 93.5 | 16.2 | 244 | 0.0417 |
| Hemoglobin | 212997 | 93.5 | 44.7 | 12.4 | 0.0415 |
| Neutrophils | 204581 | 89.8 | 48.7 | 67.8 | 0.0415 |
| Bicarbonate | 210443 | 92.4 | 19.6 | 24.4 | 0.0412 |
| MCHC | 212998 | 93.5 | 24.8 | 32.9 | 0.0412 |
| Potassium | 210513 | 92.4 | 12.1 | 4.29 | 0.0409 |
| Lymphocytes | 204570 | 89.8 | 45.9 | 22.2 | 0.0408 |
| Monocytes | 204579 | 89.8 | 12.4 | 6.98 | 0.0408 |
| Eosinophils | 204559 | 89.8 | 28.7 | 1.87 | 0.0406 |
| Sodium | 210542 | 92.5 | 9.58 | 138 | 0.0406 |
| Chloride | 210505 | 92.4 | 13.2 | 101 | 0.0404 |
| Basophils | 204565 | 89.8 | 2.87 | 0.514 | 0.0403 |
| Red Blood Cells | 213007 | 93.5 | 49 | 4.22 | 0.0398 |
| pH | 140672 | 61.8 | 1.06 | 6.34 | 0.0333 |
| Specific Gravity | 140679 | 61.8 | 5.16 | 1.02 | 0.0333 |
| Protein | 140674 | 61.8 | 18.8 | 71 | 0.0328 |
| RDW-SD | 121766 | 53.5 | 39.3 | 46.4 | 0.0313 |
| Absolute Neutrophil Count | 117564 | 51.6 | 43.3 | 6.41 | 0.0307 |
| Absolute Eosinophil Count | 117553 | 51.6 | 28.6 | 0.142 | 0.0305 |
| Absolute Monocyte Count | 117556 | 51.6 | 29.7 | 0.691 | 0.0305 |
| Absolute Basophil Count | 117551 | 51.6 | 10.2 | 0.041 | 0.0304 |
| Absolute Lymphocyte Count | 117537 | 51.6 | 34.2 | 1.83 | 0.0304 |
| Immature Granulocytes | 110341 | 48.5 | 11.7 | 0.495 | 0.0294 |
| PT | 110266 | 48.4 | 39.9 | 14.9 | 0.0294 |
| Magnesium | 109931 | 48.3 | 9.44 | 1.96 | 0.0293 |
| PTT | 109046 | 47.9 | 23.4 | 33.2 | 0.0293 |
| INR(PT) | 110266 | 48.4 | 41.2 | 1.37 | 0.0292 |
| Calcium, Total | 109129 | 47.9 | 19.2 | 9 | 0.0292 |
| Phosphate | 108354 | 47.6 | 24.2 | 3.56 | 0.0291 |
| RBC | 101588 | 44.6 | 34.9 | 7.88 | 0.0283 |
| Epithelial Cells | 101578 | 44.6 | 0 | 2.54 | 0.0283 |
| WBC | 101600 | 44.6 | 29.6 | 12.2 | 0.0282 |
| I | 95674 | 42.0 | 0 | 1.01 | 0.0274 |
| H | 95672 | 42.0 | 0.00314 | 62.2 | 0.0274 |
| L | 95682 | 42.0 | 0.00105 | 14.1 | 0.0274 |
| Alanine Aminotransferase (ALT) | 91840 | 40.3 | 20.6 | 49.4 | 0.0269 |
| Asparate Aminotransferase (AST) | 91834 | 40.3 | 28.3 | 61.5 | 0.0268 |
| Bilirubin, Total | 91554 | 40.2 | 9.07 | 0.908 | 0.0268 |
| Alkaline Phosphatase | 91250 | 40.1 | 25 | 108 | 0.0268 |
| Lactate | 84705 | 37.2 | 28.4 | 1.91 | 0.0259 |
| Albumin | 82055 | 36.0 | 21.2 | 3.94 | 0.0254 |
| Ketone | 140670 | 61.8 | 6.32 | 33.4 | 0.025 |
| Urobilinogen | 140686 | 61.8 | 11.7 | 2.46 | 0.0246 |
| Lipase | 60313 | 26.5 | 15.8 | 82.7 | 0.0218 |
| Troponin T | 60845 | 26.7 | 22.8 | 0.203 | 0.0189 |
| Potassium, Whole Blood | 27033 | 11.9 | 27 | 4.52 | 0.0145 |
| Glucose | 140666 | 61.8 | 3.62 | 547 | 0.0137 |
| Creatine Kinase (CK) | 23687 | 10.4 | 31.7 | 691 | 0.0136 |
| Hyaline Casts | 25552 | 11.2 | 99.9 | 10.5 | 0.0135 |
| Lactate Dehydrogenase (LD) | 22400 | 9.8 | 42.5 | 339 | 0.0133 |
| pH | 21856 | 9.6 | 47.5 | 7.36 | 0.0131 |
| Creatine Kinase, MB Isoenzyme | 22862 | 10.0 | 11.4 | 8.3 | 0.0125 |
| Ethanol | 35937 | 15.8 | 18.6 | 204 | 0.00798 |
| Acetaminophen | 37243 | 16.4 | 2.46 | 18.4 | 0.00005 |

**Supplementary Table C. Descriptive statistics and feature importance for the top 70 tests (including vitals) derived from the MIMIC database.**

**Abbreviations**: **I**, Icterus Index; **H**, Hemolysis Index; **L**, Lipemia Index.

| Test Name | Number of Patients | Percentage of Patients | Percentage Abnormal | Mean Value | Importance Score |
| --- | --- | --- | --- | --- | --- |
| RDW | 213005 | 93.5 | 18.4 | 14.2 | 0.0545 |
| MCV | 213000 | 93.5 | 17.9 | 90.1 | 0.0542 |
| MCH | 212991 | 93.5 | 24.7 | 29.6 | 0.0536 |
| Urea Nitrogen | 212503 | 93.3 | 29.6 | 19.5 | 0.0533 |
| White Blood Cells | 213051 | 93.6 | 31 | 9.12 | 0.0528 |
| Hematocrit | 213130 | 93.6 | 39.9 | 37.8 | 0.0517 |
| Glucose | 210310 | 92.3 | 61 | 126 | 0.0516 |
| Potassium | 210513 | 92.4 | 12.1 | 4.29 | 0.0513 |
| MCHC | 212998 | 93.5 | 24.8 | 32.9 | 0.0509 |
| Neutrophils | 204581 | 89.8 | 48.7 | 67.8 | 0.0508 |
| Creatinine | 212865 | 93.5 | 20.9 | 1.17 | 0.0506 |
| Platelet Count | 213007 | 93.5 | 16.2 | 244 | 0.0506 |
| Anion Gap | 210362 | 92.4 | 10.1 | 15.5 | 0.0503 |
| Bicarbonate | 210443 | 92.4 | 19.6 | 24.4 | 0.0501 |
| Chloride | 210505 | 92.4 | 13.2 | 101 | 0.0501 |
| Monocytes | 204579 | 89.8 | 12.4 | 6.98 | 0.0499 |
| Hemoglobin | 212997 | 93.5 | 44.7 | 12.4 | 0.0498 |
| Lymphocytes | 204570 | 89.8 | 45.9 | 22.2 | 0.0497 |
| Sodium | 210542 | 92.5 | 9.58 | 138 | 0.0495 |
| Red Blood Cells | 213007 | 93.5 | 49 | 4.22 | 0.0491 |
| Eosinophils | 204559 | 89.8 | 28.7 | 1.87 | 0.049 |
| Basophils | 204565 | 89.8 | 2.87 | 0.514 | 0.0489 |
| pH | 140672 | 61.8 | 1.06 | 6.34 | 0.0403 |
| Specific Gravity | 140679 | 61.8 | 5.16 | 1.02 | 0.0403 |
| Protein | 140674 | 61.8 | 18.8 | 71 | 0.04 |
| Ketone | 140670 | 61.8 | 6.32 | 33.4 | 0.0392 |
| Urobilinogen | 140686 | 61.8 | 11.7 | 2.46 | 0.0391 |
| RDW-SD | 121766 | 53.5 | 39.3 | 46.4 | 0.0378 |
| Absolute Neutrophil Count | 117564 | 51.6 | 43.3 | 6.41 | 0.0368 |
| Absolute Eosinophil Count | 117553 | 51.6 | 28.6 | 0.142 | 0.0368 |
| Absolute Lymphocyte Count | 117537 | 51.6 | 34.2 | 1.83 | 0.0368 |
| Absolute Basophil Count | 117551 | 51.6 | 10.2 | 0.041 | 0.0368 |
| Absolute Monocyte Count | 117556 | 51.6 | 29.7 | 0.691 | 0.0367 |
| PTT | 109046 | 47.9 | 23.4 | 33.2 | 0.0356 |
| Immature Granulocytes | 110341 | 48.5 | 11.7 | 0.495 | 0.0356 |
| PT | 110266 | 48.4 | 39.9 | 14.9 | 0.0355 |
| INR(PT) | 110266 | 48.4 | 41.2 | 1.37 | 0.0354 |
| Calcium, Total | 109129 | 47.9 | 19.2 | 9 | 0.0354 |
| Magnesium | 109931 | 48.3 | 9.44 | 1.96 | 0.0353 |
| Phosphate | 108354 | 47.6 | 24.2 | 3.56 | 0.0351 |
| Glucose | 140666 | 61.8 | 3.62 | 547 | 0.0347 |
| Epithelial Cells | 101578 | 44.6 | 0 | 2.54 | 0.0341 |
| WBC | 101600 | 44.6 | 29.6 | 12.2 | 0.0341 |
| RBC | 101588 | 44.6 | 34.9 | 7.88 | 0.0341 |
| L | 95682 | 42.0 | 0.00105 | 14.1 | 0.0331 |
| I | 95674 | 42.0 | 0 | 1.01 | 0.0331 |
| H | 95672 | 42.0 | 0.00314 | 62.2 | 0.0331 |
| Alanine Aminotransferase (ALT) | 91840 | 40.3 | 20.6 | 49.4 | 0.0324 |
| Asparate Aminotransferase (AST) | 91834 | 40.3 | 28.3 | 61.5 | 0.0323 |
| Alkaline Phosphatase | 91250 | 40.1 | 25 | 108 | 0.0323 |
| Bilirubin, Total | 91554 | 40.2 | 9.07 | 0.908 | 0.0322 |
| Lactate | 84705 | 37.2 | 28.4 | 1.91 | 0.0311 |
| Albumin | 82055 | 36.0 | 21.2 | 3.94 | 0.0305 |
| Lipase | 60313 | 26.5 | 15.8 | 82.7 | 0.0262 |
| Troponin T | 60845 | 26.7 | 22.8 | 0.203 | 0.0247 |
| Ethanol | 35937 | 15.8 | 18.6 | 204 | 0.018 |
| Potassium, Whole Blood | 27033 | 11.9 | 27 | 4.52 | 0.0176 |
| Hyaline Casts | 25552 | 11.2 | 99.9 | 10.5 | 0.0171 |
| Creatine Kinase (CK) | 23687 | 10.4 | 31.7 | 691 | 0.0165 |
| Lactate Dehydrogenase (LD) | 22400 | 9.8 | 42.5 | 339 | 0.016 |
| pH | 21856 | 9.6 | 47.5 | 7.36 | 0.0158 |
| Creatine Kinase, MB Isoenzyme | 22862 | 10.0 | 11.4 | 8.3 | 0.0157 |
| Acetaminophen | 37243 | 16.4 | 2.46 | 18.4 | 0.00022 |

**Supplementary Table D. Descriptive statistics and feature importance for the top 63 tests (excluding vitals) derived from the MIMIC database.**

**Abbreviations**: **I**, Icterus Index; **H**, Hemolysis Index; **L**, Lipemia Index.

| Test | Number of Patients | Percentage of Patients | Percentage Abnormal | Commonness rank | Importance Rank |
| --- | --- | --- | --- | --- | --- |
| Haemoglobin concentration | 478200 | 95.2 | 7.9 | 1 | 14 |
| Haematocrit percentage | 478200 | 95.2 | 8.7 | 2 | 40 |
| Red blood cell (erythrocyte) count | 478200 | 95.2 | 6.9 | 3 | 41 |
| Red blood cell (erythrocyte) distribution width | 478198 | 95.2 | 5.6 | 4 | 2 |
| Mean corpuscular volume | 478198 | 95.2 | 19.7 | 5 | 20 |
| Mean corpuscular haemoglobin | 478197 | 95.2 | 17.8 | 6 | 16 |
| Platelet count | 478197 | 95.2 | 7.2 | 7 | 28 |
| Mean corpuscular haemoglobin concentration | 478193 | 95.2 | 24.3 | 8 | 42 |
| Mean platelet (thrombocyte) volume | 478192 | 95.2 | 7.6 | 9 | 38 |
| Eosinophill percentage | 477317 | 95.0 | 8.4 | 10 | 27 |
| Basophill percentage | 477317 | 95.0 | 10.0 | 11 | 30 |
| Monocyte percentage | 477317 | 95.0 | 13.2 | 12 | 37 |
| Neutrophill percentage | 477317 | 95.0 | 7.0 | 13 | 44 |
| Lymphocyte percentage | 477317 | 95.0 | 9.1 | 14 | 50 |
| Basophill count | 477311 | 95.0 | 30.4 | 15 | 23 |
| Eosinophill count | 477311 | 95.0 | 4.2 | 16 | 31 |
| Monocyte count | 477311 | 95.0 | 1.3 | 17 | 39 |
| Lymphocyte count | 477311 | 95.0 | 0.8 | 18 | 47 |
| Neutrophill count | 477311 | 95.0 | 4.3 | 19 | 49 |
| Reticulocyte count | 469878 | 93.5 | 4.9 | 20 | 46 |
| Immature reticulocyte fraction | 469877 | 93.5 | 12.9 | 21 | 36 |
| Mean reticulocyte volume | 469877 | 93.5 | 32.7 | 22 | 45 |
| Alkaline phosphatase | 469628 | 93.5 | 3.2 | 23 | 33 |
| Cholesterol | 469615 | 93.4 | 72.2 | 24 | 26 |
| Cystatin C | 469584 | 93.4 | 6.2 | 25 | 48 |
| Alanine aminotransferase | 469427 | 93.4 | 3.3 | 26 | 3 |
| Creatinine | 469383 | 93.4 | 10.3 | 27 | 32 |
| Gamma glutamyltransferase | 469368 | 93.4 | 26.0 | 28 | 34 |
| Urea | 469297 | 93.4 | 10.0 | 29 | 25 |
| Triglycerides | 469240 | 93.4 | 40.1 | 30 | 13 |
| Urate | 469051 | 93.3 | 14.4 | 31 | 8 |
| LDL direct | 468732 | 93.3 | 72.8 | 32 | 6 |
| C-reactive protein | 468594 | 93.2 | 11.6 | 33 | 43 |
| Aspartate aminotransferase | 467822 | 93.1 | 12.8 | 34 | 24 |
| Total bilirubin | 467586 | 93.0 | 2.8 | 35 | 29 |
| Apolipoprotein B | 467232 | 93.0 | 0.0 | 36 | 5 |
| IGF-1 | 467066 | 92.9 | 0.5 | 37 | 12 |
| Glycated haemoglobin (HbA1c) | 466530 | 92.8 | 8.1 | 38 | 35 |
| Vitamin D | 448376 | 89.2 | 55.4 | 39 | 22 |
| Albumin | 430098 | 85.6 | 0.1 | 40 | 21 |
| Calcium | 429955 | 85.6 | 1.8 | 41 | 9 |
| HDL cholesterol | 429894 | 85.5 | 69.2 | 42 | 19 |
| Total protein | 429627 | 85.5 | 1.3 | 43 | 17 |
| Glucose | 429590 | 85.5 | 20.7 | 44 | 11 |
| Phosphate | 429274 | 85.4 | 5.0 | 45 | 15 |
| Apolipoprotein A | 427532 | 85.1 | 3.9 | 46 | 18 |
| SHBG | 425869 | 84.7 | 8.5 | 47 | 10 |
| Direct bilirubin | 398634 | 79.3 | 0.8 | 48 | 7 |
| Lipoprotein A | 375654 | 74.8 | 21.5 | 49 | 4 |
| Rheumatoid factor | 41316 | 8.2 | 39.4 | 50 | 1 |

**Supplementary Table E**. **Descriptive statistics and feature importance for the top 63 tests (excluding vitals) derived from UK Biobank.**

| **Test** | **Number of Patients** | **Percentage of Patients** | **Percentage Abnormal** | **Commonness rank** | **Importance Rank** |
| --- | --- | --- | --- | --- | --- |
| Creatinine | 28782 | 36 | 11.4 | 1 | 9 |
| WBC | 28117 | 35.2 | 22.8 | 2 | 2 |
| MCV | 28098 | 35.1 | 39.4 | 3 | 20 |
| MPV | 28090 | 35.1 | 16.8 | 4 | 19 |
| PCV | 28084 | 35.1 | 27.3 | 5 | 4 |
| MCH | 28084 | 35.1 | 9.1 | 6 | 8 |
| Hb | 28084 | 35.1 | 27.3 | 7 | 13 |
| PLT | 28084 | 35.1 | 10.4 | 8 | 14 |
| Neutrophils | 28064 | 35.1 | 40 | 9 | 1 |
| Monocytes | 28058 | 35.1 | 7.1 | 10 | 26 |
| Eosinophils | 28017 | 35 | 5.7 | 11 | 18 |
| Basophils | 27996 | 35 | 3.4 | 12 | 28 |
| Albumin | 27551 | 34.4 | 9.8 | 13 | 3 |
| Urea | 27369 | 34.2 | 36.8 | 14 | 17 |
| Alkaline Phosphatase | 27080 | 33.9 | 8 | 15 | 21 |
| Total Protein | 26966 | 33.7 | 13.8 | 16 | 7 |
| Bilirubin (Total) | 26951 | 33.7 | 9.6 | 17 | 12 |
| Globulin | 26926 | 33.7 | 24.6 | 18 | 5 |
| Gamma-glutamyl Transferase | 26522 | 33.2 | 20.3 | 19 | 10 |
| Potassium | 25809 | 32.3 | 7.7 | 20 | 23 |
| C-reactive Protein | 24872 | 31.1 | 49.5 | 21 | 24 |
| Aspartate Transaminase | 24042 | 30.1 | 10.6 | 22 | 27 |
| APTT Ratio | 14319 | 17.9 | 20.2 | 23 | 16 |
| Corrected Calcium | 7572 | 9.5 | 22.5 | 24 | 22 |
| Magnesium | 7377 | 9.2 | 21.8 | 25 | 15 |
| Phosphate | 6943 | 8.7 | 26.1 | 26 | 25 |
| Troponin I | 5788 | 7.2 | 26 | 27 | 6 |
| Amylase | 4392 | 5.5 | 16.8 | 28 | 11 |
| D-Dimer | 2433 | 3 | 39.4 | 29 | 29 |
| Thyroid Stimulating Hormone | 1618 | 2 | 12.7 | 30 | 30 |
| Free Thyroxine | 1614 | 2 | 4 | 31 | 31 |
| Creatine Kinase | 1256 | 1.6 | 59.6 | 32 | 32 |
| NRBC | 1087 | 1.4 | 95.5 | 33 | 33 |
| Troponin T | 852 | 1.1 | 26.4 | 34 | 34 |
| Clauss Fibrinogen | 668 | 0.8 | 22.3 | 35 | 35 |
| Blood Lactate | 619 | 0.8 | 38 | 36 | 36 |
| Ferritin | 563 | 0.7 | 48 | 37 | 37 |
| Erythrocyte Sedimentation Rate | 563 | 0.7 | 66.6 | 38 | 38 |
| B12 | 534 | 0.7 | 12.7 | 39 | 39 |
| Folate | 504 | 0.6 | 32.1 | 40 | 40 |
| Venous pH | 483 | 0.6 | 79.5 | 41 | 41 |
| Venous Bicarbonate | 483 | 0.6 | 87.8 | 42 | 42 |
| Venous PCO2 | 483 | 0.6 | 86.7 | 43 | 43 |
| H+ (venous) | 483 | 0.6 | 48.4 | 44 | 44 |
| Venous PO2 | 482 | 0.6 | 89.8 | 45 | 45 |
| Salicylate | 473 | 0.6 | 0 | 46 | 46 |
| Cholesterol | 416 | 0.5 | 33.4 | 47 | 47 |
| Triglyceride | 406 | 0.5 | 16 | 48 | 48 |
| INR (warfarin) | 403 | 0.5 | 39 | 49 | 49 |
| HBA1c (DCCT) | 378 | 0.5 | 63.2 | 50 | 50 |

**Supplementary Table F. Descriptive statistics and feature importance for the top 50 tests (excluding vitals) derived from KCH.**

**Supplementary Figure R. Relationship between test frequency and abnormality rate in emergency department patients*.***

*Scatter plot showing the proportion of abnormal results versus total test frequency (log scale) for blood tests from emergency department attendances in the MIMIC-IV-ED cohort. Each point represents a unique laboratory test, colored by abnormality rate band (grey: no abnormal flags; dark purple to green: increasing proportions of abnormal results). Vertical dashed lines indicate frequency cutoffs for the top 30 and top 70 most commonly ordered tests. Commonly ordered tests (right side) demonstrate a range of abnormality rates, typically 10-50%. Notably, very high abnormality rates (>70%) are observed exclusively among infrequently ordered tests (left side), such as CD4/CD8 ratio (76% abnormal), Free Kappa (75%), and NTproBNP (70%). These high-abnormality, low-frequency tests represent investigations ordered only when clinicians have high pre-test probability of a specific condition, resulting in substantial enrichment for abnormal results compared to the baseline prevalence in the general emergency department population.*

| Frailty index at baseline | HR 1 year mortality (95% CI) | HR 10-year mortality (95% CI) |
| --- | --- | --- |
| 0 to 0.1 | 1.67 (1.53, 1.83) | 1.30 (1.27, 1.32) |
| >0.1 to 0.2 | 1.82 (1.72, 1.92) | 1.39 (1.37, 1.41) |
| >0.2 to 0.3 | 1.85 (1.71, 1.99) | 1.41 (1.38, 1.44) |
| >0.3 to 0.4 | 1.78 (1.52, 2.08) | 1.37 (1.31, 1.43) |
| >0.4 | 2.36 (1.33, 4.18) | 1.54 (1.34, 1.76) |

**Supplementary Table G: Hazard ratios (95% CI) for mortality per SD-increase in FI-lab within strata of the Williams et al frailty index at baseline.**

| Adapted frailty phenotype at baseline | HR 1 year mortality (95% CI) per SD increase in FI-lab | HR 10-year mortality (95% CI)  per SD increase in FI-lab |
| --- | --- | --- |
| Robust | 1.54 (1.41, 1.69) | 1.27 (1.25, 1.30) |
| Pre-frail | 1.73 (1.63, 1.84) | 1.39 (1.37, 1.41) |
| Frail | 1.81 (1.68, 1.94) | 1.40 (1.37, 1.44) |

**Supplementary Table H: Hazard ratios (95% CI) for mortality per SD-increase in FI-lab across baseline levels of the adapted Frailty Phenotype.**

| Level at baseline | FI-lab: HR 10 year mortality (95% CI) | Williams et al. frailty index: HR 10-year mortality (95% CI) |
| --- | --- | --- |
| 0 to 0.1 | Ref | Ref |
| >0.1 to 0.2 | 1.21 (1.17, 1.25) | 1.47 (1.43 ,1.52) |
| >0.2 to 0.3 | 1.78 (1.72, 1.84) | 2.30 (2.22, 2.39) |
| >0.3 to 0.4 | 3.02 (2.84, 3.20) | 3.46 (3.23, 3.71) |
| >0.4 | 5.22 (4.87, 5.59) | 3.86 (3.08, 4.84) |

**Supplementary Table I: Hazard ratios (95% CI) for 10-year mortality at specified levels of FI-lab and Williams et al. frailty index in UK Biobank – both variables in same model adjusted for age and sex.**

# **Supplementary Methods**

# **Data sources and cohort**

**Healthcare Setting Descriptions**

MIMIC-IV-ED (Beth Israel Deaconess Medical Center, Boston)

Beth Israel Deaconess Medical Center is a 673-bed academic tertiary care center in Boston, Massachusetts, serving as a major teaching hospital for Harvard Medical School. The emergency department (ED) sees approximately 60,000 visits annually, with a case mix ranging from minor injuries to complex medical emergencies. The hospital serves a diverse urban and suburban population.

King's College Hospital (London)

King's College Hospital NHS Foundation Trust (KCH) is a major tertiary referral center serving South London. This analysis used data from the Denmark Hill site only (not Princess Royal University Hospital). The Denmark Hill ED is one of the busiest in England with approximately 122,000 attendances annually. The hospital provides major trauma and specialist tertiary services. The catchment area serves a diverse, predominantly urban population including some of the most socioeconomically deprived areas in London.

UK Biobank

UK Biobank recruited 502,533 participants aged 37-73 years from 22 assessment centers across England, Scotland, and Wales between 2006-2010. Participants attended baseline assessment appointments at which blood samples were collected and processed by centralized laboratories. Hospital admission data were obtained through linkage to NHS Digital records.

**Data Access and Permissions**

MIMIC-IV-ED: We accessed complete de-identified extracts of the MIMIC-IV-ED database (version 2.2) including the following data tables: edstays.csv, vitalsign.csv, triage.csv, medrecon.csv, and linked core MIMIC-IV tables (patients.csv, admissions.csv, labevents.csv, d_labitems.csv). All dates were shifted for privacy protection. Access was granted through PhysioNet credentialing following completion of "Data or Specimens Only Research" training (CITI Program).

King's College Hospital: We accessed restricted extracts of electronic health record data approved under KCH Electronic Patient Record Interface committee approval 20230411B using the cogstack system. Data included emergency department attendance records, linked laboratory results, vital signs, and hospital admission/discharge records for the period 1 January 2017 to 1 January 2020.

UK Biobank: Analysis conducted under application 14151 with access to baseline assessment data (blood sample results, questionnaires) and linked hospital episode statistics through NHS Digital.

**Cohort and inclusion/exclusion criteria**
 We included adults (≥18 years) attending the emergency departments at Beth Israel Deaconess Medical Center (MIMIC-IV-ED, 2011–2019) and King’s College Hospital (KCH, 2017–2020), and adults enrolled in UK Biobank (baseline 2006–2010). For each ED attendance, we extracted demographics, vital signs, laboratory results, dispositions, admissions and deaths. ED stays were retained if at least one ED data element (vital signs, triage record or medication reconciliation) was present to permit NEWS2 derivation and time alignment; in MIMIC-IV-ED this criterion did not exclude any stays. Laboratory results were linked to ED arrival using subject identifiers and a 0–24 hour window post-arrival. For KCH, we excluded records with non-binary gender (n < 100), deaths recorded before ED arrival, and implausible hospital stays (negative duration or >1,000 days). Mortality analyses used each individual’s first eligible ED attendance; all other outcomes used all eligible attendances. For readmission outcomes, patients who died during the index admission were excluded and follow-up was censored at death. Length-of-stay analyses excluded deaths and zero-day stays. The cohort flow is shown in Figure S1. Per-criterion exclusion counts for KCH were as follows: age <18 or missing/implausible, n = 101,871 (predominantly paediatric); records with unparseable datetimes, n = 12,868; admitted before the study window (≤31/12/2016), n = 150; non-binary or missing gender, n = 90; death recorded before ED arrival, n = 76; zero or negative length of stay, n = 51; length of stay >1,000 days, n = 43. Criteria overlap and do not sum exactly to the total of 115,048 records excluded by at least one criterion. Of the 182,052 records subsequently excluded at lab-linkage, 10,622 were admitted to hospital, 52 died in the ED, 268 died in hospital, and the remainder were discharged from the ED. For MIMIC-IV-ED the only exclusion was lab linkage (425,087 → 227,736); for UK Biobank, blood test availability (502,533 → 492,703).

**Supplementary: cohort selection algorithm**

1. Adults: 18 ≤ age_at_ed ≤ 120 (age = anchor_age + year(intime) − anchor_year).
2. Labs linked to ED: retain labs with 0 ≤ (charttime − intime) ≤ 24 h.
3. Required ED metadata present (vitals/triage/meds) to compute NEWS2 and align times.
4. KCH exclusions: non-binary gender; deathtime < intime; LOS < 0 or > 1,000 days.
5. Analysis sets: mortality = first ED stay per person; others = all eligible stays.
6. Outcome-specific: readmission - exclude in-hospital deaths; censor at death. LOS - exclude deaths or LOS ≤ 0.

**Laboratory Data Processing and Linkage**

**Data Extraction**

Laboratory results were extracted from source CSV files.

**Linkage Algorithm**

Laboratory measurements were linked to emergency department encounters using the following deterministic algorithm:

1. Match on patient identifier (subject_id field) between labevents and cohort tables

2. Calculate time difference: hours_from_ed = (charttime - intime) / 3600 seconds

3. Retain measurements where 0 ≤ hours_from_ed ≤ 24

**Linkage Quality**

In MIMIC-IV-ED, 45.2 % of eligible ED stays had ≥1 successfully linked laboratory test. In KCH, 36.2% had successful linkage. The majority of patients without linkage had attended with minor injuries or other issues which did not require laboratory investigation.

**Multiple Measurements**

When multiple measurements of the same test (itemid) existed within the 24-hour window, we retained the first measurement (minimum hours_from_ed value) to reflect initial ED assessment rather than follow-up or serial monitoring. For ED encounters, duplicate visit records were handled by grouping by visit identifier and retaining unique visits.

**Laboratory Reference Ranges**

MIMIC-IV-ED: Binary abnormality flags provided in the 'flag' field of labevents.csv (values: "normal", "abnormal", or missing). These reflect laboratory-specific reference ranges applied by Beth Israel Deaconess Medical Center clinical laboratories.

KCH: Reference ranges provided separately by the clinical laboratory. Tests were flagged as abnormal if measured value fell outside the provided lower/upper reference limits.

UK Biobank: Results compared against reference ranges provided by the UK Biobank coordinating laboratory.

Tests without available reference ranges were excluded from FI-Lab calculation

## **FI-Lab construction**

FI-Lab was defined as the proportion of abnormal results among tests actually available for a patient (no imputation). Abnormality used site-specific reference ranges (MIMIC laboratory flags; KCH provided ranges). Tests without a reference range were excluded.

- **Window:** results within 24 hours of ED arrival.
- **Multiplicity:** where multiple measurements existed, the first value was used.
- **Linkage:** laboratory results were linked to ED stays by subject identifier and timestamp, retaining measurements 0–24 hours post-arrival.
- **Vital signs as tests (sensitivity):** first recorded vital signs were converted to normal/abnormal using clinical ranges (e.g., respiratory rate <12 or >20 breaths/min; SpO₂ <95%; temperature <36.1 or >38.0 °C; systolic BP <90 or >140 mmHg; heart rate <60 or >100 bpm; diastolic BP <60 or >90 mmHg) and treated as additional “tests”.
- **Feature floor:** configurations required a minimum number of available tests; if not met, the FI-Lab score was set to missing.

## **Configuration search**

We varied three elements to identify a performant and robust construction:

1. **Test-selection strategy**

- **Common:** tests ranked by frequency of ordering (availability).
- **Important:** tests ranked by prognostic importance for 1-year mortality using a random-forest classifier (ranger; permutation importance) trained on eligible labs (conducted in ≥5% of patients) with inverse-prevalence case weights; importance was availability-weighted. Where random-forest importance could not be estimated (e.g., insufficient data), we used a fallback based on abnormality rate weighted by test frequency/availability.
- **Random:** at each list size, 20 random sets sampled from eligible tests.

2. **Number of tests:** 10, 15, 20, 25, 30, 35, 40, 50, 60, 70.
3. **Feature floor (minimum tests required):** 0, 5, 10, 15, 20.

For each configuration and stay we recorded the number of tests used, number abnormal, and FI-Lab = abnormal/used.

## **NEWS2 calculation**

NEWS2 was calculated from first vital signs using standard scoring for seven parameters (respiratory rate, oxygen saturation, supplemental oxygen, temperature, systolic blood pressure, heart rate, consciousness). Temperatures >50 were assumed Fahrenheit and converted to Celsius. Supplemental oxygen use was identified from medication records or inferred from oxygen flow. Consciousness was inferred from triage descriptors; where not recorded, ‘Alert’ was assumed. If >2 components were missing, NEWS2 was set to missing.

## **Outcomes and time origin**

- **1-year mortality:** time from ED arrival to death; censored at 365.25 days.
- **In-hospital death and length of stay (LOS):** among admissions; LOS modelled as time to discharge with in-hospital death treated as censoring.
- **Readmission:** among admissions, time from discharge to next admission; death treated as censoring (30-day and 1-year).
- **ED admission (binary):** disposition at the index ED visit.

**Outcome Definitions and Ascertainment**

**Mortality:**

- *MIMIC-IV-ED:* Date of death was obtained from the dod column in the patients table, derived from hospital records and Massachusetts state death records. State records were matched to patients using a deterministic linkage algorithm based on last name, date of birth, and social security number. Where both hospital and state records existed, hospital records took precedence. Deaths occurring more than one year after the last hospital discharge were censored as part of the de-identification process.
- *King's College Hospital:* Date of death was obtained from hospital administrative records, linked to NHS Digital spine records via NHS number. Deaths occurring outside of KCH but within the NHS system were captured through this linkage.
- *UK Biobank:* Mortality data obtained through linkage to national death registries: NHS England (for deaths in England and Wales) and NHS Central Register, National Records of Scotland (for deaths in Scotland). All UK Biobank participants were flagged with these data providers from the date of recruitment, and death data extracts are received quarterly. Date of death and ICD-10 coded causes of death are provided.

**Hospital Readmissions:** Readmission events were identified only within the same hospital system (MIMIC-IV: Beth Israel Deaconess Medical Center; KCH: King's College Hospital NHS Foundation Trust). Readmissions to other healthcare facilities were not captured.

**ED Admission:** ED admission (disposition from ED to hospital) was determined from the discharge destination field in ED records, indicating whether patients were admitted to the hospital versus

## **Statistical analysis**

For each outcome and FI-Lab configuration we fitted Cox proportional-hazards models comparing:

1. Age + sex;
2. Age + sex + NEWS2;
3. Age + sex + FI-Lab;
4. Age + sex + FI-Lab + NEWS2.

Continuous predictors were standardised; hazard ratios are per SD increase with 95% CIs. Mortality models used the first ED stay per patient; LOS, readmission and ED-admission models used all eligible stays with outcome-specific exclusions as above. ED admission was analysed as a binary event using a Cox model with constant time (equivalent to a risk-ratio model). Models used complete-case analysis for the predictors in each specification; combined models were restricted to stays with non-missing NEWS2. We intentionally applied minimal adjustment to estimate FI-Lab’s independent prognostic value. Proportional-hazards assumptions were not formally tested, and no multiplicity correction was applied given the exploratory configuration search. Performance was summarised with Harrell’s C-statistic and AIC.

Following primary configuration analyses, we selected 25 commonly ordered tests with a feature floor of 15 as the optimal FI-Lab configuration for all sensitivity analyses.

**Age stratification**

To assess performance across the age spectrum, we fitted separate models within 10-year age bands (<30, 30-40, 40-50, 50-60, 60-70, 70-80, ≥80 years) for one-year mortality. Within each age band, models adjusted for age (continuous, within band), gender, and NEWS2 where available. UK Biobank analyses were limited to age bands 40-49, 50-59, 60-69, and 70+ due to the recruitment age range (37-73 years at baseline).

**Race, ethnicity, and socioeconomic status**

Race and ethnicity data were standardized within datasets but not harmonized across datasets due to different national classification systems. In MIMIC-IV-ED, race categories were collapsed as: Asian (any Asian ethnicity); Black/African American (Black, African, or African American); Hispanic/Latino (Hispanic or Latino); White (White or Caucasian); Other (all other reported ethnicities); and Unknown/declined. In KCH and UK Biobank, ethnicity was recorded using UK Office for National Statistics 2011 Census categories: White; Black or Black British; Asian or Asian British; Chinese; Mixed; Other ethnic group; and Prefer not to answer (KCH only).

Socioeconomic status was measured using insurance status in MIMIC-IV-ED (categorized as: Medicare; Medicaid; Private; Self-pay; Other/Unknown), Index of Multiple Deprivation (IMD) in KCH, and Townsend deprivation index in UK Biobank.

For one-year mortality, we fitted Cox models adjusted for race/ethnicity and socioeconomic status, including FI-Lab, age, gender, race/ethnicity, and the dataset-specific socioeconomic measure as covariates, using White ethnicity and Private insurance (MIMIC) as reference categories. We tested for effect modification by race/ethnicity using likelihood ratio tests comparing models with and without FI-Lab × race/ethnicity interaction terms. We also fitted separate models within each racial/ethnic group where sample size permitted (≥100 patients and ≥20 events), adjusting for age, gender, and socioeconomic status within each stratum.

**Inclusion of vital signs as binary features**

We compared FI-Lab performance with and without vital signs included as binary abnormal/normal features. Vital signs (respiratory rate, oxygen saturation, temperature, systolic and diastolic blood pressure, heart rate) were converted to binary using clinical reference ranges: respiratory rate <12 or >20 breaths/min; SpO₂ <95%; temperature <36.1 or >38.0°C; systolic BP <90 or >140 mmHg; heart rate <60 or >100 bpm; diastolic BP <60 or >90 mmHg. These thresholds differ from NEWS2 scoring to reflect general clinical abnormality rather than acuity weighting. Vital sign measurements were treated as additional features in the FI-Lab calculation, with abnormality determined by the specified ranges.

**Uncertainty and robustness**

- **Sampling variability of test lists:** for each list size, FI-Lab was computed using 20 random test sets; within-patient variation (mean, SD, coefficient of variation, range) was summarised by list size and feature floor.
- **Measurement noise (KCH subset):** To assess FI-Lab stability against laboratory measurement uncertainty, we randomly sampled 5,000 patients from the KCH database. For each patient, the FI-Lab score was recalculated 20 times with noise introduced to laboratory values prior to each calculation. Noise was generated using test-specific analytical variation based on manufacturer specifications and clinical laboratory standards: tests with proportional error (e.g., hemoglobin, creatinine) used coefficient of variation (CV) ranging from 2-10% depending on the analyte; tests with constant absolute error (e.g., sodium ±1.0 mmol/L, potassium ±0.1 mmol/L, corrected calcium ±0.05 mmol/L) used fixed standard deviations. For each iteration, noise was randomly sampled from a normal distribution with the test-specific standard deviation, added to the measured value, and abnormality was reassessed using the original reference ranges. This simulated how near-threshold measurements might variably classify as normal or abnormal due to analytical imprecision.

## **Coverage reporting**

For each configuration we report the number with calculable FI-Lab, median features used, the proportion achieving the full requested set, and the distribution of FI-Lab values.

## **Software and reproducibility**

Analyses used R (v4.5.0 for MIMIC; v4.2.1 for KCH) with tidyverse, data.table, lubridate, survival, ranger, DBI and duckdb. Random seeds were set where applicable. ED-windowed laboratory extraction used DuckDB SQL against flat files. Summary tables and model outputs were exported to CSV.

## **Approvals and data access**

MIMIC-IV-ED is publicly available after credentialling; KCH analyses were approved by the King’s Electronic Patient Record Interface committee (20230411B); UK Biobank analyses were conducted under application 14151. All analyses used de-identified data.

# **Supplementary Limitations**

## **Design, setting, and generalisability**

- **Retrospective design:** establishes associations, not causation.
- **Health-system context:** cohorts from high-income settings; findings may not generalise to other jurisdictions or resource-constrained environments.
- **Data requirements:** reliance on EHRs with coded laboratory data limits applicability where such infrastructure is absent or incomplete.
- **Cohort composition:** adults (≥18 years); performance in younger populations and specific subgroups may differ despite age-stratified checks.

## **Cohort selection and linkage**

- **Inclusion dependency on ED records:** inclusion required at least one supplementary ED record (vital signs, triage, or medication reconciliation). Incomplete ED documentation in non-MIMIC datasets could lead to exclusion and selection bias.
- **Laboratory linkage window:** labs were linked by subject identifier and timestamp within 0–24 hours post-arrival. Tests performed before ED arrival (e.g., in the community or during ambulance transfer) were not captured, likely underestimating laboratory coverage and potentially biasing denominators.
- **First-measurement rule:** where multiple values existed in the window, the first was used. Timing heterogeneity (e.g., pre- vs post-intervention) may introduce bias.

## **Measurement and variable definition**

- **Reference ranges:** abnormality used site-specific reference ranges (MIMIC flags; KCH provided ranges). Differences in analytical platforms, calibration, or population-specific ranges (including age-/sex-specific normals) may yield non-comparable “abnormal” designations across sites.
- **Dichotomisation at cut-points:** small analytical or biological variation near thresholds can flip classification (e.g., sodium 134.9 vs 135.1 mmol/L). Our measurement-noise assessment was conducted in a subset only.
- **Vital signs handling:** for NEWS2, temperatures >50 were assumed Fahrenheit and converted; erroneous Celsius entries above 50 would be misclassified. Supplemental oxygen was inferred from medication/flow records, which may be incomplete. Consciousness was inferred from triage descriptors and defaulted to “Alert” if unspecified—susceptible to misclassification.
- **Vital signs as “tests” (sensitivity):** converting vital signs to binary abnormal/normal uses simple clinical ranges rather than NEWS2 thresholds and may not reflect nuanced clinical states.

## **FI-Lab construction choices**

- **Denominator variability:** FI-Lab adjusts to tests actually available; test ordering is influenced by acuity and workflow. Even with denominator adjustment and feature floors, selection effects may persist (sicker patients often undergo broader testing).
- **Feature floors:** requiring a minimum number of available tests (0–20) improves comparability but excludes patients with sparse testing, potentially introducing selection bias.
- **Test-set strategies:** “Important” lists used random-forest permutation importance on tests present in ≥5% of patients, availability-weighted. This favours commonly available tests and may down-weight rare but clinically salient markers.
- **Subsampling for importance:** the random-forest model was trained on a random subsample (up to 200,000 individuals) to control runtime; estimated importances may vary with the sampling frame. Where RF importance could not be estimated, we used a fallback based on abnormality rate × availability.
- **Random sets and stability:** sampling variability was assessed using 20 random sets per list size; while informative, this does not exhaust the combinatorial space.

## **Outcomes and ascertainment**

- **Mortality capture:** completeness of out-of-hospital deaths may vary by dataset; under-ascertainment would bias mortality estimates.
- **Readmissions:** identified within the same data systems; out-of-network readmissions may be missed, underestimating incidence.
- **Length of stay (LOS):** modelled as time to discharge; in-hospital death treated as censoring, which may bias LOS estimates in the sickest patients.
- **Time origins:** ED arrival for mortality/admission vs hospital discharge for readmission; differences in time-zero introduce differing at-risk populations.

## **Statistical modelling**

- **Limited adjustment:** models deliberately adjusted only for age, sex, and NEWS2 to isolate FI-Lab’s incremental value; residual confounding (comorbidities, diagnosis, treatments, socioeconomic factors) is likely.
- **Multiple comparisons:** many FI-Lab configurations were tested without adjustment for multiplicity; chance findings are possible.
- **Proportional-hazards assumption:** not formally tested; violations could bias hazard-ratio estimates.
- **Model choices:** for readmission and length of stay we used standard Cox models rather than competing-risk methods (computational constraints), potentially underestimating incidence in the presence of the competing risk of death.
- **Complete-case analysis:** each model used complete cases for included predictors; combined models required non-missing NEWS2, which may induce selection bias via informative missingness.
- **Repeated measures:** for non-mortality outcomes we used multiple ED stays per person but did not model within-person correlation (no clustering or robust variance), risking underestimated standard errors.
- **Functional form and interactions:** predictors were standardised and entered linearly; non-linear effects and interactions (e.g., FI-Lab × age) were not explored.
- **Performance metrics:** discrimination and information criteria were reported (C-index, AIC); we did not present calibration, reclassification, decision-curve analysis, or net benefit.

## **External validity and transportability**

- **Across sites and time:** practice patterns, ordering behaviour, and assay characteristics vary by site and epoch; FI-Lab lists derived in one context may not transport without re-specification.
- **Operational dependence:** real-world performance depends on timely lab availability and stable reference ranges; drift in lab systems could affect score behaviour.

## **Clinical impact**

- **Utility not tested:** we did not evaluate whether presenting FI-Lab to clinicians changes decisions or improves outcomes; this requires prospective, ideally randomised, evaluation with implementation considerations (alert fatigue, workflow integration).

## **Practical and computational constraints**

- **Runtime controls:** subsampling for random-forest importance was used for computational efficiency, and may limit reproducibility of exact variable rankings across runs/datasets.
- **Code paths:** NEWS2 derivation relied on heuristics for oxygen and consciousness when explicit fields were absent; errors in upstream documentation propagate to derived scores.
- **Model choices:** for readmission and length of stay we used standard Cox models rather than competing-risk methods (computational constraints), potentially underestimating incidence in the presence of the competing risk of death.
