# Supplementary material for: Evaluation of the FI-Lab, a laboratory-based, automated frailty index for acute care: A multicohort study
Source: PLoS Med. 2026 Sep 1;23(9):e1005004. doi: 10.1371/journal.pmed.1005004 (PMC13577487; doi:10.1371/journal.pmed.1005004)
Supplement: Supplementary file 2 [file pmed.1005004.s002.docx]

**STROBE-RECORD Checklist**

Evaluation of the FI-Lab, a laboratory-based, automated frailty index for acute care: a multicohort study

| **Section/Topic** | **Item No.** | **STROBE Items** | **Location in Manuscript** | **RECORD Items** | **Location in Manuscript** |
| --- | --- | --- | --- | --- | --- |
| **Title and Abstract** | **1** | (a) Indicate the study's design with a commonly used term in the title or the abstract  (b) Provide in the abstract an informative and balanced summary of what was done and what was found | *Abstract* | RECORD 1.1: The type of data used should be specified in the title or abstract. When possible, the name of the databases used should be included. | *Abstract, Methods section* |
|  |  |  |  | RECORD 1.2: If applicable, the geographic region and timeframe within which the study took place should be reported in the title or abstract. | *Abstract, Methods section* |
|  |  |  |  | RECORD 1.3: If linkage between databases was conducted for the study, this should be clearly stated in the title or abstract. | *Not in abstract (word limit); fully described in Methods* |
| **Introduction** | **2** | Explain the scientific background and rationale for the investigation being reported | *Introduction (paragraphs 1-3)* |  |  |
|  | **3** | State specific objectives, including any prespecified hypotheses | *Introduction (final paragraph: 'To realize the potential...')* |  |  |
| **Methods** | **4** | Present key elements of study design early in the paper | *Methods (Study Design and Data Sources; first paragraph)* |  |  |
|  | **5** | Describe the setting, locations, and relevant dates, including periods of recruitment, exposure, follow-up, and data collection | *Methods (Study Design and Data Sources); Supplementary Methods (Healthcare Setting Descriptions)* |  |  |
|  | **6** | (a) Give the eligibility criteria, and the sources and methods of selection of participants. Describe methods of follow-up  (b) For matched studies, give matching criteria and number of exposed and unexposed | *Methods (Study Population, Exclusions); Supplementary Methods (Cohort and inclusion/exclusion criteria)* | RECORD 6.1: The methods of study population selection (such as codes or algorithms used to identify subjects) should be listed in detail. | *Supplementary Methods (Cohort selection algorithm)* |
|  |  |  |  | RECORD 6.2: Any validation studies of the codes or algorithms used to select the population should be referenced. | *Reference 18 (Sapp et al. meta-analysis)* |
|  |  |  |  | RECORD 6.3: If the study involved linkage of databases, consider use of a flow diagram or other graphical display to demonstrate the data linkage process. | *Supplementary Figure S1* |
|  | **7** | Clearly define all outcomes, exposures, predictors, potential confounders, and effect modifiers. Give diagnostic criteria, if applicable. | *Methods (Laboratory-Based Frailty Index Construction, Primary and Secondary Outcomes); Supplementary Methods (FI-Lab construction, Outcome Definitions and Ascertainment)* | RECORD 7.1: A complete list of codes and algorithms used to classify exposures, outcomes, confounders, and effect modifiers should be provided. | *Supplementary Methods (Outcome Definitions and Ascertainment, Laboratory Reference Ranges); Supplementary Tables 4-7* |
|  | **8** | For each variable of interest, give sources of data and details of methods of assessment (measurement). Describe comparability of assessment methods if there is more than one group | *Methods (Laboratory-Based Frailty Index Construction); Supplementary Methods (Laboratory Data Processing and Linkage, Laboratory Reference Ranges)* |  |  |
|  | **9** | Describe any efforts to address potential sources of bias | *Methods (Statistical Analysis); Discussion (Limitations); Supplementary Limitations* |  |  |
|  | **10** | Explain how the study size was arrived at | *Methods (Study Population) - observational study using all available eligible data from existing databases* |  |  |
|  | **11** | Explain how quantitative variables were handled in the analyses. If applicable, describe which groupings were chosen, and why | *Methods (Statistical Analysis); Supplementary Methods (Statistical analysis, Age stratification)* |  |  |
|  | **12** | (a) Describe all statistical methods, including those used to control for confounding  (b) Describe any methods used to examine subgroups and interactions  (c) Explain how missing data were addressed  (d) If applicable, explain how loss to follow-up was addressed  (e) Describe any sensitivity analyses | *Methods (Statistical Analysis); Supplementary Methods (Statistical analysis, Age stratification, Race/ethnicity/SES, Inclusion of vital signs, Uncertainty and robustness)* | RECORD 12.1: Authors should describe the extent to which the investigators had access to the database population used to create the study population. | *Supplementary Methods (Data Access and Permissions)* |
|  |  |  |  | RECORD 12.2: Authors should provide information on the data cleaning methods used in the study. | *Supplementary Methods (Laboratory Data Processing and Linkage - Multiple Measurements, Linkage Quality; Cohort and inclusion/exclusion criteria; NEWS2 calculation)* |
|  |  |  |  | RECORD 12.3: State whether the study included person-level, institutional-level, or other data linkage across two or more databases. The methods of linkage and methods of linkage quality evaluation should be provided. | *Supplementary Methods (Laboratory Data Processing and Linkage - Linkage Algorithm, Linkage Quality)* |
| **Results** | **13** | (a) Report the numbers of individuals at each stage of the study  (b) Give reasons for non-participation at each stage  (c) Consider use of a flow diagram | *Results (Study Population); Table 1; Supplementary Figure S1* | RECORD 13.1: Describe in detail the selection of the persons included in the study (i.e., study population selection) including filtering based on data quality, data availability and linkage. | *Results (Study Population); Supplementary Figure S1; Supplementary Methods (Cohort and inclusion/exclusion criteria, Laboratory Data Processing - Linkage Quality)* |
|  | **14** | (a) Give characteristics of study participants  (b) Indicate the number of participants with missing data for each variable of interest  (c) Cohort study - summarise follow-up time | *Results (Study Population); Table 1* |  |  |
|  | **15** | Report numbers of outcome events or summary measures over time | *Results (Study Population); Table 1; Results (FI-Lab Performance Across Configurations)* |  |  |
|  | **16** | (a) Give unadjusted estimates and, if applicable, confounder-adjusted estimates and their precision. Make clear which confounders were adjusted for and why they were included  (b) Report category boundaries when continuous variables were categorized  (c) If relevant, consider translating estimates of relative risk into absolute risk | *Results (Performance Compared to Established Measures and Between Sexes, Secondary Outcomes); Figure 2; Supplementary Tables 2-3* |  |  |
|  | **17** | Report other analyses done—e.g., analyses of subgroups and interactions, and sensitivity analyses | *Results (Sensitivity Analyses); Supplementary Tables 2-3 and 8-10; Supplementary Figures S2-S18* |  |  |
| **Discussion** | **18** | Summarise key results with reference to study objectives | *Discussion (paragraphs 1-2: 'In this large, multicohort study...')* |  |  |
|  | **19** | Discuss limitations of the study, taking into account sources of potential bias or imprecision. Discuss both direction and magnitude of any potential bias | *Discussion (Limitations); Supplementary Limitations (comprehensive section covering all aspects)* | RECORD 19.1: Discuss the implications of using data that were not created or collected to answer the specific research question(s). Include discussion of misclassification bias, unmeasured confounding, missing data, and changing eligibility over time. | *Discussion (Limitations - paragraph 1); Supplementary Limitations (Measurement and variable definition, Outcomes and ascertainment, Statistical modelling sections)* |
|  | **20** | Give a cautious overall interpretation of results considering objectives, limitations, multiplicity of analyses, results from similar studies, and other relevant evidence | *Discussion (Key Findings, Practical Implementation Guide, Limitations, Conclusion)* |  |  |
|  | **21** | Discuss the generalisability (external validity) of the study results | *Discussion (Limitations - paragraph 1: 'Data from two high-income countries...'); Supplementary Limitations (Design, setting, and generalisability; External validity and transportability)* |  |  |
| **Other Information** | **22** | Give the source of funding and the role of the funders for the present study and, if applicable, for the original study on which the present article is based | *Sources of funding* | RECORD 22.1: Authors should provide information on how to access any supplemental information such as the study protocol, raw data, or programming code. | *Supplementary Methods (Code availability): GitHub repository* [*https://github.com/jhfl2/FI-Lab-Recipe*](https://github.com/jhfl2/FI-Lab-Recipe) *or https://doi.org/10.5281/zenodo.21686996* |

*STROBE*: von Elm E, Altman DG, Egger M, Pocock SJ, Gøtzsche PC, Vandenbroucke JP, et al. (2007). The Strengthening the Reporting of Observational Studies in Epidemiology (STROBE) Statement: Guidelines for Reporting Observational Studies. PLoS Med. 4(10): e296. <https://doi.org/10.1371/journal.pmed.0040296>

*RECORD*: Benchimol EI, Smeeth L, Guttmann A, Harron K, Moher D, Petersen I, Sørensen HT, von Elm E, Langan SM; RECORD Working Committee. (2015). The REporting of studies Conducted using Observational Routinely-collected health Data (RECORD) statement. PLoS Med. 12(10):e1001885. doi: 10.1371/journal.pmed.1001885
