# Supplementary material for: Evaluation of the FI-Lab, a laboratory-based, automated frailty index for acute care: A multicohort study
Source: PLoS Med. 2026 Sep 1;23(9):e1005004. doi: 10.1371/journal.pmed.1005004 (PMC13577487; doi:10.1371/journal.pmed.1005004)
Supplement: Supplementary file 5 [file pmed.1005004.s005.docx]

**Response to Reviewers**

**Editorial Requests**

| **Comment** | **Response** |
| --- | --- |
| In revising the manuscript for further consideration here, please ensure you address the specific points made by each reviewer and the editors. In your rebuttal letter you should indicate your response to the reviewers' and editors' comments and the changes you have made in the manuscript. Please submit a clean version of the paper as the main article file. A version with changes marked must also be uploaded as a marked up manuscript file. | We will ensure that we upload this Response to Reviewer letter, a tracked changes version of the manuscript, and a clean version of the manuscript. |
| We ask every co-author listed on the manuscript to fill in a contributing author statement. | N/A (co-authors will be contacted by the journal to approve the submission, its content, and authorship).  We have also provided a CRediT taxonomy of Author Contributions in the manuscript. |
| Please review your reference list to ensure that it is complete and correct. | Done. |
| GENERAL EDITORIAL REQUESTS * Please confirm that your title complies with PLOS Medicine's style. Your title must be nondeclarative and not a question. It should begin with main concept if possible. "Effect of" should be used only if causality can be inferred, i.e., for an RCT. Please place the study design ("A randomized controlled trial," "A retrospective study," "A modelling study," etc.) in the subtitle (ie, after a colon). e.g “Evaluation of FI-Lab, a laboratory-based, automated frailty index for acute care: a multicohort study” this is only an example and we are open for other suggestions. | Done: Changed the title to “Evaluation of the FI-Lab, a laboratory-based, automated frailty index for acute care: a multicohort study”. |
| * Please confirm that your abstract complies with our requirements, including format (three sections: Background, Methods and Findings, and Conclusions) and providing all the information relevant to this study type [https://journals.plos.org/plosmedicine/s/submission-guidelines#loc-abstract](https://can01.safelinks.protection.outlook.com/?url=https%3A%2F%2Fjournals.plos.org%2Fplosmedicine%2Fs%2Fsubmission-guidelines%23loc-abstract&data=05%7C02%7Calexandra.vandervalk%40nshealth.ca%7C4b505c79745f40a6201208dee32a4c42%7C8eb23313ce754345a56a297a2412b4db%7C0%7C0%7C639197967983719557%7CUnknown%7CTWFpbGZsb3d8eyJFbXB0eU1hcGkiOnRydWUsIlYiOiIwLjAuMDAwMCIsIlAiOiJXaW4zMiIsIkFOIjoiTWFpbCIsIldUIjoyfQ%3D%3D%7C0%7C%7C%7C&sdata=kAhZ2YGpvoiA0M6RzCjP8DFsp6KVnQE0EXXhqAJWQyI%3D&reserved=0) | Done. Please note that we have made additional minor edits to the Abstract as requested below. As a result, we have also done some minor whittling to preserve the word count (<300 words). |
| * Please ensure that the Introduction ends with a clear description of the study question or hypothesis. | Done. |
| * Please ensure that all abbreviations are defined at first use throughout the text. | Done. |
| * Please confirm that all numbers presented in the abstract are present and identical to numbers presented in the main manuscript text. | Done. We have fixed a small error in the Results section (paragraph 1), where the participant sample size for UK Biobank was incorrectly listed as 492,700 – we have fixed this to be 492,703 to match the existing (correct) instances in the Abstract, Manuscript, and Supplement.  We have also added to the abstract the Hazard Ratios for the UK Biobank cohort, as follows: (1.87 [95% CI, 1.80, 1.94]; p<0.001).  During this process, we also identified that Supplemental Figure S1 (STROBE flowchart) was missing the third panel for the UK Biobank sample. We have revised the figure to incorporate the UK Biobank cohort. |
| GENERAL * Please disclose any use of AI tools and technologies to the study or article content at the end of the Methods. Please specify the name(s) and version(s) of any tool(s) used and a description of their use, including how the AI-assisted or generated content was evaluated by authors, and a clear statement of which aspects of the study, article contents, data, or supporting files were affected/generated by AI tool usage. Please also include a statement specifying who retains responsibility for the final manuscript. See our guidelines for more information: [https://journals.plos.org/plosmedicine/s/ethical-publishing-practice#loc-artificial-intelligence-tools-and-technologies](https://can01.safelinks.protection.outlook.com/?url=https%3A%2F%2Fjournals.plos.org%2Fplosmedicine%2Fs%2Fethical-publishing-practice%23loc-artificial-intelligence-tools-and-technologies&data=05%7C02%7Calexandra.vandervalk%40nshealth.ca%7C4b505c79745f40a6201208dee32a4c42%7C8eb23313ce754345a56a297a2412b4db%7C0%7C0%7C639197967983729753%7CUnknown%7CTWFpbGZsb3d8eyJFbXB0eU1hcGkiOnRydWUsIlYiOiIwLjAuMDAwMCIsIlAiOiJXaW4zMiIsIkFOIjoiTWFpbCIsIldUIjoyfQ%3D%3D%7C0%7C%7C%7C&sdata=ZqS8CZT0dAJdvpHwC%2Bwu1URV7Rf4rPLU9lL0wzeF29w%3D&reserved=0). | As requested, we have revised our AI statement and moved it from the Acknowledgement section to the end of the Methods section, as follows:  “Claude Opus 4.0 (Anthropic) was used to refine code as necessary, primarily to improve efficiency, comprehensibility, and to streamline ggplot syntax used for visualization. The authors manually tested the code and relevant outputs post-iteratively to ensure accuracy.” |
| * Please review your text for claims of novelty or primacy (e.g. 'for the first time') and remove this language. In addition, please check that any use of statistical terms (such as trend or significant) are supported by the data, and if not please remove them. | Done. |
| * Please remove the 'conclusions' subheading from the discussion. Please also remove any other subheadings from the discussion. | Done. |
| * Statistical reporting: Please revise throughout the manuscript, including tables and figures.  - Please report statistical information as follows to improve clarity for the reader ""22% (95% CI [13,28]; p</=)"". | Done. |
| - Please separate upper and lower bounds with commas instead of hyphens as the latter can be confused with reporting of negative values. | Done (fixed in main text and supplement). |
| - Please repeat statistical definitions (HR, CI etc.) for each set of parentheses." | Done. |
| * In the last sentence of the Abstract Methods and Findings section, please describe the main limitation(s) of the study's methodology. | We have added a sentence regarding the study’s main limitation to the end of the Methods and Findings section of the Abstract. |
| * In the author summary, please revise formatting and ensure you use bullet points. | Done. |
| FUNDING STATEMENT * The funding statement should include: specific grant numbers, initials of authors who received each award, URLs to sponsors’ websites. | Done. Please note that the Dalhousie UIMRF and DRIVE-Health awards do not have associated grant numbers. |
| Also, please state whether any sponsors or funders (other than the named authors) played any role in study design, data collection and analysis, the decision to publish, or preparation of the manuscript. If they had no role in the research, include this sentence: “The funders had no role in study design, data collection and analysis, decision to publish, or preparation of the manuscript. | Done. |
| COMPETING INTERESTS STATEMENT * Please revise Competing Interest statement and remove first sentence – PhD’s student research is not a conflict. | Done. |
| DATA AVAILABILITY * PLOS defines the “minimal data set” to consist of the data set used to reach the conclusions drawn in the manuscript with related metadata and methods, and any additional data required to replicate the reported study findings in their entirety. Authors do not need to submit their entire data set, or the raw data collected during an investigation. Please submit the following data: The values behind the means, standard deviations and other measures reported; The values used to build graphs; The points extracted from images for analysis. | Thank you – we have uploaded a minimal data set to our GitHub repository, available at: <https://github.com/jhfl2/FI-Lab-Recipe> |
| * The Data Availability Statement (DAS) requires revision. Please include DAS in your manuscript. For each data source used in your study: a) If the data are freely or publicly available, note this and state the location of the data: within the paper, in Supporting Information files, or in a public repository (include the DOI or accession number) b) If the data are owned by a third party but freely available upon request, please note this and state the institutional owner of the data set and contact information for data requests (insitutional web or email address). Note that a study author cannot be the contact for the data. c) If the data are not freely available, please describe briefly the ethical, legal, or contractual restriction that prevents you from sharing it. Please also include an appropriate institutional contact (insitutional web or email address) for inquiries (again, this cannot be a study author). | Done. We have added the following Data Availability Statement:  “UK Biobank data are available for use subject to application and approval of a data sharing agreement (https://www.ukbiobank.ac.uk/use-our-data/apply-for-access/). MIMIC-IV-ED data are freely available upon request to the Massachusetts Institute of Technology (https://mimic.mit.edu/docs/faq/how-to-get-access.html). King’s College Hospital data are not publicly available; access is typically limited to approved researchers who meet the relevant data access and ethics requirements. All analysis code for FI-Lab is available at GitHub (https://github.com/jhfl2/FI-Lab-Recipe).” |
| * For studies in which a novel model is central to the manuscript's findings, as is the case here, authors are responsible for providing the source code needed to replicate the study's findings in a repository (such as GitHub, SourceForge or Bitbucket) or a cloud computing service (such as Code Ocean).  Please include a citable digital object identifier (DOI) or other persistent identifier of the code (such as through linkage with Zenodo). Protection of authors’ intellectual property will not be cause for exception. Please explain in the manuscript’s Data Availability Statement how readers can access the shared code.  * Please include the statement on code availability in the data availability statement. Please see guidance here:https://journals.plos.org/plosmedicine/s/code-sharing-guidance#loc-data-availability-statement-examples | We have included in our Data Availability Statement the location of the project source code.  All analysis code for FI-Lab is available at GitHub (https://github.com/jhfl2/FI-Lab-Recipe). |
| ETHICS AND CONSENT * Please revise for use of patient-centered language. Please note that patient-centered language is constructed with the use of post-modified nouns (e.g. 'patients with psoriasis’ (or similar) instead of ‘psoriasis patients’) putting the person first in the sentence structure. | Done. |
| FIGURES  * Please show graph axes beginning at zero. If this is not possible, please show a break in the axis. | Thank you. We have added an axis break to Figure 2 along with a corresponding note at the bottom of the figure. Breaks are not relevant to the other main text figures. For supplemental figures presenting model fit, adjusting the axes to start at zero would greatly compress the information presented and hinder interpretation. The remaining axis ranges of existing figures best fit the visualizations of the presented differences (for example, figures of hazard ratios logically start at a reference of 1.0). |
| * When a p value is given, please specify the statistical test used to determine it in the legend. | N/A |
| * Please consider avoiding the use of red and green in order to make your figure more accessible | Thank you. We have avoided the use of red completely, and use green only sparingly, typically within multicolour gradients. Because interpretation of the figures does not require red-green discrimination, we have retained our existing colour scheme.  Please note that we have also fixed a small text misalignment in Supplementary Figures S5-S8 so that the labels are easier to read. |
| TABLES  * Please provide the unadjusted comparisons as well as the adjusted comparisons in all relevant Tables | N/A - there are no adjusted analyses presented in the tables; results reflect summary statistics. |
| * Please specify the variables controlled for in all relevant Tables | N/A - there are no adjusted analyses presented in the tables; results reflect summary statistics. |
| * Please consider if moving some of the figures shared in the supplementary information into the main text would aid the reader. I am asking specifically for Sup Fig 1 (STROBE flow diagram) – I think moving it to the method section as Figure 1 will add clarity in the method section and provide quick overview of the work. | Thank you for your suggestion. We already include the initial and final sample sizes in the Methods section of the manuscript, which we believe is the key information necessary for readers to interpret our study findings. Adding this figure to the main manuscript would largely duplicate the information already presented in the text. To avoid redundancy, we encourage readers interested in the exclusion values to refer to the supplementary figure. |
| ACKNOWLEDGMENTS * Please include an acknowledgment of study participants. | Done. |
| OBSERVATIONAL, COHORT, CROSS-SECTIONAL, AND CASE CONTROL STUDIES * Thank you for provide RECORD checklist as Supplementary Table S1. We do ask to provide it separately as S1 Checklist file and attach as separate supplementary file. | Thank you. At submission, we will ensure the RECORD checklist is uploaded to the portal as a separate PDF file. |
| * Your study is observational and therefore causality cannot be inferred. Please remove language that implies causality and refer to associations instead. | Done. |

**Reviewer Comments**

Reviewer #3

| **Comment** | **Response** |
| --- | --- |
| I greatly appreciate the authors' very thoughtful response - and Dr. Rockwood's personal input here. The paper is much clearer and the additional analyses are very helpful. The point of "acute" vs "critical" illness unmasking frailty, often in those younger than expected is very nicely now made. This will be a very important contribution to the frailty literature. | Thank you very much. |

Reviewer #4:

| **Comment** | **Response** |
| --- | --- |
| Thank you for this very thoughtful response and further analyses, as well as a helpful outline of the history behind this work (and by extension our field). | Thank you! |
| The stratified results are very interesting, thank you for including them. While the text also mentions them, they do not appear in the supplement that was included in this revision (S4-S7 per line 362 on page 22 of the manuscript). | Thank you. We have added these to the supplement (now numbered Supplementary tables S8 and S9) and have amended the text to reflect this. |
| It would also be helpful for readers to present the hazard ratios of the FI-Lab and UK Biobank FI in a regression of the two together to provide some comparison of the magnitude of association. I hope these points are not troublesome; if addressed I have no further suggestions for this manuscript. | We have added this to the supplementary appendix as Supplementary Table S10, along with the following sentence to the main text.  “When including the FI based on diagnoses/symptoms and the FI-lab in the same model, both were associated with higher 10-year mortality with broadly comparable hazard ratios (Supplementary Table S10).” |

**Technical Edits**

| **Comment** | **Response** |
| --- | --- |
| We note that your Supporting Information files are duplicated on your submission. Please remove any unnecessary or old files from your revision, and make sure that only those relevant to the current version of the manuscript are included. | Thank you. We will ensure that only the files associated with this specific revision are included when submitting in the online portal. |
