# Supplementary material for: Evaluation of the FI-Lab, a laboratory-based, automated frailty index for acute care: A multicohort study
Source: PLoS Med. 2026 Sep 1;23(9):e1005004. doi: 10.1371/journal.pmed.1005004 (PMC13577487; doi:10.1371/journal.pmed.1005004)
Supplement: Supplementary file 6 [file pmed.1005004.s003.docx]

**Response to Reviewers**

**Reviewer 1**

**R1.1 — Competing risks**

*Using standard Cox models for readmission outcomes ignores the strong competing risk of high 1-year mortality (e.g., 7.3% in MIMIC-IV-ED), which likely overestimates readmission risks. Despite citing “computational constraints,” the authors should employ Fine-Gray or cause-specific hazard models (at least in sensitivity analyses) for methodological rigor.*

**Response:** We have run a Fine–Gray model sensitivity analysis using the reference configuration (MIMIC-IV-ED; 25 Common tests; feature floor 15) on a 50,000-patient subset in MINIC. Fine–Gray attenuated the hazard ratio by approximately 2–3% compared with CoxPH hazard ratios we report (30-day readmission SHR 1.29 [95% CI 1.27–1.32] vs CoxPH HR 1.32; 1-year readmission Fine Grey HR 1.32 [1.31–1.34] vs CoxPH HR 1.36)

We have added these results to the Limitations paragraph. We note that each of these sensitivity analyses took more than one hour of time on the 50,000-patient subset, and we would be unable to extend them to all cohorts and configurations within the revision window. We have retained cause-specific hazards as the primary analysis. Our position is that the scientific question we are addressing is not the absolute probability of readmission in the presence of competing mortality, but whether the FI-Lab indexes the underlying physiological property that makes readmission more likely.

**R1.2 — Age-stratified reference ranges**

*FI-Lab relies on standard laboratory reference ranges, which are typically for healthy adults and lack age stratification. The authors should discuss how this binary thresholding might misclassify normal age-related physiological changes as “deficits” and affect construct validity in older adults.*

**Response:** We have elected not to introduce age-stratified reference ranges. The point of the FI-Lab described here is to be a basic universal measure: a proportion of abnormal laboratory values that can be calculated identically at any site, in any country, for any adult, from routinely collected blood tests. Fine-tuning or adjustment for age, population, sex, etc, sacrifices exactly the property that makes the measure useful. It moves the FI-Lab from being a ballpark index of underlying physiological dysregulation toward being a calibrated classifier, which it is not intended to be and is not needed to be.

We would also gently push back on the implicit assumption that age-related changes are “normal” in a construct-validity sense. Within a deficit-accumulation framework, the drift of laboratory values outside young-adult reference ranges with age is precisely the biological signal the FI-Lab we would like to capture. This is the mechanism by which deficit indices track biological ageing in the first place (Mitnitski et al., multiple cohorts cited in the Introduction). Stratifying by age before counting would attenuate exactly the signal we are interested in.

We have added a sentence to the Limitations explicitly noting that laboratory reference ranges are standard adult ranges, are not age-stratified by design, and reflect our goal of a uniformly computable measure. We hope this acknowledges the reviewer’s substantive point while making the design choice defensible.

**R1.3 — Pre-test probability and community application**

*ED lab tests are ordered with high clinical suspicion. The authors should further discuss whether FI-Lab thresholds validated in this high-pretest-probability environment might overestimate its predictive performance when applied to routine community screening (e.g., UK Biobank).*

**Response:** It is true that tests ordered in the ED context are selected for clinical suspicion, and we discuss this explicitly in the Discussion paragraph beginning “The decline seen when including rare tests…” and in Figure S19, where we show the strong relationship between a test’s rarity of ordering and its abnormality rate when ordered. We agree this selection applies to the ED cohorts.

However, we would note that the UK Biobank analysis is the community-sampling test case the reviewer asks about. UK Biobank participants received a standard laboratory panel as part of baseline assessment regardless of clinical suspicion, and an FI-Lab calculated from that panel remains associated with one-year and ten-year mortality at hazard ratios per standard deviation of 1.87 (95% CI 1.80–1.94) in the combined model. Performance is attenuated relative to the ED cohorts (as the reviewer says) but remains substantial. We have also added a sentence to the Limitations (in combination with our response to R2.6) noting that the smaller effect size in UK Biobank may in part reflect a higher proportion of non-physiologically predictable deaths (e.g. trauma) in a community cohort than in patients presenting to hospital.

**R1.4 — Applicability in resource-constrained settings**

*The claim that FI-Lab is robust across diverse settings is overstated. The cohorts only represent high-income countries (US and UK) with mature EHR systems. Its applicability in resource-constrained environments lacks data support.*

**Response:** The reviewer is correct. We have softened the Conclusion accordingly: “it performs robustly across settings, populations, and ages” has been changed to “it performs robustly across the settings and populations studied.” The existing Limitations sentence noting that our data come from high-income countries with mature EHR infrastructure, which may not generalise to resource-constrained environments, is retained. We also cite Seligman and colleagues’ recent harmonised frailty index work (J Gerontol A, 2025) as an example of the groundwork needed to extend deficit-accumulation measures into LMIC settings.

**R1.5 (minor) — 20–40 tests in the Abstract**

*The finding that predictive discrimination plateaus at 20–40 features is highly valuable. This specific numerical recommendation should be explicitly added to the Abstract’s conclusion to improve clinical utility.*

**Response:** We have added “A core set of 20 - 40 commonly ordered tests is sufficient to capture the signal” to the Abstract Conclusions. We agree this is the most actionable take-away for a reader and we were glad of the prompt. We have also restructured the Abstract’s “Methods” and “Findings” into a single “Methods and Findings” section per PLOS Medicine format.

**Reviewer 2**

**R2.1 — Exclusion numbers**

*Several exclusion criteria were applied (lines 107–110). Please specify the number excluded for each criterion.*

**Response:** Per-criterion exclusion counts are now reported in the supplementary Methods ("Cohort and inclusion/exclusion criteria"). For KCH, the individual criteria are: age <18 or missing/implausible, n = 101,871 (predominantly paediatric); unparseable datetimes, n = 12,868; admitted before the study window (≤31/12/2016), n = 150; non-binary or missing gender, n = 90; death recorded before ED arrival, n = 76; zero or negative length of stay, n = 51; length of stay >1,000 days, n = 43. Criteria overlap and do not sum exactly to the 115,048 records excluded at this stage. Of the 182,052 records subsequently excluded at lab linkage, 10,622 were admitted to hospital, 52 died in the ED, 268 died in hospital, and the remainder were discharged. For MIMIC-IV-ED the only exclusion was lab linkage (425,011 → 227,736); for UK Biobank, blood test availability (502,533 → 492,703).

**R2.2 — Random forest overfitting**

*The ‘Important’ tests were determined based on a random forest model but no details were provided. How was it defined based on the model? what was the underlying data? if using the same data for evaluation, would it be a cause for overfitting?*

**Response:** ‘Importance’ feature ranking was derived from a random forest model trained on MIMIC-IV-ED with one-year mortality as the outcome, using mean decrease in Gini impurity as the ranking statistic as per supplemental (supplemental text: tests ranked by prognostic importance for 1-year mortality using a random-forest classifier (ranger; permutation importance) trained on eligible labs (conducted in ≥5% of patients) with inverse-prevalence case weights; importance was availability-weighted. Where random-forest importance could not be estimated (e.g., insufficient data), we used a fallback based on abnormality rate weighted by test frequency/availability.). The feature-importance list was then applied to both MIMIC-IV-ED and KCH. We have clarified this in the Methods and we now also state explicitly that using the same cohort for feature selection and evaluation is a potential source of overfitting for the ‘Importance’ strategy in MIMIC-IV-ED.

However, we did not find benefit in this despite the risk of overfitting, and we do not recommend this strategy.

**R2.3 — Feature floor and patient selection**

*Feature floor — presumably this will affect the numbers of patients included in analysis. Please specify this and whether the analyses comparing different feature floors were based on the common set of patients. If the sample size differs, the comparison won’t be fair; otherwise there’d be a selection bias skewed towards the sicker patients.*

**Response**. The reviewer is exactly right: raising the feature floor filters in patients with more tests ordered, who on average have more clinical suspicion of illness (as we discuss in relation to Figure S19). The comparison across floors in our original text was not on a common patient set. This means the apparent improvement in C-statistic with higher floors should not be read as “higher floor is a better measurement” but as “higher floor selects a different patient cohort.”

Changing the floor will not change the FI-lab for any individual patient, it will only change which patients are included in the analysis.

We have rewritten the corresponding cell of Table 2 (“Generally improves” is now “Modest; reflects cohort selection (see Discussion)”) and have more clearly framed the feature floor in the Methods and Discussion as a patient-inclusion filter rather than a measurement-property change.

**R2.4 — Table 2 wording**

*Table 2 is a good summary of the findings, though I'm not sure I agree that increasing feature floor ‘generally improves’ performance. with a few exceptions, the differences in C indices appeared quite small.*

**Response:** Addressed — see R2.3 above. The cell now reads “Modest; reflects cohort selection (see Discussion).”

**R2.5 — Figure 3 axis clarity**

*I also am not sure I fully understand Figure 3. Why the age panels have number of features as x-axis? And the comparison with age would dependent on how age is parameterised. If age is expressed as per 5 years increase the HR would be greater, for example.*

**Response:** We have rewritten the caption to clarify. The x-axis shows the number of laboratory features used to compute the FI-Lab. We plot the age HR across the same x-axis purely as a visual comparator to show how adding more features diminishes the effect of age in the overall model. One might argue that the perfect latent measure of health would account for all the physiological changes associated with age, and thus the significance and effect of age would vanish with the inclusion of such a latent feature.

On the parameterisation point, the reviewer is exactly right and we should have been clearer. All continuous variables (age, FI-Lab, NEWS2) are scaled before modelling and hazard ratios are reported per standard deviation increase. We have now added to the Methods that one standard deviation of the FI-Lab corresponds to approximately 0.10 in the ED cohorts and 0.06 in UK Biobank, and that one standard deviation of age corresponds to approximately 20 years in the ED cohorts. Reporting per-SD rather than per-year or per-0.01 puts all three variables on the same footing for the visual comparison.

**R2.6 — Site-specific reference ranges and non-comparability**

*One notable limitation i think is that each dataset would have own ‘normal’ definition, which made them not comparable. This should at least be acknowledged. Additionally the setting are also not comparable between A&E and UK Biobank, which could explain the lower performance in UKB.*

**Response:** This is, in fact, one of the paper’s central points, and we have rewritten the opening of the Discussion to make it explicit rather than leaving it implied. If the FI-Lab is, as we argue, a latent measure of underlying physiological dysregulation, then it should produce comparable results whether calculated in Boston or London, whether against Beth Israel’s laboratory reference ranges or King’s College’s, and whether against an adult-derived or age-adjusted normal. The observation that hazard ratios for one-year mortality are 2.00 (95% CI 1.99–2.09) in MIMIC-IV-ED and 2.55 (2.42–2.69) in KCH, despite those datasets differing in laboratory platforms, reference-range conventions, case mix, and coding we would argue is the evidence that the technique is measuring something more fundamental than any one site’s definition of normal.

The new opening paragraph of the Discussion articulates this directly: “Critically, this [prognostic] signal remained consistent across the full adult age spectrum, all ethnic groups, and different healthcare systems, and was detectable up to ten years after measurement. This consistency suggests that the FI-Lab captures something fundamental about patient vulnerability, independently of where, when, or in whom it is measured. An optimal FI-Lab balances performance with measurement stability and patient coverage, typically achieved with a core set of 20-40 commonly ordered laboratory tests. We are persuaded that FI-lab captures a latent property of health, which combines both elements of chronic health status and acute illness severity. In this sense the deficit accumulation model could serve as a general-purpose instrument for measuring latent health status, extending the application of FI-labs from geriatrics into routine clinical care.” The new bridging paragraph we have added at the end of the Introduction makes the same point.

On the second half of the reviewer’s comment, we agree this contributes to the smaller UK Biobank effect size, and we have added a Limitations sentence noting that this effect size may in part reflect a higher proportion of non-physiologically-predictable deaths (e.g. trauma) in a community-dwelling cohort compared with hospital presentations.

**Reviewer 3**

**R3.1 — Anchoring on the original Howlett FI-Lab**

*Given existing Lab-FI data from other cohorts, strong rationale is needed for a new framework, particularly one that appears to be validated on mortality specifically (eg Methods line 125 ‘‘Important’’ tests for mortality) when frailty is distinctly a syndrome of vulnerability that predicts mortality but is not defined by it. Better clarification for how the Lab-FI components were selected is needed to frame this as a frailty tool. I suggest an anchor on the original FI-Lab developed by Howlett, Rockwood et al. Otherwise, this becomes yet another FI that may or may not be useful.*

**Response:** We partially concede this point. We agree wholeheartedly with the reviewer that the work should anchor on the deficit-accumulation technique established by Howlett, Rockwood and colleagues. We have made this anchor explicit in the Methods (“…an FI-Lab score was calculated following the deficit-accumulation technique established by Howlett and colleagues…”) and in the new opening paragraph of the Discussion, which positions the present work as an extension of Howlett’s technique from the study of ageing into routine clinical care.

However, we do not want to frame FI-lab as solely a frailty tool but as a latent-health-factor measure—an index of underlying physiological dysregulation, of which clinical frailty in older adults is one manifestation and acute illness severity is another. We agree that “frailty is distinctly a syndrome of vulnerability that predicts mortality but is not defined by it” and this FI-Lab is not offered solely as a classifier, say for triaging patients in ED. We envisage a broader use to measure some factor of general health status across ages and settings. The ‘Importance’ selection ranked against mortality is included only as a ceiling comparator; the practical recommendation of the paper is the ‘Common’ strategy, which is entirely agnostic to outcome.

We have revised the Introduction, Discussion and Conclusion to set out this framing more explicitly. We have been careful not to dilute the debt the present work owes to Howlett’s original FI-Lab framework. The contribution we claim here is its generalisation and the demonstration that it works outside the case of frailty screening in older adults.

**R3.2 — Comparison to eFI**

*Moreover, comparison to NEWS2 makes this a very UK specific analysis. It would strengthen the results considerably to compare the Lab-FI to an eFI which is more broadly used for frailty assessment (vs mortality risk, as noted above).*

**Response:** The eFI (electronic Frailty Index) is a frailty screening tool for older adults constructed from primary care Read/SNOMED codes for chronic conditions, symptoms and functional impairments (Clegg et al., Age Ageing 2016). It is a different construct, derived from a different data source, intended for a different use case, and validated in a different population (older community-dwelling adults). The present work is not a frailty screening tool, does not rely on clinician-coded diagnoses, is not restricted to older adults, and is situated in the emergency department and general adult population, settings where eFI is neither available nor intended to be used.

On the NEWS2 comparison being UK-specific: NEWS2 is the point-of-care deterioration score used across the NHS and serves as the appropriate comparator here because it is what clinicians can currently reference when a patient presents to the ED—that is the gap the FI-Lab is intended to address. We would note also that NEWS and NEWS2 have been widely adopted and are very similar to tools used outside the UK (USA, Canada, Australia, multiple European systems, as well as a WHO version).

We have added language to the Discussion making the choice of comparator explicit: we are benchmarking against what is currently used at the point of care (NEWS2) rather than against what is used for frailty screening in primary care (eFI), because the intended use-case of the FI-Lab described here is acute, not longitudinal primary-care frailty.

**R3.3 — Vital signs and toxicology in the FI-Lab**

*Given the text in the manuscript, I was surprised to see that the Lab-FI includes biometric measures such as BP and HR which vary at times minute to minute. Additionally, inclusion of potentially toxic exposures (ethanol, acetaminophen) will be rarely measured outside of the emergency department making these less generalizable. Also, as above, how are these measures of frailty?*

**Response:** We have clarified this in the Methods. The reference configuration recommended in the Abstract (25 Common tests, feature floor 15) is the 25 most commonly ordered laboratory tests; vital signs are evaluated in a sensitivity analysis (and are discussed in Results, “Sensitivity Analyses”). The reason we include them is precisely that the reviewer’s characterisation—that they vary minute-to-minute—is the clinical intuition the FI-Lab challenges: a patient whose heart rate, blood pressure and respiratory rate are all simultaneously outside reference ranges is, in the deficit-accumulation framework, a patient whose physiology has lost homeostatic capacity. That is a property of the patient, not of the moment. Our data suggest vital signs add modest discrimination for in-hospital mortality but nothing material for 1-year mortality, which supports the interpretation that they encode short-horizon acute severity (complementary to, but distinct from, the deeper physiological reserve signal that drives the 1-year and 10-year predictions).

On toxicology: the reviewer is correct that ethanol and acetaminophen are rarely measured outside the ED, and they would not be part of our recommended configuration as they were not among the top tests ordered. However, if they were ordered for all patients presenting to an ED we would recommend including them, since they would encode useful information about the current physiological status of that patient.

On the frailty framing question (“how are these measures of frailty?”), please see our response to R3.1. We are not claiming the FI-Lab is a frailty measure in the narrow syndrome-of-vulnerability-in-older-adults sense; we are claiming it is a measure of underlying physiological dysregulation of which frailty is one manifestation. Within that framing, vital signs and exposures are manifestations of the same underlying dysregulation, which is why they are included in a deficit-accumulation index even if their relevance to the clinical phenotype of frailty is less direct.

**Reviewer 4**

**R4.1 — Acute-phase reactants may be inappropriately normal in frailty**

*The first point is conceptual and should be at least mentioned in the discussion, though there is no clear resolution. Frailty is meant to capture reduced ability to respond to physiologic stressors. This suggests that values for acute phase reactants, such as WBC or lactate, might be inappropriately normal in acute illness in frail people. By relying largely on values from ED/A&E visits, this index may represent severity of acute illness rather than frailty. This may explain some of the differences in discrimination among the UK Biobank, KCH, and MIMIC-IV. This might also explain some of the decline in AUC with additional labs in MIMIC-IV.*

**Response: See previous responses**

At least one author had not considered fully that acute phase responses might be inappropriately normal in some frail older adults, and this might explain why we observed some diminution of the FI-lab in these groups in terms of discriminative performance.

It is true that “by relying largely on values from ED/A&E visits, this index may represent severity of acute illness rather than frailty.” (see responses to reviewer 3). However, in UK Biobank FI-Lab remained associated with mortality at hazard ratios per SD of 1.87 (95% CI 1.80–1.94) and at time horizons of up to ten years. If the signal in the ED cohorts were purely acute illness severity, it should be absent in UK Biobank; it is not. It is likely a combination of both. We elaborate on these points in the Key Findings section.

**R4.2 — FI distribution, HR scale, bootstrap CIs**

*Second, the details of how frailty was measured and used in the Cox regressions are unclear. The makeup of the 25-items FI used in the Cox regressions should be clearly presented in the appendix. The scale should also be made clear: an FI is typically a proportion between 0 and 1, however Figure 1 suggests it was measured on a percentage point scale. Cox regressions yielded HRs of ~2, a very strong relationship on the percentage point scale, but on the 0-1 scale implies an HR of 1.07 for a 0.1-increment change in FI, similar to the NEWS score. Finally, the FI distribution is a non-normal and asymmetric; bootstrapped confidence intervals may be more appropriate than analytic ones.*

**Response:**

(i) The 25-item reference configuration. The full list of candidate tests and the 25 ‘Common’ tests used in the reference configuration are in the Supplementary Appendix. We will highlight this more prominently in the revised Methods text.

(ii) Scale. We report hazard ratios per standard deviation increase throughout, not per 0.1 increment and not per 0.01 increment. We have added to the Methods that one standard deviation of the FI-Lab corresponds to approximately 0.10 in the ED cohorts and approximately 0.06 in UK Biobank, and that one standard deviation of age corresponds to approximately 20 years in the ED cohorts. The reviewer’s arithmetic on the per-0.1 scale is right as an order of magnitude: a per-SD HR of around 2.00 in the ED cohorts corresponds to approximately HR 1.07 per 0.01 increase or approximately HR 2.00 per 0.10 increase. The per-SD reporting is deliberate because it puts FI-Lab, age and NEWS2 on the same footing for the visual comparison in Figure 3. Figure 1 shows the raw FI-Lab score on the 0–1 scale with percentage labelling for readability; we have clarified the caption.

(iii) Bootstrap CIs. The reviewer is right that the FI-Lab distribution is non-normal and asymmetric. However, in practice, at the sample sizes here (n > 100,000 with several thousand events in each ED cohort) the sampling distribution of the log hazard ratio is effectively Gaussian by the central limit theorem regardless, and analytic Wald intervals and percentile bootstrap intervals would be expected to agree to three decimal places.

**R4.3 — Comparison with traditional FI and physical frailty in UKB**

*Third and finally, this is a paper investigating a different approach to assessing frailty and it is worth including a comparison with a more traditional FI and possibly with physical frailty. This is likely most feasible in the UK Biobank data. Both FIs and physical frailty have been developed in this cohort (see Williams DM et al. J Gerontol A Biol Sci Med Sci. 2019;74(4):582-587. and Hanlon P et al. Lancet Public Health. 2018;3(7):e323-e332.).*

**Response:** We have considered this carefully and decline this specific comparison, for the same framework reason we decline the eFI comparison (R3.2). The FI-Lab described here is not offered as a frailty measure in the sense that the Hanlon/Williams UK Biobank FIs are frailty measures. Those indices are developed and validated against classical frailty phenotypes in older adults (Fried-type physical frailty, Rockwood-type cumulative-deficit FI from self-report and clinical variables) and are the appropriate tools for identifying frailty. If this paper were a claim that the FI-Lab is a superior frailty screen, the comparison the reviewer proposes would be essential. It is not. We also think such a comparison would be a distraction from the central argument of the paper rather than an addition to it.

We considered running the comparison anyway for inclusion in this response, if not in the paper. However, UK Biobank has imposed a platform-wide pause on researcher access following a recent data-handling incident, and so we have been unable to do so within the revision window.

R4.4 — Figure S2 removal

*Figure S2 is not referenced in the manuscript and looks identical to Figure 1. It should be removed.*

**Response:** Done — Figure S2 has been removed from the Supplementary Appendix.

**Summary of changes to the manuscript**

The principal revisions are as follows; all changes are tracked in the marked-up manuscript and supplementary appendix.

1. Abstract restructured into “Methods and Findings” format per PLOS requirements, with explicit recommendation of a 20–40-test core set.

2. New bridging paragraph at the end of the Introduction introducing the latent-health-factor framing.

3. Explicit anchor on Howlett’s deficit-accumulation technique in the Methods.

4. Per-SD scaling clarification added to the Methods.

5. Table 2, Feature floor × Effect on Performance cell rewritten to reflect cohort selection rather than measurement improvement.

6. New opening paragraph of the Discussion articulating the latent-health-factor framing explicitly.

7. New paragraph at the end of Key Findings addressing acute-phase response attenuation within the latent-health-factor framing (Reviewer 4).

8. Limitations: Cox-vs-competing-risk sentence replaced with Fine–Gray / Aalen–Johansen sensitivity analysis results (Reviewer 1).

9. Limitations: new sentence noting reference ranges are standard adult ranges by design.

10. Limitations: new sentence on probable contribution of non-biologically-predictable deaths to smaller UK Biobank effect size.

11. Conclusion softened: “robustly across settings, populations, and ages” to “robustly across the settings and populations studied” (Reviewer 1).

12. Supplementary: duplicate Figure S2 removed (Reviewer 4).

We are grateful to the reviewers and to the editorial team for the time and attention given to this manuscript. We believe the revised version is substantially clearer and, in particular, better articulates what the FI-Lab is measuring and what it is not.

**General Editorial Requests:**

* We ask every co-author listed on the manuscript to fill in a contributing author statement, making sure to declare all competing interests. If any of the co-authors have not filled in the statement, we will remind them to do so when the paper is revised. If all statements are not completed in a timely fashion this could hold up the re-review process. If new competing interests are declared later in the revision process, this may also hold up the submission. Should there be a problem getting one of your co-authors to fill in a statement we will be in contact. Please do not add or remove authors without first discussing this with the handling editor. You can see our competing interests policy here: [http://journals.plos.org/plosmedicine/s/competing-interests](https://can01.safelinks.protection.outlook.com/?url=http%3A%2F%2Fjournals.plos.org%2Fplosmedicine%2Fs%2Fcompeting-interests&data=05%7C02%7Calexandra.vandervalk%40nshealth.ca%7Cd7607787d20e4838439c08dea9f22072%7C8eb23313ce754345a56a297a2412b4db%7C0%7C0%7C639135054593019066%7CUnknown%7CTWFpbGZsb3d8eyJFbXB0eU1hcGkiOnRydWUsIlYiOiIwLjAuMDAwMCIsIlAiOiJXaW4zMiIsIkFOIjoiTWFpbCIsIldUIjoyfQ%3D%3D%7C0%7C%7C%7C&sdata=x9Ue7AQAsSahb2mgQVgh4TlkLCHxVounYjjnUuM9%2BPs%3D&reserved=0).

**Reply:** We have written to all co-authors and asked them either ot confirm with me (Rockwood) or copy me when they submit to you.

* Please upload any figures associated with your paper as individual TIF or EPS files with 300dpi resolution at resubmission; please read our figure guidelines for more information on our requirements: [http://journals.plos.org/plosmedicine/s/figures](https://can01.safelinks.protection.outlook.com/?url=http%3A%2F%2Fjournals.plos.org%2Fplosmedicine%2Fs%2Ffigures&data=05%7C02%7Calexandra.vandervalk%40nshealth.ca%7Cd7607787d20e4838439c08dea9f22072%7C8eb23313ce754345a56a297a2412b4db%7C0%7C0%7C639135054593068277%7CUnknown%7CTWFpbGZsb3d8eyJFbXB0eU1hcGkiOnRydWUsIlYiOiIwLjAuMDAwMCIsIlAiOiJXaW4zMiIsIkFOIjoiTWFpbCIsIldUIjoyfQ%3D%3D%7C0%7C%7C%7C&sdata=0fQaGM60Gc96Vx%2FGowszsh5f7Ka%2FLXLEzwt4iJqKQGQ%3D&reserved=0). While revising your submission, we strongly recommend that you use PLOS's NAAS tool ([https://ngplosjournals.pagemajik.ai/artanalysis](https://can01.safelinks.protection.outlook.com/?url=https%3A%2F%2Fngplosjournals.pagemajik.ai%2Fartanalysis&data=05%7C02%7Calexandra.vandervalk%40nshealth.ca%7Cd7607787d20e4838439c08dea9f22072%7C8eb23313ce754345a56a297a2412b4db%7C0%7C0%7C639135054593113225%7CUnknown%7CTWFpbGZsb3d8eyJFbXB0eU1hcGkiOnRydWUsIlYiOiIwLjAuMDAwMCIsIlAiOiJXaW4zMiIsIkFOIjoiTWFpbCIsIldUIjoyfQ%3D%3D%7C0%7C%7C%7C&sdata=TZLzxgqoaz5kPNHokRp3h5hjxvR%2BGLu%2FRDymddhWkNY%3D&reserved=0)) to test your figure files. NAAS can convert your figure files to the TIFF file type and meet basic requirements (such as print size, resolution), or provide you with a report on issues that do not meet our requirements and that NAAS cannot fix.

After uploading your figures to PLOS's NAAS tool - [https://ngplosjournals.pagemajik.ai/artanalysis](https://can01.safelinks.protection.outlook.com/?url=https%3A%2F%2Fngplosjournals.pagemajik.ai%2Fartanalysis&data=05%7C02%7Calexandra.vandervalk%40nshealth.ca%7Cd7607787d20e4838439c08dea9f22072%7C8eb23313ce754345a56a297a2412b4db%7C0%7C0%7C639135054593157197%7CUnknown%7CTWFpbGZsb3d8eyJFbXB0eU1hcGkiOnRydWUsIlYiOiIwLjAuMDAwMCIsIlAiOiJXaW4zMiIsIkFOIjoiTWFpbCIsIldUIjoyfQ%3D%3D%7C0%7C%7C%7C&sdata=ow%2FWNaeFr6FH4iksfh%2BeJM137kPbUhJeAlyHinVb9WU%3D&reserved=0), NAAS will process the files provided and display the results in the "Uploaded Files" section of the page as the processing is complete.
If the uploaded figures meet our requirements (or NAAS is able to fix the files to meet our requirements), the figure will be marked as "fixed" above. If NAAS is unable to fix the files, a red "failed" label will appear above.
When NAAS has confirmed that the figure files meet our requirements, please download the file via the download option, and include these NAAS processed figure files when submitting your revised manuscript.

**Reply:** Figures have been uploaded as TIF.

* Please ensure that the paper adheres to the PLOS Data Availability Policy (see [http://journals.plos.org/plosmedicine/s/data-availability)](https://can01.safelinks.protection.outlook.com/?url=http%3A%2F%2Fjournals.plos.org%2Fplosmedicine%2Fs%2Fdata-availability)&data=05%7C02%7Calexandra.vandervalk%40nshealth.ca%7Cd7607787d20e4838439c08dea9f22072%7C8eb23313ce754345a56a297a2412b4db%7C0%7C0%7C639135054593199344%7CUnknown%7CTWFpbGZsb3d8eyJFbXB0eU1hcGkiOnRydWUsIlYiOiIwLjAuMDAwMCIsIlAiOiJXaW4zMiIsIkFOIjoiTWFpbCIsIldUIjoyfQ%3D%3D%7C0%7C%7C%7C&sdata=gfUEpMAiI%2BHVTXmd2CsbYT4xkY0hOeT%2Fu7I1RVUFKp4%3D&reserved=0), which requires that all data underlying the study's findings be provided in a repository or as Supporting Information. For data residing with a third party, authors are required to provide instructions with contact information (web or email address) for obtaining the data. Please note that a study author cannot be the contact person for the data. PLOS journals do not allow statements supported by "data not shown" or "unpublished results." For such statements, authors must provide supporting data or cite public sources that include it.

**Reply:** The manuscript adheres to the Data Availability policy.

* We expect all researchers with submissions to PLOS in which author-generated code underpins the findings in the manuscript to make all author-generated code available without restrictions upon publication of the work. In cases where code is central to the manuscript, we may require the code to be made available as a condition of publication. Authors are responsible for ensuring that the code is reusable and well documented. Please make any custom code available, either as part of your data deposition or as a supplementary file. Please add a sentence to your data availability statement regarding any code used in the study, e.g. "The code used in the analysis is available from Github [URL] and archived in Zenodo [DOI link]" Please review our guidelines at [https://journals.plos.org/plosmedicine/s/materials-software-and-code-sharing](https://can01.safelinks.protection.outlook.com/?url=https%3A%2F%2Fjournals.plos.org%2Fplosmedicine%2Fs%2Fmaterials-software-and-code-sharing&data=05%7C02%7Calexandra.vandervalk%40nshealth.ca%7Cd7607787d20e4838439c08dea9f22072%7C8eb23313ce754345a56a297a2412b4db%7C0%7C0%7C639135054593240312%7CUnknown%7CTWFpbGZsb3d8eyJFbXB0eU1hcGkiOnRydWUsIlYiOiIwLjAuMDAwMCIsIlAiOiJXaW4zMiIsIkFOIjoiTWFpbCIsIldUIjoyfQ%3D%3D%7C0%7C%7C%7C&sdata=8Ie3ILXQxXIo9PytB45o3ymrZ7vUfeeV3fjnoky2b30%3D&reserved=0) and ensure that your code is shared in a way that follows best practice and facilitates reproducibility and reuse. Because Github depositions can
be readily changed or deleted, we encourage you to make a permanent DOI'd copy (e.g. in Zenodo) and provide the URL.

**Reply:** A Data Availability statement is included in the manuscript.

* Please ensure that the study is reported according to the relevant guideline and include the completed [XXXX] checklist as Supporting Information. When completing the checklist, please use section and paragraph numbers, rather than page numbers. Please add the following statement, or similar, to the Methods: "This study is reported as per [XXXX] guideline (S1 Checklist)."

**Reply:** The manuscript adheres to relevant guidelines and the appropriate checklist is provided.

FORMATTING – GENERAL

* Abstract: Please structure your abstract using the PLOS Medicine headings (Background, Methods and Findings, Conclusions). Please combine the Methods and Findings sections into one section.

**Reply:** Abstract has been structured as required.

* At this stage, we ask that you include a short, non-technical Author Summary of your research to make findings accessible to a wide audience that includes both scientists and non-scientists. The Author Summary should immediately follow the Abstract in your revised manuscript. This text is subject to editorial change and should be distinct from the scientific abstract. Ideally each sub-heading should contain 2-3 single sentence, concise bullet points containing the most salient points from your study. In the final bullet point of 'What Do These Findings Mean?', please include the main limitations of the study in non-technical language. Please see our author guidelines for more information: [https://can01.safelinks.protection.outlook.com/?url=https%3A%2F%2Fjournals.plos.org%2Fplosmedicine%2Fs%2Frevising-your-manuscript%23loc-author-summary&data=05%7C02%7CAlexandra.vanderValk%40nshealth.ca%7Cb7b04645121a42cbe3ba08dea78f8d32%7C8eb23313ce754345a56a297a2412b4db%7C0%7C0%7C639132432461960906%7CUnknown%7CTWFpbGZsb3d8eyJFbXB0eU1hcGkiOnRydWUsIlYiOiIwLjAuMDAwMCIsIlAiOiJXaW4zMiIsIkFOIjoiTWFpbCIsIldUIjoyfQ%3D%3D%7C0%7C%7C%7C&sdata=JSc45nzjT0F0J3SmFPzCTNjVs2N3F3DDm%2BJZ4fmUR84%3D&reserved=0](https://can01.safelinks.protection.outlook.com/?url=https%3A%2F%2Fjournals.plos.org%2Fplosmedicine%2Fs%2Frevising-your-manuscript%23loc-author-summary&data=05%7C02%7CAlexandra.vanderValk%40nshealth.ca%7Ce40526e083ed482d46ee08dea85468fe%7C8eb23313ce754345a56a297a2412b4db%7C0%7C0%7C639133277640471236%7CUnknown%7CTWFpbGZsb3d8eyJFbXB0eU1hcGkiOnRydWUsIlYiOiIwLjAuMDAwMCIsIlAiOiJXaW4zMiIsIkFOIjoiTWFpbCIsIldUIjoyfQ%3D%3D%7C0%7C%7C%7C&sdata=%2Fg1AUW5pkHCfO8%2BjLkwWS7Hcp2EUlpg1bI4NXh7U%2BQg%3D&reserved=0).

**Reply:** The following Author Summary has been added to the Revised manuscript.

**Why was this study done?**

"How sick is this person?" is a question that health care providers ponder dozens of times a day. Despite all data recorded for each patient, we lack good ways to answer the question. We wanted to see if we could create a quick, at-a-glance summary, using data already in a patient's record to help answer just how sick a specific person might be.

We know that counting the proportion of tests which are abnormal (called a laboratory frailty index, or FI-Lab for short) is an effective way of measuring frailty in older adults. We asked whether the FI-lab approach could work as a general measure of how unwell a person is, across all adult ages and in different healthcare settings.

**What did the researchers do and find?**

We used already-collected healthcare data for almost 900,000 adults from three datasets: emergency department visits in Boston (MIMIC-IV-ED) and London (King's College Hospital), and a UK community cohort (UK Biobank).

We tested different recipes for making an FI-Lab, varying which tests to include, how many, and how few were required. We compared how well these different approaches worked against each other, as well as against other general measures health: age, and standard health deterioration scores.

A simple FI-Lab built from around 25 commonly-ordered blood tests is as strong an indicator as someone's age to assess their risk of dying, being admitted to hospital, or being readmitted, whereas common deterioration risk scores are much worse at these tasks. The FI-Lab works regardless of age, sex, ethnicity, or either of the healthcare systems we tested.

**What do these findings mean?**

FI-Lab provides a fast, automatic, transparent gauge of how unwell a patient is, without needing any extra testing or a bedside assessment. The same idea extends beyond blood tests to vital signs and, in principle, any routinely collected data point that can be classified as normal or abnormal.

An idea originally developed for older people works as a broader indicator of underlying health across the entire adult lifespan. It could aid clinicians in their assessment of patients.

This was a retrospective study using data from two high-income countries. Even though it shows the score reliably tracks poor outcomes, it does not yet prove that having access to it in real time would change decisions or improve patient care.

* Please express the main results with 95% CIs as well as p values. When reporting p values please report as p<0.001 and where higher as the exact p value p=0.002, for example. Throughout, suggest reporting statistical information as follows to improve clarity for the reader "22% (95% CI [13%,28%]; p</=)". Please be sure to define all numerical values at first use.

**Reply:** Done

* Please include page numbers and line numbers in the manuscript file. Use continuous line numbers (do not restart the numbering on each page).

**Reply:** Done

* Please cite the reference numbers in square brackets. Citations should precede punctuation.

**Reply:** Done

FIGURES AND TABLES
* Please provide titles and legends for all figures and tables (including those in Supporting Information files).

**Reply:** Done

* Please define all abbreviations used in each figure/table (including those in Supporting Information files).

**Reply:** Done

* Please consider avoiding the use of red and green in order to make your figure more accessible to those with color blindness.

**Reply**: Only shades of green, blue, and yellow on slides.

SUPPLEMENTARY MATERIAL
* Please note that supplementary material will be posted as supplied by the authors. Therefore, please amend it according to the relevant comments outlined here.

* Please cite your Supporting Information as outlined here: [https://journals.plos.org/plosmedicine/s/supporting-information](https://can01.safelinks.protection.outlook.com/?url=https%3A%2F%2Fjournals.plos.org%2Fplosmedicine%2Fs%2Fsupporting-information&data=05%7C02%7Calexandra.vandervalk%40nshealth.ca%7Cd7607787d20e4838439c08dea9f22072%7C8eb23313ce754345a56a297a2412b4db%7C0%7C0%7C639135054593326052%7CUnknown%7CTWFpbGZsb3d8eyJFbXB0eU1hcGkiOnRydWUsIlYiOiIwLjAuMDAwMCIsIlAiOiJXaW4zMiIsIkFOIjoiTWFpbCIsIldUIjoyfQ%3D%3D%7C0%7C%7C%7C&sdata=Bdx2lS2ucTXVEBLBg0H8g0hESmWvbK89wQWE9cjxcss%3D&reserved=0)

**Reply:** Supplementary material referenced as appropriate.

REFERENCES
* PLOS uses the numbered citation (citation-sequence) method and first six authors, et al.

* Please ensure that journal name abbreviations match those found in the National Center for Biotechnology Information (NCBI) databases ([http://www.ncbi.nlm.nih.gov/nlmcatalog/journals](https://can01.safelinks.protection.outlook.com/?url=http%3A%2F%2Fwww.ncbi.nlm.nih.gov%2Fnlmcatalog%2Fjournals&data=05%7C02%7Calexandra.vandervalk%40nshealth.ca%7Cd7607787d20e4838439c08dea9f22072%7C8eb23313ce754345a56a297a2412b4db%7C0%7C0%7C639135054593370987%7CUnknown%7CTWFpbGZsb3d8eyJFbXB0eU1hcGkiOnRydWUsIlYiOiIwLjAuMDAwMCIsIlAiOiJXaW4zMiIsIkFOIjoiTWFpbCIsIldUIjoyfQ%3D%3D%7C0%7C%7C%7C&sdata=HsloWvPgRDvekhwfxdtMwXAdjPblEKcs8ZCEM2hhyiQ%3D&reserved=0)), and are appropriately formatted and capitalised.

* Where website addresses are cited, please include the complete URL and specify the date of access (e.g. [accessed: 12/06/2023]).

* Please also see [https://journals.plos.org/plosmedicine/s/submission-guidelines#loc-references](https://can01.safelinks.protection.outlook.com/?url=https%3A%2F%2Fjournals.plos.org%2Fplosmedicine%2Fs%2Fsubmission-guidelines%23loc-references&data=05%7C02%7Calexandra.vandervalk%40nshealth.ca%7Cd7607787d20e4838439c08dea9f22072%7C8eb23313ce754345a56a297a2412b4db%7C0%7C0%7C639135054593415398%7CUnknown%7CTWFpbGZsb3d8eyJFbXB0eU1hcGkiOnRydWUsIlYiOiIwLjAuMDAwMCIsIlAiOiJXaW4zMiIsIkFOIjoiTWFpbCIsIldUIjoyfQ%3D%3D%7C0%7C%7C%7C&sdata=0sP8VPnB5bxhzIZINuQ6OsQjvn9RKb5tWcuXlveOd8A%3D&reserved=0) for further details on reference formatting.

**Reply:** Formatted according to the journal website’s specified *Vancouver* style, applied automatically using Zotero software.

STUDY TYPE-SPECIFIC REQUESTS - please note not all might apply:

MODELLING STUDIES

The following list is derived from Geoffrey P Garnett, Simon Cousens, Timothy B Hallett, Richard Steketee, Neff Walker. Mathematical models in the evaluation of health programmes. (2011) Lancet DOI:10.1016/S0140-6736(10)61505-X:

1. If pertinent, please provide a diagram that shows the model structure, including how the natural history of the disease is represented, the process and determinants of disease acquisition, and how the putative intervention could affect the system.

**Reply**: Not applicable i.e., this is not creating a model of disease processes with intermediate steps. See below, points 9 and 10. The way we came to this was seeing that it worked in the KCH data in older adults, and given that, albeit to varying degrees, we were each familiar with FI-Labs, we wondered about examining the context in the setting of acute illness, across the adult age spectrum. From that, we have now made clear (lines 97-98), we wanted to check out whether this held in a community setting. In this sense, Hugh’s hackathon idea about the use of the FI-Lab more generally was translated into first its use at a much larger scale than had been employed previously (the two Age and Ageing papers, plus now here) and then across the adult life course (MIMIC-ED and KCH).

2. Please provide a complete list of model parameters, including clear and precise descriptions of the meaning of each parameter, together with the values or ranges for each, with justification or the primary source cited and important caveats about the use of these values noted.

**Reply:** As we read it, the question refers to explicit parameters that have been used to construct an explicit model of a process. That was not exactly what we were doing, but we note that all model parameters and their respective reference ranges are available in the Supplement file (see section entitled “Supplementary Methods” pp23-21). For each step, justifications and/or sources are discussed as appropriate.

For clarity: The Supplement contains all of the analyses that we performed.

3. Please provide a clear statement about how the model was fitted to the data, including goodness-of-fit measure, the numerical algorithm used, which parameter varied, constraints imposed on parameter values, and starting conditions.

**Reply:** We have added more detail regarding the model used, its characteristics, and relevant constraints (lines 148-151; 164). We emphasize, however, that the purpose of the model was to validate the FI-Lab tool as a measure, not to use the model for prediction.

4. For uncertainty analyses, please state the sources of uncertainties quantified and not quantified [can include parameter, data, and model structure].

**Reply**: Uncertainty and robustness are discussed on page 24 of the Supplement file. The results of this stability vs noise analysis for the constructed FI-Lab are presented in the Supplement in Figure S4.

5. Please provide sensitivity analyses to identify which parameter values are most important in the model. Uncertainty estimates seek to derive a range of credible results on the basis of an exploration of the range of reasonable parameter values. The choice of method should be presented and justified.

**Reply**: Sensitivity analyses are discussed in detail and the results presented in a dedicated subsection of the manuscript Pages 18-19, lines 280-297, with references to Supplementary Table 1, Table 2, and Figure 17).

6. Please discuss the scientific rationale for the choice of model structure and identify points where this choice could influence conclusions drawn. Please also describe the strength of the scientific basis underlying the key model assumptions.

**Reply:** As above

7. For studies that develop a prediction model or evaluate its performance, please ensure that the study is reported according to the TRIPOD statement (<https://can01.safelinks.protection.outlook.com/?url=https%3A%2F%2Fwww.equator-network.org%2Freporting-guidelines%2Ftripod-statement&data=05%7C02%7Ckenneth.rockwood%40nshealth.ca%7C1f546ab490df42ab56f708dea782a426%7C8eb23313ce754345a56a297a2412b4db%7C0%7C0%7C639132376710859212%7CUnknown%7CTWFpbGZsb3d8eyJFbXB0eU1hcGkiOnRydWUsIlYiOiIwLjAuMDAwMCIsIlAiOiJXaW4zMiIsIkFOIjoiTWFpbCIsIldUIjoyfQ%3D%3D%7C0%7C%7C%7C&sdata=vVoGYF3P5nLP4ZAdCLpaOntdloY8gpukYIB%2BFrWt0So%3D&reserved=0>) and include the completed checklist as Supporting Information. Please add the following statement, or similar, to the Methods: "This study is reported as per the Transparent Reporting of a Multivariable Prediction Model for Individual Prognosis Or Diagnosis (TRIPOD) statement (S1 Checklist)." For studies using machine learning, please use the TRIPOD-AI checklist. When completing the checklist, please use section and paragraph numbers, rather than page numbers.

**Reply**: Not applicable. As above, we see the work here as testing whether what we have is a physiological measure. As with a great deal of work on frailty, the goal is not to develop a mortality model. We recognize however that as an adverse outcome, mortality has the considerable advantages of being clinically relevant, verifiable, non-arbitrary, subject to mandatory reporting, and dichotomous.

OBSERVATIONAL STUDIES

8. Abstract: Please include the study design, population and setting, number of participants, years during which the study took place (enrollment and follow up), length of follow up, and main outcome measures.

**REPLY:** These aspects are included in our Abstract**.**

9. Please ensure that the study is reported according to the STROBE (or appropriate STOBE extension) guideline (available from: <https://can01.safelinks.protection.outlook.com/?url=https%3A%2F%2Fwww.equator-network.org%2Freporting-guidelines%2Fstrobe&data=05%7C02%7Ckenneth.rockwood%40nshealth.ca%7C1f546ab490df42ab56f708dea782a426%7C8eb23313ce754345a56a297a2412b4db%7C0%7C0%7C639132376710785553%7CUnknown%7CTWFpbGZsb3d8eyJFbXB0eU1hcGkiOnRydWUsIlYiOiIwLjAuMDAwMCIsIlAiOiJXaW4zMiIsIkFOIjoiTWFpbCIsIldUIjoyfQ%3D%3D%7C0%7C%7C%7C&sdata=XKhPG5Ued6Kcd95c7WQNlM1kwSBeYh9t12fr8Ds4ApE%3D&reserved=0>) and include the completed STROBE (or STROBE extension) checklist as Supporting Information. Please add the following statement, or similar, to the Methods: "This study is reported as per the Strengthening the Reporting of Observational Studies in Epidemiology (STROBE) guideline (S1 Checklist)." When completing the checklist, please use section and paragraph numbers, rather than page numbers.

* [FOR POPULATION HEALTH/REGISTRY STUDIES] Please ensure that the study is reported according to the RECORD guideline (available from <https://can01.safelinks.protection.outlook.com/?url=https%3A%2F%2Fwww.record-statement.org%2F&data=05%7C02%7Ckenneth.rockwood%40nshealth.ca%7C1f546ab490df42ab56f708dea782a426%7C8eb23313ce754345a56a297a2412b4db%7C0%7C0%7C639132376710801915%7CUnknown%7CTWFpbGZsb3d8eyJFbXB0eU1hcGkiOnRydWUsIlYiOiIwLjAuMDAwMCIsIlAiOiJXaW4zMiIsIkFOIjoiTWFpbCIsIldUIjoyfQ%3D%3D%7C0%7C%7C%7C&sdata=wcRwY0I401EuRsmip662ocrErz0kkESMyubrqjkHMbQ%3D&reserved=0>) and include the completed checklist as Supporting Information. Please add the following statement, or similar, to the Methods: "This study is reported as per the Reporting of Studies Conducted using Observational Routinely-Collected Data (RECORD) guideline (S1 Checklist)." When completing the checklist, please use section and paragraph numbers, rather than page numbers.

* [FOR POPULATION HEALTH ESTIMATES] Please ensure that the study is reported according to the GATHER statement (available from <https://can01.safelinks.protection.outlook.com/?url=https%3A%2F%2Fwww.equator-network.org%2Freporting-guidelines%2Fgather-statement&data=05%7C02%7Ckenneth.rockwood%40nshealth.ca%7C1f546ab490df42ab56f708dea782a426%7C8eb23313ce754345a56a297a2412b4db%7C0%7C0%7C639132376710818525%7CUnknown%7CTWFpbGZsb3d8eyJFbXB0eU1hcGkiOnRydWUsIlYiOiIwLjAuMDAwMCIsIlAiOiJXaW4zMiIsIkFOIjoiTWFpbCIsIldUIjoyfQ%3D%3D%7C0%7C%7C%7C&sdata=bkkq733T7LkoaeAQtvWoBfTss43SyvnUd4J365sIja4%3D&reserved=0>) and include the completed checklist as Supporting Information. Please add the following statement, or similar, to the Methods: "This study is reported as per the Guidelines for Accurate and Transparent Health Estimates Reporting (GATHER) statement (S1 Checklist)." When completing the checklist, please use section and paragraph numbers, rather than page numbers.

**REPLY**: As below, we have followed STROBE/RECORD with that sentence in the Methods, and Figure S1: STROBE flow diagram of the study cohort selection process. (Supplement p1).

This appears to be the appropriate checklist for all of this. By our reading of the GATHER link, what we’ve done does not fit that definition of population health estimates. As we add below, regarding mortality rates, the level of precision that we bring to it is appropriate to the subject matter. Our goal here is to validate that the FI-Lab is capturing an element of physiologically determined lack of resilience and robustness that makes further deficit accumulation likely, especially as the degree of frailty (ageing, deficit accumulation increases). My clinical work (Rockwood writing here), especially years on the Medicine consult team in our Emergency Department, looking at the intersection of acute illness and underlying frailty has informed much of my understanding of how frailty arises. In my experience what I will say next is more detail than a general audience would care for. First though, I acknowledge that I have been privileged to work with talented people with great insights into the physical world – first my late friend and colleague Arnold Mitnitski, who died during the pandemic, and now Andrew Rutenberg, one of the founding members of the new discipline of gerophysics. Without them, I would not have had access to the apparatus that is beginning to reveal what further motivates the present work and its interpretation. A longstanding concern of mine has been the near-tautology of saying that frailty is “accelerated ageing” or “lack of physiological reserve”. This typically goes unchallenged, even in the frailty literature – and perhaps for that reason didn’t make the final cut in our 2024 NEJM review. What we did manage to get into that review though was that frailty (as a multiply determined, age-related state of increased risk) progresses as the ability to resist or repair damage erodes.

What signals that erosion is deficit accumulation, *i.e*., unresisted, unremoved, or unrepaired damage. Damage begets damage – the easiest way to think about this is that at some stage, and quite commonly by chronological age 75 years [to be transparent, we have yet only a preprint on this, under what is turning out to be long-term review - here’s the PMID: 41479451] damage begins to damage the repair processes. This increases recovery time, so diminishing the chance of withstanding further damage. Said damage can be acute or chronic. Not all of that is captured by the FI-Lab, so age remains important. That is the plain words account of what I think is happening here. (Not all environments are equally damaging. That can be captured by deprivation / social vulnerability. We did some adjustment for that in our KCH estimates, but clearly that is a much longer story, and not material at this stage.)

In short, we refer to the STROBE and RECORD guidelines in the manuscript (lines 166-169). We have attached the relevant checklist to our submission files.

* [FOR MEDIATION ANALYSES] We recommend that the study is reported according to the AGReMA statement (<https://can01.safelinks.protection.outlook.com/?url=https%3A%2F%2Fagrema-statement.org%2F%23%3A~%3Atext%3DAGReMA%2520is%2520an%2520evidence-%2520and%2Crandomised%2520trials%2520and%2520observational%2520studies&data=05%7C02%7Ckenneth.rockwood%40nshealth.ca%7C1f546ab490df42ab56f708dea782a426%7C8eb23313ce754345a56a297a2412b4db%7C0%7C0%7C639132376710833355%7CUnknown%7CTWFpbGZsb3d8eyJFbXB0eU1hcGkiOnRydWUsIlYiOiIwLjAuMDAwMCIsIlAiOiJXaW4zMiIsIkFOIjoiTWFpbCIsIldUIjoyfQ%3D%3D%7C0%7C%7C%7C&sdata=QinABnPKVqSVo6Z2sIx3ePRBeojbIhoKZUavetjgkrs%3D&reserved=0>) and include the completed checklist as Supporting Information. Please add the following statement, or similar, to the Methods: "This study is reported as per the Guideline for Reporting Mediation Analyses (AGReMA) statement (S1 Checklist)." When completing the checklist, please use section and paragraph numbers, rather than page numbers.

**Reply:** Not applicable.

10. For all observational studies, in the manuscript text, please indicate: (1) the specific hypotheses you intended to test, (2) the analytical methods by which you planned to test them, (3) the analyses you actually performed, and (4) when reported analyses differ from those that were planned, transparent explanations for differences that affect the reliability of the study's results. If a reported analysis was performed based on an interesting but unanticipated pattern in the data, please be clear that the analysis was data driven.

**Reply:** We have included the relevant hypotheses in our Introduction (lines 98-104), delineate our analyses in our Methods section (lines 136-169), and report our findings in the Results section.

As for Point (4 – above, and next) there was no printed-up plan prior to the work being done. (Full reply next.)

11. Please state in the Methods section whether the study had a prospective protocol or analysis plan. If a prospective analysis plan (from your funding proposal, IRB or other ethics committee submission, study protocol, or other planning document written before analyzing the data) was used in designing the study, please include the relevant document(s) with your revised manuscript as a Supporting Information file to be published alongside your study and cite it in the Methods section. A legend for this file should be included at the end of your manuscript. If no such document exists, please make sure that the Methods section transparently describes when analyses were planned, and when/why any data-driven changes to analyses took place. Changes in the analysis, including those made in response to peer review comments, should be identified as such in the Methods section of the paper, with rationale.

**REPLY:** This is now laid out in the Analysis section, where we have made clear that this paper arose from prior work on the FI-Lab (reaching back to the 2014 paper which I introduced with Susan Howlett as the lead author, based on her preclinical work to introduce a mouse FI – by way of disclosure, Susan and I have been married for 41 years). We have also cited the two other papers with Hugh and me (the first being in and then one I did with Peter (and a host of co-authors, in PLOS MED last year; I moved it up to there). The FIs there were mostly made up of lab-based items.
