# Supplementary material for: Evaluation of the FI-Lab, a laboratory-based, automated frailty index for acute care: A multicohort study
Source: PLoS Med. 2026 Sep 1;23(9):e1005004. doi: 10.1371/journal.pmed.1005004 (PMC13577487; doi:10.1371/journal.pmed.1005004)
Supplement: Supplementary file 7 [file pmed.1005004.s004.docx]

PMEDICINE D-26-00743 - REPLY TO REVIEWERS

Comments from the reviewers:

Reviewer #1: *No further concern from my side*.

REPLY: Thank you.

Reviewer #2: *Thank you for responding to my comments. The revised paper is much clearer and accurate.*

REPLY: You’re welcome, and thank you.

Reviewer #3: I appreciate the thoughtful responses to my review.

REPLY: Thank you.

*I am left wondering, however, if the field would be best served by a different name for this instrument? FI-Lab invokes the cumulative deficit approach, whether the authors intend this or not, given how synonymous "FI" has become with eFI/Rockwood FI/cumulative deficit index etc.*

*I would also counter on the comparison to eFI - while the UK eFI is specific to outpatient there are many other electronic frailty indices that could be used as a comparison. As the US moves towards automated screening in hospitals as part of the CMS Age Friendly Health System measures, using the Rockwood FI approach, such a comparison could be valuable.*

REPLY: This is Ken Rockwood writing. I hear your comment about a name change. And “young frailty” is a newish concept. Still, it is an area of active inquiry by many groups. We have published a cross-sectional across-the-life-course FI-Lab under that name (ref 16). In Revision 2 though, we did not spend enough time on elaborating the FI-Lab as an FI. Saying that, I feel the need first to clarify the relationship between acute illness in younger people and frailty in older people. As we know, frailty is age-related; uncommon in younger people, very common by the eighth decade. Further, across the life course, underlying frailty can be unmasked by acute illness, even in young people at risk (e.g., from social vulnerability, congenital disorders, childhood cancer and its treatment, substance use disorders and its many complications). Here, I will be writing for this small audience; as you will see, the remedies by which we propose to address some unnecessary confusion in the R2 draft are much shorter in the R3 draft than what I write next. You can just cut to the final paragraph of this reply (the “Against this background” paragraph) without loss of meaning, but if you can manage it, please bear with me to understand the background. My goal here is to honour your questions and those of Reviewer 4, who objects to one part of our earlier reply to you, with a fuller answer.

By our team’s account, worked out over 25+ years, frailty arises from the accumulation of health deficits. This view, new to us at the turn of the century, over-turned our own much-too-contingent frailty definition that we’d used in the Canadian Study of Health & Aging (that definition we’d even managed to get published in the Lancet, in 1999^[1]^. The 2001 deficit accumulation FI we published in a then-novel e-journal^[2]^.)

The then-new frailty definition had two aims: simplicity, and mathematical tractability. Multiple dimensions could be reduced to a single number, based on a simple idea: count deficits. Self-consciously, it allowed us to embrace Vaupel’s statistical definition of frailty as variability in the risk of death for people of the same age.^[3]^ (The term was quickly generalized to be variability in the risk of an adverse outcome for people with the same degree of exposure.)

My colleagues (here chiefly Hugh Logan Ellis and Peter Hanlon who have done the analyses) and I see your question in these parts:

A.	*“FI-Lab invokes the cumulative deficit approach, whether the authors intend this or not…”*

REPLY: We do. The FI-Lab fits with essential deficit accumulation Frailty Index properties. Reviewer 4 had a similar concern, and I address this more below.

B. “*given how synonymous "FI" has become with eFI/Rockwood FI/cumulative deficit index etc.”*

REPLY: As we see it, your question now is whether the FI-lab is a frailty index. It is, as argued next. (Personally, I never use the “Rockwood FI” terminology; instead, it is an FI based on deficit accumulation – I resist somewhat snide “DAFI” abbreviation too.)

Note that already in the text (Line 121), we say “Building on the well-established deficit accumulation model of frailty[13], the laboratory-based frailty index (FI-Lab)[14] offers a different approach rooted in the mathematical principles describing complex systems at risk of collapse”. We have now changed this to read: Building on the well-established deficit accumulation model of frailty[13], the laboratory-based frailty index (FI-Lab)[14] offers **an approach that differs from prediction models and** is rooted in mathematical principles describing complex systems at risk of collapse. Further, we note in the Discussion that early work on the frailty index revealed properties such as initially higher scores at younger ages, maintenance of a limit, and normalization of the shape of the curve as age increases.

C*. I would also counter on the comparison to eFI - while the UK eFI is specific to outpatient there are many other electronic frailty indices that could be used as a comparison.*

REPLY: Agree. See how we tackle this point in reply to a suggestion from Reviewer 4.

D. *As the US moves towards automated screening in hospitals as part of the CMS Age Friendly Health System measures, using the Rockwood FI approach, such a comparison could be valuable.*

REPLY: I hope so. We agree that comparing FI-lab to other FIs is valuable and have done so below using the available measures in UK Biobank as presented in more detail in response to Reviewer 4. There we also show how, and as in ref 16, we can demonstrate that across the life course, an FI-Lab has the properties that we would expect of any FI. That contrasts with some of the “very quick” FIs being promoted; there is a lot of underbrush to clear out. Most EHRs appear to have little useful information about function, and I am afraid, based on what I’m sent to review, that frailty is being defined solely using diagnostic codes. That can be done, but outside the USVA, it appears that codes to indicate impaired function are little honoured, save as billings for supplying canes, wheelchairs and the like. The related issue is the (to me) unpardonable debasing of the FI by using as few diagnoses as possible – “the modified CSHA FI-11” being iteratively paired down to a modified CSHA FI-3. (Note, we have not burdened the paper with this.)

Reviewer #4: *I appreciate the authors' response and technical thoroughness. I continue to have conceptual issues with this work, however. While the authors, in their response to my comments, attempt to distance what they present from frailty, they plainly call this a "frailty index" and describe its relationship to the deficit accumulation model of frailty. The idea of capturing physiologic dysregulation through lab values without accounting for whether these values could, in an acute setting, be inappropriately normal further suggests that this bears a stronger relationship to frailty and the patient's baseline status than to acute physiologic dysregulation when receiving emergency care. My concern that this is not meaningfully different from frailty conceptually and in terms of its applications is supported by their response to reviewer 3: "However, we do not want to frame FI-lab as solely a frailty tool but as a latent-health-factor measure—an index of underlying physiological dysregulation, of which clinical frailty in older adults is one manifestation and acute illness severity is another." They go on to state "We envisage a broader use to measure some factor of general health status across ages and settings." On some level the lab-based frailty index presented is simply an attempt to go down a level or two of physiology from the usual construction of a frailty index (some of which, such as the Atrium Health eFI and the CGA-FI, do explicitly incorporate lab values*).

*I think that the best way to address this conceptual issue, to contextualize its association alongside established related measures, and in line with the authors' stated vision for this index, is to explicitly compare the lab-FI with other established measures of frailty that rely more on diagnosis codes, as suggested previously.*

A. REPLY: Thank you. We agree that, as originally worded in responding last time, we were not clear that the frailty index and measuring a latent construct of physiological dysregulation is more “yes/and” than “either/or”. At Line 131 we note that the theory of deficit accumulation is agnostic to age” to clarify our stance as “yes/and” not “either/or”, noting that “The proportion of abnormal biological parameters **captured in the FI-lab** is an index of dysregulation, **making it a latent** construct of overall health.” We do not doubt that patients with frailty can present with signs that are not typical for what is taught as textbook examples of illness, based on single-system disorders. Indeed, this is long a core part of teaching – an idea is especially “geriatric” in its nature. A common example is the patient with pneumonia whose white cell count and temperature go down and not up, who has a chronic cough and crackles that might or might not be new, whose normal blood pressure at 120mm systolic is 30 mm lower than normal, and whose absence of tachycardia reflects beta blockade complicating sinoatrial node dysfunction and so on. (On this I have dilated at some extent and over decades. Perhaps relevant here is that this information should not be kept secret, but propagated widely^[4]^.) In short, on the idea of atypical presentation, we are as one.

B. On the idea that an acutely ill older adult’s frailty would result in no profile of abnormal results, I am skeptical. In my experience, it is more often the case that, accompanying functional decline, or delirium, or impaired mobility they have laboratory abnormalities that are seen as “normal for their age” such as dysnatremias (for me, a hobby horse^[5]^ especially if seen with poor bed mobility^[6]^). In any case, we make clear in our Discussion that we are not proposing the FI-lab as the sole measure: “Finally, we position the FI-Lab not to replace clinical judgment, but as a data-driven frailty (or fitness) measure to help clinicians rapidly contextualize a patient's underlying health in light of their chronological age.”

C. Further regarding the conceptual issue, with the datasets to hand, we draw attention to the following, elaborated chiefly in the Discussion, to address this important point that you have raised. Firstly, using UK Biobank data to address the relationship with other frailty measures, we have evaluated the association of the FI-lab with mortality alongside two frailty measures: an earlier UK Biobank Frailty Index^[7]^ (which relies largely on diagnostic and symptom-based data) and Fried frailty phenotype as adapted in these same data.^[8]^ As you can see our results and in the supplemental tables, within each strata of both of these frailty measures, the FI-lab retains its association with mortality. Specifically, higher scores are associated with worse adverse outcomes, and show a dose response, for two separate approaches to frailty. Elaborating further on the point we made above (Reviewer 3, B) we have established that the FI-lab does indeed correlate with other frailty index measures, is multiply determined, and adds information in multivariable models in which other FIs are used.

Hazard ratios (95% CI) for mortality per SD-increase in FI-lab within strata of the Williams et al frailty index at baseline

| Frailty index at baseline | HR 1 year mortality (95% CI) | HR 10-year mortality (95% CI) |
| --- | --- | --- |
| 0 to 0.1 | 1.67 (1.53-1.83) | 1.30 (1.27-1.32) |
| >0.1 to 0.2 | 1.82 (1.72-1.92) | 1.39 (1.37-1.41) |
| >0.2 to 0.3 | 1.85 (1.71-1.99) | 1.41 (1.38-1.44) |
| >0.3 to 0.4 | 1.78 (1.52-2.08) | 1.37 (1.31-1.43) |
| >0.4 | 2.36 (1.33-4.18) | 1.54 (1.34-1.76) |

Hazard ratios (95% CI) for mortality per SD-increase in FI-lab across baseline levels of the adapted Frailty Phenotype

| Adapted frailty phenotype at baseline | HR 1 year mortality (95% CI) per SD increase in FI-lab | HR 10-year mortality (95% CI)  per SD increase in FI-lab |
| --- | --- | --- |
| Robust | 1.54 (1.41-1.69) | 1.27 (1.25-1.30) |
| Pre-frail | 1.73 (1.63-1.84) | 1.39 (1.37-1.41) |
| Frail | 1.81 (1.68-1.94) | 1.40 (1.37-1.44) |

*All models Cox-PH models adjusted for age and sex. FI-lab is based on 25 features, floor = 15.*

Secondly, as above, we underpin the idea that the measure can both be a frailty index and capture the latent variable of physiological reserve. Though not included in the paper due to space limitations, some years ago, we formally attempted this in collaboration with Prof Tom Kirkwood, an early adopter in measuring aging biomarkers^[9]^. Whereas in the Newcastle 85+ Study (n= 845) they had addressed the effects of the biomarkers individually, we combined them (as an FI-biomarkers; FI-B)^[10]^. We showed that the mean FI-B was 0.35 (SD, 0.08), higher than the mean FI-Clinical Deficits (0.22 +/-0.12). Further, at age 85, no one had an FI-B score <0.12. This we interpreted as the biomarker abnormality preceding the clinical deficits. In the Canadian Longitudinal Study of Health and Aging (CLSA; ages 45-85 years at baseline) self-report FI scores were lowest, then lab test abnormalities, and highest an FI based on the clinical examination. The last included measures of cognition, mobility and balance, bone density, anthropometrics, body composition, and spirometry^[11]^. Of the 30,027 people with Self-report FIs, 24,780 had data for all three FIs. Each FI showed the same characteristic features. On average, scores increased with age, with an upward increase in the mean FI. At any age, FI scores varied. Higher scores were accompanied by greater mortality risk. In each FI, the upper limit fell well short of 1.00 (0.47 for self-report, and 0.70 for lab tests and clinical examination). These features, common to FIs of varying composition, are shared by the FI-lab. We note too that using biomarkers to predict frailty is coming into its own^[12]^.

Thirdly, returning to data in the paper, in addressing this point in the Discussion, we now draw the readers’ attention to Figure 1 (FI-Lab Score Distributions Across Age Groups, Feature Number, and Feature Floor in MIMIC). As can be seen, most especially in the panel that has a minimum feature floor of 15, as age increases, the distribution of the scores broadens, even as a submaximal limit is maintained. These too are characteristic features of an FI^[13]^.

Fourthly, our use of an FI-lab in younger people is not without precedent. In addition, and in the CLSA paper above, an early report saw the FI estimated in people in the Canadian National Population Health Survey, as young as 15 years^[14]^. Thereby encouraged, we used US NHANES data to construct the 32-item FI-lab in the 2017 report noted above.^[15]^ It shows (in the Figure below) much the same pattern as here: fewer people have FI lab scores of 0, it increases with age and cellular events and appears to precede the development of self-reported health deficits. The notion of molecular and cellular events, themselves related to the degree of frailty, scaling up to have functional consequences has also been demonstrated in animal models^[16]^. This we now expand on as well, in both the Introduction and in the Discussion.

Against that background, in the paper under review we set out to extend the FI-lab approach in two ways. The first was to test it across the life course, focusing on its use in acutely ill patients. The second was to distinguish between acute and chronic changes. In this, we were motivated by experience in acute medicine. We are aware that ICUs have long used sometimes complicated illness severity measures. Our approach is much simpler: use data at hand; don’t bother with a complex weighting formula, and consider its use in acute illness, not just critical illness. With its simple, but still useful data configuration, we see the tool as being a useful addition in acute care, especially as people age. In short, it appears to work with acute accumulation of potentially reversible deficits as well. To be clear, our claim is not that everyone with a high FI-acute score is frail, but as above we recognize that acute illness can often unmask frailty. Finally, we draw attention to the fact that when it does unmask it, some people will be younger than where much FI discussion lies presently.

1. Rockwood K, Stadnyk K, MacKnight C, et al., A brief clinical instrument to classify frailty in elderly people. Lancet. 1999 Jan 16;353(9148):205-6. doi: 10.1016/S0140-6736(98)04402-X.
2. Mitnitski AB, Mogilner AJ, Rockwood K. Accumulation of deficits as a proxy measure of aging. ScientificWorldJournal. 2001 Aug 8;1:323-36. doi: 10.1100/tsw.2001.58.
3. Vaupel JW, Manton KG, Stallard E. The impact of heterogeneity in individual frailty on the dynamics of mortality. Demography. 1979 Aug;16(3):439-54.
4. Rockwood K. Medical management of frailty: confessions of a gnostic. CMAJ. 1997;157(8):1081-4
5. Miller AJ, Theou O, McMillan M, et al., . Dysnatremia in Relation to Frailty and Age in Community-dwelling Adults in the National Health and Nutrition Examination Survey. J Gerontol A Biol Sci Med Sci. 2017 Mar 1;72(3):376-381. doi: 10.1093/gerona/glw114. George J, Rockwood K. Dehydration and delirium--not a simple relationship. J Gerontol A Biol Sci Med Sci. 2004 Aug;59(8):811-2. doi: 10.1093/gerona/59.8.m811.
6. Hatheway OL, Mitnitski A, Rockwood K. Frailty affects the initial treatment response and time to recovery of mobility in acutely ill older adults admitted to hospital. Age Ageing. 2017;46(6):920-925. doi: 10.1093/ageing/afw257..
7. Williams DM, Jylhävä J, Pedersen NL, Hägg S. A Frailty Index for UK Biobank Participants. J Gerontol A Biol Sci Med Sci. 2019 Mar 14;74(4):582-587. doi: 10.1093/gerona/gly094
8. [Hanlon P, Nicholl BI, Jani BD, Lee D, McQueenie R, Mair FS. Frailty and pre-frailty in middle-aged and older adults and its association with multimorbidity and mortality: a prospective analysis of 493 737 UK Biobank participants. Lancet Public Health. 2018 Jul;3(7):e323-e332. doi: 10.1016/S2468-2667(18)30091-4.]
9. Collerton J, Martin-Ruiz C, Davies K, Hilkens CM, Isaacs J, Kolenda C, et al. Frailty and the role of inflammation, immunosenescence and cellular ageing in the very old: cross-sectional findings from the Newcastle 85+ Study. Mech Ageing Dev. 2012;133:456–66
10. Mitnitski A, Collerton J, Martin-Ruiz C,et al., Age-related frailty and its association with biological markers of ageing. BMC Med. 2015 Jul 13;13:161. doi: 10.1186/s12916-015-0400-x.
11. Blodgett JM, Pérez-Zepeda MU, Godin J, et L., Frailty indices based on self-report, blood-based biomarkers and examination-based data in the Canadian Longitudinal Study on Aging. Age Ageing. 2022;51(5):afac075. doi: 10.1093/ageing/afac075.
12. Garst S, Kuiper L, van den Akker E, van den Berg N, Ghanbari M, Mooijaart S, Beekman M, Reinders M, Slagboom PE, van Meurs J. ProtFI, an efficient frailty-trained proteomics-based biomarker of aging, robustly predicts age-related decline. Cell Rep Methods. 2026 May 18;6(5):101405. doi: 10.1016/j.crmeth.2026.101405.
13. Rockwood K, Mitnitski A. Limits to deficit accumulation in elderly people. Mech Ageing Dev. 2006 May;127(5):494-6. doi: 10.1016/j.mad.2006.01.002. Rutenberg AD, Mitnitski AB, Farrell SG, Rockwood K. Unifying aging and frailty through complex dynamical networks. Exp Gerontol. 2018 Jul 1;107:126-129. doi: 10.1016/j.exger.2017.08.027.
14. Rockwood, K ∙ Song, X ∙ Mitnitski, A Changes in relative fitness and frailty across the adult lifespan: evidence from the Canadian National Population Health Survey CMAJ. 2011; 183:E487-E49
15. Smeeth D, Warren-Gash C, Green RE,et al., Associations of common infections with frailty and mortality in two UK cohort studies. J Gerontol A Biol Sci Med Sci. 2026 Mar 10;81(4):glag046. doi: 10.1093/gerona/glag046.
16. Howlett SE, Rutenberg AD, Rockwood K. The degree of frailty as a translational measure of health in aging. Nat Aging. 2021 Aug;1(8):651-665. doi: 10.1038/s43587-021-00099-3. PMID: 37117769.
