# Supplementary material for: Evaluation of the FI-Lab, a laboratory-based, automated frailty index for acute care: A multicohort study
Source: PLoS Med. 2026 Sep 1;23(9):e1005004. doi: 10.1371/journal.pmed.1005004 (PMC13577487; doi:10.1371/journal.pmed.1005004)
Supplement: Peer review history

# Editor report: Decision Letter 0

**DOI:** 10.1371/journal.pmed.1005004.r001

**Authors:** Katarzyna Starowicz

4 Mar 2026

Dear Dr Rockwood,

Thank you for submitting your manuscript entitled "The FI-Lab: A Simple, Scalable, Electronic Health Measure of Frailty" for consideration by PLOS Medicine.

Your manuscript has now been evaluated by the PLOS Medicine editorial staff, and I am writing to let you know that we would like to send your submission out for external peer review.

However, before we can send your manuscript to reviewers, we need you to complete your submission by providing the metadata that is required for full assessment. To this end, please login to Editorial Manager where you will find the paper in the 'Submissions Needing Revisions' folder on your homepage. Please click 'Revise Submission' from the Action Links and complete all additional questions in the submission questionnaire.

For clinical studies, please upload a copy of your trial study protocol as a supporting information file. The study protocol should be the version submitted for approval to the institutional review board or ethics committee, should include any amendments to the study protocol, as well as the date of their approval by the institutional review or ethics committee. Please also detail any deviations from the study protocol in the Methods section of your manuscript. The editors will consider the protocol and study conduct prior to a final decision for external review.

Please re-submit your manuscript within two working days, i.e. by Mar 06 2026 11:59PM.

Login to Editorial Manager here: https://www.editorialmanager.com/pmedicine

Once your full submission is complete, your paper will undergo a series of checks in preparation for peer review. Once your manuscript has passed all checks it will be sent out for review.

Feel free to email us at plosmedicine@plos.org if you have any queries relating to your submission.

Kind regards,

Katarzyna Starowicz, PhD

Associate Editor

PLOS Medicine

kstarowicz@plos.org

# Peer review: Decision Letter 1

**DOI:** 10.1371/journal.pmed.1005004.r002

**Authors:** Katarzyna Starowicz

9 Apr 2026

Dear Dr Rockwood,

Many thanks for submitting your manuscript "The FI-Lab: A Simple, Scalable, Electronic Health Measure of Frailty" (PMEDICINE-D-26-00743R1) to PLOS Medicine. The paper has been reviewed by subject experts and a statistician; their comments are included below and can also be accessed here: [LINK]

After discussing the paper with the editorial team and an academic editor with relevant expertise, I'm pleased to invite you to revise the paper in response to the reviewers' comments. As you will see, the reviewers find your study important for the field however, they have some concerns which you need to address in full. We plan to send the revised paper to some or all of the original reviewers, and we cannot provide any guarantees at this stage regarding publication.

In addition to these revisions, you may need to complete some formatting changes, which you will receive in a follow up email. A member of our team will be in touch with a set of requests shortly. If you do not receive a separate email within a few days, please assume that checks have been completed, and no additional changes are required.

When you upload your revision, please include a point-by-point response that addresses all of the reviewer and editorial points, indicating the changes made in the manuscript and either an excerpt of the revised text or the location (eg: page and line number) where each change can be found. Please also be sure to check the general editorial comments at the end of this letter and include these in your point-by-point response. When you resubmit your paper, please include a clean version of the paper as the main article file and a version with changes tracked as a marked-up manuscript. It may also be helpful to check the guidelines for revised papers at http://journals.plos.org/plosmedicine/s/revising-your-manuscript for any that apply to your paper.

We ask that you submit your revision by Apr 30 2026 11:59PM. However, if this deadline is not feasible, please contact me by email, and we can discuss a suitable alternative.

Don't hesitate to contact me directly with any questions (kstarowicz@plos.org).

Best regards,

Katarzyna

Katarzyna Starowicz, PhD

Associate Editor

PLOS Medicine

kstarowicz@plos.org

-----------------------------------------------------------

Comments from the reviewers:

Reviewer #1: 1. Using standard Cox models for readmission outcomes ignores the strong competing risk of high 1-year mortality (e.g., 7.3% in MIMIC-IV-ED), which likely overestimates readmission risks. Despite citing "computational constraints," the authors should employ Fine-Gray or cause-specific hazard models (at least in sensitivity analyses) for methodological rigor

2. FI-Lab relies on standard laboratory reference ranges, which are typically for healthy adults and lack age stratification. The authors should discuss how this binary thresholding might misclassify normal age-related physiological changes as "deficits" and affect construct validity in older adults.

3. ED lab tests are ordered with high clinical suspicion. The authors should further discuss whether FI-Lab thresholds validated in this high-pretest-probability environment might overestimate its predictive performance when applied to routine community screening (e.g., UK Biobank).

4. The claim that FI-Lab is robust across diverse settings is overstated. The cohorts only represent high-income countries (US and UK) with mature EHR systems. Its applicability in resource-constrained environments lacks data support.

Minor: the finding that predictive discrimination plateaus at 20-40 features is highly valuable. This specific numerical recommendation should be explicitly added to the Abstract's conclusion to improve clinical utility.

Reviewer #2: This is a retrospective multicohort study that explored the optimal configuration of the Laboratory-based frailty indices (FI-Lab) in two emergency (MIMIC-IV-ED; KCH) and one community (UK Biobank) setting. This is an interesting study and the methods used appear largely robust. I have several comments:

1. Several exclusion criteria were applied (lines 107-110). Please specify the number excluded for each criteria.

2. The 'Important' tests were determined based on a random forest model but no details were provided. How was it defined based on the model? what was the underlying data? if using the same data for evaluation, would it be a cause for overfitting?

3. Feature floor - presumably this will affect the numbers of patients included in analysis. Please specify this and whether the analyses comparing different feature floors were based on the common set of patients. If the sample size differs, the comparison won't be fair; otherwise there'd be a selection bias skewed towards the sicker patients.

4. Table 2 is a good summary of the findings, though I'm not sure I agree that increasing feature floor 'generally improves' performance. with a few exceptions, the differences in C indices appeared quite small.

5. I also am not sure I fully understand Figure 3. Why the age panels have number of features as x-axis? And the comparison with age would dependent on how age is parametrerised. If age is expressed as per 5 years increase the HR would be greater, for example.

6. One notable limitation i think is that each dataset would have own 'normal' definition, which made them not comparable. This should at least be acknowledged. Additionally the setting are also not comparable between A&E and UK Biobank, which could explain the lower performance in UKB.

Reviewer #3: The authors seek to develop a Lab-FI for use in a clinical setting from commonly available biomarkers. This is an important and necessary step in the field of frailty medicine and may have real meaningful outcome for helping to triage patients at the point of care.

The manuscript is well written and the authors are commended for the extensive analyses done to validate the Lab-FI, which are quite sound. I have a few fundamental considerations for the authors to consider.

1.Given existing Lab-FI data from other cohorts, strong rationale is needed for a new framework, particularly one that appears to be validated on mortality specifically (eg Methods line 125 "'Important' tests for mortality) when frailty is distinctly a syndrome of vulnerability that predicts mortality but is not defined by it. Better clarification for how the Lab-FI components were selected is needed to frame this as a frailty tool. I suggest an anchor on the original FI-Lab developed by Howlett, Rockwood et al. Otherwise, this becomes yet another FI that may or may not be useful.

2.Moreover, comparison to NEWS2 makes this a very UK specific analysis. It would strengthen the results considerably to compare the Lab-FI to an eFI which is more broadly used for frailty assessment (vs mortality risk, as noted above).

3.Given the text in the manuscript, I was surprised to see that the Lab-FI includes biometric measures such as BP and HR which vary at times minute to minute. Additionally, inclusion of potentially toxic exposures (ethanol, acetaminophen) will be rarely measured outside of the emergency department making these less generalizable. Also, as above, how are these measures of frailty?

Reviewer #4: The authors present a thorough manuscript testing approaches to developing a laboratory marker-based frailty index using EHR data. This approach complements other approaches to assessing frailty in EHR data using diagnoses, procedures, and prescription data. The authors' work carefully explores the hyper-parameters of such an index using data from different EHR systems in two different countries and with different clinical contexts. On the whole this is a well-done study. There are a few points the authors may wish to clarify.

The first point is conceptual and should be at least mentioned in the discussion, though there is no clear resolution. Frailty is meant to capture reduced ability to respond to physiologic stressors. This suggests that values for acute phase reactants, such as WBC or lactate, might be inappropriately normal in acute illness in frail people. By relying largely on values from ED/A&E visits, this index may represent severity of acute illness rather than frailty. This may explain some of the differences in discrimination among the UK Biobank, KCH, and MIMIC-IV. This might also explain some of the decline in AUC with additional labs in MIMIC-IV.

Second, the details of how frailty was measured and used in the Cox regressions are unclear. The makeup of the 25-items FI used in the Cox regressions should be clearly presented in the appendix. The scale should also be made clear: an FI is typically a proportion between 0 and 1, however Figure 1 suggests it was measured on a percentage point scale. Cox regressions yielded HRs of ~2, a very strong relationship on the percentage point scale, but on the 0-1 scale implies an HR of 1.07 for a 0.1-increment change in FI, similar to the NEWS score. Finally, the FI distribution is a non-normal and asymmetric; bootstrapped confidence intervals may be more appropriate than analytic ones.

Third and finally, this is a paper investigating a different approach to assessing frailty and it is worth including a comparison with a more traditional FI and possibly with physical frailty. This is likely most feasible in the UK Biobank data. Both FIs and physical frailty have been developed in this cohort (see Williams DM et al. J Gerontol A Biol Sci Med Sci. 2019;74(4):582-587. and Hanlon P et al. Lancet Public Health. 2018;3(7):e323-e332.).

Minor Comments:

Figure S2 is not referenced in the manuscript and looks identical to Figure 1. It should be removed.

Any attachments provided with reviews can be seen via the following link: [LINK]

--------------------------------------------------------- ---

General editorial requests:

(Note: not all will apply to your paper, but please check each item carefully)

*We ask every co-author listed on the manuscript to fill in a contributing author statement, making sure to declare all competing interests. If any of the co-authors have not filled in the statement, we will remind them to do so when the paper is revised. If all statements are not completed in a timely fashion this could hold up the re-review process. If new competing interests are declared later in the revision process, this may also hold up the submission. Should there be a problem getting one of your co-authors to fill in a statement we will be in contact. Please do not add or remove authors without first discussing this with the handling editor. You can see our competing interests policy here: http://journals.plos.org/plosmedicine/s/competing-interests.

*Please upload any figures associated with your paper as individual TIF or EPS files with 300dpi resolution at resubmission; please read our figure guidelines for more information on our requirements: http://journals.plos.org/plosmedicine/s/figures. While revising your submission, we strongly recommend that you use PLOS's NAAS tool (https://ngplosjournals.pagemajik.ai/artanalysis) to test your figure files. NAAS can convert your figure files to the TIFF file type and meet basic requirements (such as print size, resolution), or provide you with a report on issues that do not meet our requirements and that NAAS cannot fix.

After uploading your figures to PLOS's NAAS tool - https://ngplosjournals.pagemajik.ai/artanalysis, NAAS will process the files provided and display the results in the "Uploaded Files" section of the page as the processing is complete.

If the uploaded figures meet our requirements (or NAAS is able to fix the files to meet our requirements), the figure will be marked as "fixed" above. If NAAS is unable to fix the files, a red "failed" label will appear above.

When NAAS has confirmed that the figure files meet our requirements, please download the file via the download option, and include these NAAS processed figure files when submitting your revised manuscript.

*Please ensure that the paper adheres to the PLOS Data Availability Policy (see http://journals.plos.org/plosmedicine/s/data-availability), which requires that all data underlying the study's findings be provided in a repository or as Supporting Information. For data residing with a third party, authors are required to provide instructions with contact information (web or email address) for obtaining the data. Please note that a study author cannot be the contact person for the data. PLOS journals do not allow statements supported by "data not shown" or "unpublished results." For such statements, authors must provide supporting data or cite public sources that include it.

*We expect all researchers with submissions to PLOS in which author-generated code underpins the findings in the manuscript to make all author-generated code available without restrictions upon publication of the work. In cases where code is central to the manuscript, we may require the code to be made available as a condition of publication. Authors are responsible for ensuring that the code is reusable and well documented. Please make any custom code available, either as part of your data deposition or as a supplementary file. Please add a sentence to your data availability statement regarding any code used in the study, e.g. "The code used in the analysis is available from Github [URL] and archived in Zenodo [DOI link]" Please review our guidelines at https://journals.plos.org/plosmedicine/s/materials-software-and-code-sharing and ensure that your code is shared in a way that follows best practice and facilitates reproducibility and reuse. Because Github depositions can be readily changed or deleted, we encourage you to make a permanent DOI'd copy (e.g. in Zenodo) and provide the URL.

*Please ensure that the study is reported according to the relevant guideline and include the completed [XXXX] checklist as Supporting Information. When completing the checklist, please use section and paragraph numbers, rather than page numbers. Please add the following statement, or similar, to the Methods: "This study is reported as per [XXXX] guideline (S1 Checklist)."

FORMATTING - GENERAL

*Abstract: Please structure your abstract using the PLOS Medicine headings (Background, Methods and Findings, Conclusions). Please combine the Methods and Findings sections into one section.

*At this stage, we ask that you include a short, non-technical Author Summary of your research to make findings accessible to a wide audience that includes both scientists and non-scientists. The Author Summary should immediately follow the Abstract in your revised manuscript. This text is subject to editorial change and should be distinct from the scientific abstract. Ideally each sub-heading should contain 2-3 single sentence, concise bullet points containing the most salient points from your study. In the final bullet point of 'What Do These Findings Mean?', please include the main limitations of the study in non-technical language. Please see our author guidelines for more information: https://journals.plos.org/plosmedicine/s/revising-your-manuscript#loc-author-summary.

*Please express the main results with 95% CIs as well as p values. When reporting p values please report as p<0.001 and where higher as the exact p value p=0.002, for example. Throughout, suggest reporting statistical information as follows to improve clarity for the reader "22% (95% CI [13%,28%]; p</=)". Please be sure to define all numerical values at first use.

*Please include page numbers and line numbers in the manuscript file. Use continuous line numbers (do not restart the numbering on each page).

*Please cite the reference numbers in square brackets. Citations should precede punctuation.

FIGURES AND TABLES

*Please provide titles and legends for all figures and tables (including those in Supporting Information files).

*Please define all abbreviations used in each figure/table (including those in Supporting Information files).

*Please consider avoiding the use of red and green in order to make your figure more accessible to those with color blindness.

SUPPLEMENTARY MATERIAL

*Please note that supplementary material will be posted as supplied by the authors. Therefore, please amend it according to the relevant comments outlined here.

*Please cite your Supporting Information as outlined here: https://journals.plos.org/plosmedicine/s/supporting-information

REFERENCES

*PLOS uses the numbered citation (citation-sequence) method and first six authors, et al.

*Please ensure that journal name abbreviations match those found in the National Center for Biotechnology Information (NCBI) databases (http://www.ncbi.nlm.nih.gov/nlmcatalog/journals), and are appropriately formatted and capitalised.

*Where website addresses are cited, please include the complete URL and specify the date of access (e.g. [accessed: 12/06/2023]).

*Please also see https://journals.plos.org/plosmedicine/s/submission-guidelines#loc-references for further details on reference formatting.

STUDY TYPE-SPECIFIC REQUESTS - please note not all might apply:

OBSERVATIONAL STUDIES

*Abstract: Please include the study design, population and setting, number of participants, years during which the study took place (enrollment and follow up), length of follow up, and main outcome measures.

*Please ensure that the study is reported according to the STROBE (or appropriate STOBE extension) guideline (available from: https://www.equator-network.org/reporting-guidelines/strobe) and include the completed STROBE (or STROBE extension) checklist as Supporting Information. Please add the following statement, or similar, to the Methods: "This study is reported as per the Strengthening the Reporting of Observational Studies in Epidemiology (STROBE) guideline (S1 Checklist)." When completing the checklist, please use section and paragraph numbers, rather than page numbers.

*[FOR POPULATION HEALTH/REGISTRY STUDIES] Please ensure that the study is reported according to the RECORD guideline (available from https://www.record-statement.org) and include the completed checklist as Supporting Information. Please add the following statement, or similar, to the Methods: "This study is reported as per the Reporting of Studies Conducted using Observational Routinely-Collected Data (RECORD) guideline (S1 Checklist)." When completing the checklist, please use section and paragraph numbers, rather than page numbers.

*[FOR POPULATION HEALTH ESTIMATES] Please ensure that the study is reported according to the GATHER statement (available from https://www.equator-network.org/reporting-guidelines/gather-statement) and include the completed checklist as Supporting Information. Please add the following statement, or similar, to the Methods: "This study is reported as per the Guidelines for Accurate and Transparent Health Estimates Reporting (GATHER) statement (S1 Checklist)." When completing the checklist, please use section and paragraph numbers, rather than page numbers.

*[FOR MEDIATION ANALYSES] We recommend that the study is reported according to the AGReMA statement (https://agrema-statement.org/#:~:text=AGReMA%20is%20an%20evidence%2D%20and,randomised%20trials%20and%20observational%20studies) and include the completed checklist as Supporting Information. Please add the following statement, or similar, to the Methods: "This study is reported as per the Guideline for Reporting Mediation Analyses (AGReMA) statement (S1 Checklist)." When completing the checklist, please use section and paragraph numbers, rather than page numbers.

*For all observational studies, in the manuscript text, please indicate: (1) the specific hypotheses you intended to test, (2) the analytical methods by which you planned to test them, (3) the analyses you actually performed, and (4) when reported analyses differ from those that were planned, transparent explanations for differences that affect the reliability of the study's results. If a reported analysis was performed based on an interesting but unanticipated pattern in the data, please be clear that the analysis was data driven.

*Please state in the Methods section whether the study had a prospective protocol or analysis plan. If a prospective analysis plan (from your funding proposal, IRB or other ethics committee submission, study protocol, or other planning document written before analyzing the data) was used in designing the study, please include the relevant document(s) with your revised manuscript as a Supporting Information file to be published alongside your study and cite it in the Methods section. A legend for this file should be included at the end of your manuscript. If no such document exists, please make sure that the Methods section transparently describes when analyses were planned, and when/why any data-driven changes to analyses took place. Changes in the analysis, including those made in response to peer review comments, should be identified as such in the Methods section of the paper, with rationale.

MODELLING STUDIES

The following list is derived from Geoffrey P Garnett, Simon Cousens, Timothy B Hallett, Richard Steketee, Neff Walker. Mathematical models in the evaluation of health programmes. (2011) Lancet DOI:10.1016/S0140-6736(10)61505-X:

*If pertinent, please provide a diagram that shows the model structure, including how the natural history of the disease is represented, the process and determinants of disease acquisition, and how the putative intervention could affect the system.

*Please provide a complete list of model parameters, including clear and precise descriptions of the meaning of each parameter, together with the values or ranges for each, with justification or the primary source cited and important caveats about the use of these values noted.

*Please provide a clear statement about how the model was fitted to the data, including goodness-of-fit measure, the numerical algorithm used, which parameter varied, constraints imposed on parameter values, and starting conditions.

*For uncertainty analyses, please state the sources of uncertainties quantified and not quantified [can include parameter, data, and model structure].

*Please provide sensitivity analyses to identify which parameter values are most important in the model. Uncertainty estimates seek to derive a range of credible results on the basis of an exploration of the range of reasonable parameter values. The choice of method should be presented and justified.

*Please discuss the scientific rationale for the choice of model structure and identify points where this choice could influence conclusions drawn. Please also describe the strength of the scientific basis underlying the key model assumptions.

*For studies that develop a prediction model or evaluate its performance, please ensure that the study is reported according to the TRIPOD statement (https://www.equator-network.org/reporting-guidelines/tripod-statement) and include the completed checklist as Supporting Information. Please add the following statement, or similar, to the Methods: "This study is reported as per the Transparent Reporting of a Multivariable Prediction Model for Individual Prognosis Or Diagnosis (TRIPOD) statement (S1 Checklist)." For studies using machine learning, please use the TRIPOD-AI checklist. When completing the checklist, please use section and paragraph numbers, rather than page numbers.

# Author comment: Author response to Decision Letter 2

**DOI:** 10.1371/journal.pmed.1005004.r003

30 Apr 2026

Attachment Submitted filename: Response_to_Reviewers_PMEDICINE-D-26-00743_R2_May4-.docx

# Peer review: Decision Letter 2

**DOI:** 10.1371/journal.pmed.1005004.r004

**Authors:** Katarzyna Starowicz

2 Jun 2026

Dear Dr Rockwood,

Many thanks for submitting your manuscript "The FI-Lab: A Simple, Scalable, Electronic Health Measure of Frailty" (PMEDICINE-D-26-00743R2) to PLOS Medicine. The paper has been reviewed by subject experts and a statistician; their comments are included below and can also be accessed here: [LINK]

Your manuscript has been see by all original reviewers. As you will see, the reviewers #3 and #4 feel that their concerns were not fully addressed. After discussing the paper with the editorial team and an academic editor with relevant expertise, I'm pleased to invite you to revise the paper in response to the reviewers' comments. We think that they have provided valuable feedback and we would like you to address their concerns in full.

We plan to send the revised paper to some or all of the original reviewers, and we cannot provide any guarantees at this stage regarding publication.

In addition to these revisions, you may need to complete some formatting changes, which you will receive in a follow up email. A member of our team will be in touch with a set of requests shortly. If you do not receive a separate email within a few days, please assume that checks have been completed, and no additional changes are required.

When you upload your revision, please include a point-by-point response that addresses all of the reviewer and editorial points, indicating the changes made in the manuscript and either an excerpt of the revised text or the location (eg: page and line number) where each change can be found. Please also be sure to check the general editorial comments at the end of this letter and include these in your point-by-point response. When you resubmit your paper, please include a clean version of the paper as the main article file and a version with changes tracked as a marked-up manuscript. It may also be helpful to check the guidelines for revised papers at http://journals.plos.org/plosmedicine/s/revising-your-manuscript for any that apply to your paper.

We ask that you submit your revision by Jun 16 2026 11:59PM. However, if this deadline is not feasible, please contact me by email, and we can discuss a suitable alternative.

Don't hesitate to contact me directly with any questions (kstarowicz@plos.org).

Best regards,

Katarzyna

Katarzyna Starowicz, PhD

Associate Editor

PLOS Medicine

kstarowicz@plos.org

-----------------------------------------------------------

Comments from the reviewers:

Reviewer #1: No further concern from my side.

Reviewer #2: Thank you for responding to my comments. The revised paper is much clearer and accurate.

Reviewer #3: I appreciate the thoughtful responses to my review.

I am left wondering, however, if the field would be best served by a different name for this instrument? FI-Lab invokes the cumulative deficit approach, whether the authors intend this or not, given how synonymous "FI" has become with eFI/Rockwood FI/cumulative deficit index etc.

I would also counter on the comparison to eFI - while the UK eFI is specific to outpatient there are many other electronic frailty indices that could be used as a comparison. As the US moves towards automated screening in hospitals as part of the CMS Age Friendly Health System measures, using the Rockwood FI approach, such a comparison could be valuable.

Reviewer #4: I appreciate the authors' response and technical thoroughness. I continue to have conceptual issues with this work, however. While the authors, in their response to my comments, attempt to distance what they present from frailty, they plainly call this a "frailty index" and describe its relationship to the deficit accumulation model of frailty. The idea of capturing physiologic dysregulation through lab values without accounting for whether these values could, in an acute setting, be inappropriately normal further suggests that this bears a stronger relationship to frailty and the patient's baseline status than to acute physiologic dysregulation when receiving emergency care. My concern that this is not meaningfully different from frailty conceptually and in terms of its applications is supported by their response to reviewer 3: "However, we do not want to frame FI-lab as solely a frailty tool but as a latent-health-factor measure—an index of underlying physiological dysregulation, of which clinical frailty in older adults is one manifestation and acute illness severity is another." They go on to state "We envisage a broader use to measure some factor of general health status across ages and settings." On some level the lab-based frailty index presented is simply an attempt to go down a level or two of physiology from the usual construction of a frailty index (some of which, such as the Atrium Health eFI and the CGA-FI, do explicitly incorporate lab values).

I think that the best way to address this conceptual issue, to contextualize its association alongside established related measures, and in line with the authors' stated vision for this index, is to explicitly compare the lab-FI with other established measures of frailty that rely more on diagnosis codes, as suggested previously.

Any attachments provided with reviews can be seen via the following link: [LINK]

--------------------------------------------------------- ---

# Author comment: Author response to Decision Letter 3

**DOI:** 10.1371/journal.pmed.1005004.r005

25 Jun 2026

Attachment Submitted filename: PMEDICINE D-26-00743_R3 REPLY TO REVIEWERS JUNE 22.docx

# Peer review: Decision Letter 3

**DOI:** 10.1371/journal.pmed.1005004.r006

**Authors:** Katarzyna Starowicz

16 Jul 2026

Dear Dr. Rockwood,

Thank you very much for re-submitting your manuscript "The FI-Lab: A Simple, Scalable, Electronic Health Measure of Frailty" (PMEDICINE-D-26-00743R3) for review by PLOS Medicine.

I have discussed the paper with my colleagues and the academic editor and it was also seen again by two reviewers. I am pleased to say that provided the remaining reviewers, editorial and production issues are dealt with we are planning to accept the paper for publication in the journal.

The remaining issues that need to be addressed are listed at the end of this email. As during previous revision please include your rebuttal letter to all editorial and reviewers comments.

Any accompanying reviewer attachments can be seen via the link below. Please take these into account before resubmitting your manuscript:

[LINK]

***Please note while forming your response, if your article is accepted, you may have the opportunity to make the peer review history publicly available. The record will include editor decision letters (with reviews) and your responses to reviewer comments. If eligible, we will contact you to opt in or out.***

In revising the manuscript for further consideration here, please ensure you address the specific points made by each reviewer and the editors. In your rebuttal letter you should indicate your response to the reviewers' and editors' comments and the changes you have made in the manuscript. Please submit a clean version of the paper as the main article file. A version with changes marked must also be uploaded as a marked up manuscript file.

Please also check the guidelines for revised papers at http://journals.plos.org/plosmedicine/s/revising-your-manuscript for any that apply to your paper. If you haven't already, we ask that you provide a short, non-technical Author Summary of your research to make findings accessible to a wide audience that includes both scientists and non-scientists. The Author Summary should immediately follow the Abstract in your revised manuscript. This text is subject to editorial change and should be distinct from the scientific abstract.

In addition to these revisions, you may need to complete some formatting changes, which you will receive in a follow up email. A member of our team will be in touch with a set of requests shortly. If you do not receive a separate email within a few days, please assume that checks have been completed, and no additional changes are required.

We expect to receive your revised manuscript within 1 week. Please email us (plosmedicine@plos.org) if you have any questions or concerns.

We ask every co-author listed on the manuscript to fill in a contributing author statement. If any of the co-authors have not filled in the statement, we will remind them to do so when the paper is revised. If all statements are not completed in a timely fashion this could hold up the re-review process. Should there be a problem getting one of your co-authors to fill in a statement we will be in contact. YOU MUST NOT ADD OR REMOVE AUTHORS UNLESS YOU HAVE ALERTED THE EDITOR HANDLING THE MANUSCRIPT TO THE CHANGE AND THEY SPECIFICALLY HAVE AGREED TO IT.

Please ensure that the paper adheres to the PLOS Data Availability Policy (see http://journals.plos.org/plosmedicine/s/data-availability), which requires that all data underlying the study's findings be provided in a repository or as Supporting Information. For data residing with a third party, authors are required to provide instructions with contact information for obtaining the data. PLOS journals do not allow statements supported by "data not shown" or "unpublished results." For such statements, authors must provide supporting data or cite public sources that include it.

To enhance the reproducibility of your results, we recommend that you deposit your laboratory protocols in protocols.io, where a protocol can be assigned its own identifier (DOI) such that it can be cited independently in the future. Additionally, PLOS ONE offers an option to publish peer-reviewed clinical study protocols. Read more information on sharing protocols at https://plos.org/protocols?utm_medium=editorial-email&utm_source=authorletters&utm_campaign=protocols

Please review your reference list to ensure that it is complete and correct. If you have cited papers that have been retracted, please include the rationale for doing so in the manuscript text, or remove these references and replace them with relevant current references. Any changes to the reference list should be mentioned in the rebuttal letter that accompanies your revised manuscript.

Please note, when your manuscript is accepted, an uncorrected proof of your manuscript will be published online ahead of the final version, unless you've already opted out via the online submission form. If, for any reason, you do not want an earlier version of your manuscript published online or are unsure if you have already indicated as such, please let the journal staff know immediately at plosmedicine@plos.org.

If you have any questions in the meantime, please contact me or the journal staff on plosmedicine@plos.org.

We look forward to receiving the revised manuscript by Jul 24 2026 11:59PM.

Sincerely,

Katarzyna Starowicz, PhD

Associate Editor, PLOS Medicine

kstarowicz@plos.org

------------------------------------------------------------

Requests from Editors:

GENERAL EDITORIAL REQUESTS

* Please confirm that your title complies with PLOS Medicine's style. Your title must be nondeclarative and not a question. It should begin with main concept if possible. "Effect of" should be used only if causality can be inferred, i.e., for an RCT. Please place the study design ("A randomized controlled trial," "A retrospective study," "A modelling study," etc.) in the subtitle (ie, after a colon).

e.g “Evaluation of FI-Lab, a laboratory-based, automated frailty index for acute care: a multicohort study” this is only an example and we are open for other suggestions.

* Please confirm that your abstract complies with our requirements, including format (three sections: Background, Methods and Findings, and Conclusions) and providing all the information relevant to this study type https://journals.plos.org/plosmedicine/s/submission-guidelines#loc-abstract

* Please ensure that the Introduction ends with a clear description of the study question or hypothesis.

* Please ensure that all abbreviations are defined at first use throughout the text.

* Please confirm that all numbers presented in the abstract are present and identical to numbers presented in the main manuscript text.

GENERAL

* Please disclose any use of AI tools and technologies to the study or article content at the end of the Methods. Please specify the name(s) and version(s) of any tool(s) used and a description of their use, including how the AI-assisted or generated content was evaluated by authors, and a clear statement of which aspects of the study, article contents, data, or supporting files were affected/generated by AI tool usage. Please also include a statement specifying who retains responsibility for the final manuscript. See our guidelines for more information: https://journals.plos.org/plosmedicine/s/ethical-publishing-practice#loc-artificial-intelligence-tools-and-technologies.

* Please review your text for claims of novelty or primacy (e.g. 'for the first time') and remove this language. In addition, please check that any use of statistical terms (such as trend or significant) are supported by the data, and if not please remove them.

* Please remove the 'conclusions' subheading from the discussion. Please also remove any other subheadings from the discussion.

* Statistical reporting: Please revise throughout the manuscript, including tables and figures.

- Please report statistical information as follows to improve clarity for the reader ""22% (95% CI [13,28]; p</=)"".

- Please separate upper and lower bounds with commas instead of hyphens as the latter can be confused with reporting of negative values.

- Please repeat statistical definitions (HR, CI etc.) for each set of parentheses."

* In the last sentence of the Abstract Methods and Findings section, please describe the main limitation(s) of the study's methodology.

* In the author summary, please revise formatting and ensure you use bullet points.

FUNDING STATEMENT

* The funding statement should include: specific grant numbers, initials of authors who received each award, URLs to sponsors’ websites. Also, please state whether any sponsors or funders (other than the named authors) played any role in study design, data collection and analysis, the decision to publish, or preparation of the manuscript. If they had no role in the research, include this sentence: “The funders had no role in study design, data collection and analysis, decision to publish, or preparation of the manuscript.

COMPETING INTERESTS STATEMENT

* Please revise Competing Interest statement and remove first sentence – PhD’s student research is not a conflict.

DATA AVAILABILITY

* PLOS defines the “minimal data set” to consist of the data set used to reach the conclusions drawn in the manuscript with related metadata and methods, and any additional data required to replicate the reported study findings in their entirety. Authors do not need to submit their entire data set, or the raw data collected during an investigation. Please submit the following data:

The values behind the means, standard deviations and other measures reported;

The values used to build graphs;

The points extracted from images for analysis.

* The Data Availability Statement (DAS) requires revision. Please include DAS in your manuscript. For each data source used in your study:

a) If the data are freely or publicly available, note this and state the location of the data: within the paper, in Supporting Information files, or in a public repository (include the DOI or accession number)

b) If the data are owned by a third party but freely available upon request, please note this and state the institutional owner of the data set and contact information for data requests (insitutional web or email address). Note that a study author cannot be the contact for the data.

c) If the data are not freely available, please describe briefly the ethical, legal, or contractual restriction that prevents you from sharing it. Please also include an appropriate institutional contact (insitutional web or email address) for inquiries (again, this cannot be a study author).

* For studies in which a novel model is central to the manuscript's findings, as is the case here, authors are responsible for providing the source code needed to replicate the study's findings in a repository (such as GitHub, SourceForge or Bitbucket) or a cloud computing service (such as Code Ocean). Please include a citable digital object identifier (DOI) or other persistent identifier of the code (such as through linkage with Zenodo). Protection of authors’ intellectual property will not be cause for exception. Please explain in the manuscript’s Data Availability Statement how readers can access the shared code.

* Please include the statement on code availability in the data availability statement. Please see guidance here:https://journals.plos.org/plosmedicine/s/code-sharing-guidance#loc-data-availability-statement-examples

ETHICS AND CONSENT

* Please revise for use of patient-centered language. Please note that patient-centered language is constructed with the use of post-modified nouns (e.g. 'patients with psoriasis’ (or similar) instead of ‘psoriasis patients’) putting the person first in the sentence structure.

FIGURES

* Please show graph axes beginning at zero. If this is not possible, please show a break in the axis.

* When a p value is given, please specify the statistical test used to determine it in the legend.

* Please consider avoiding the use of red and green in order to make your figure more accessible

* Please provide the unadjusted comparisons as well as the adjusted comparisons in all relevant Tables

* Please specify the variables controlled for in all relevant Tables

* Please consider if moving some of the figures shared in the supplementary information into the main text would aid the reader. I am asking specifically for Sup Fig 1 (STROBE flow diagram) – I think moving it to the method section as Figure 1 will add clarity in the method section and provide quick overview of the work.

ACKNOWLEDGMENTS

* Please include an acknowledgment of study participants.

OBSERVATIONAL, COHORT, CROSS-SECTIONAL, AND CASE CONTROL STUDIES

* Thank you for provide RECORD checklist as Supplementary Table S1. We do ask to provide it separately as S1 Checklist file and attach as separate supplementary file.

* Your study is observational and therefore causality cannot be inferred. Please remove language that implies causality and refer to associations instead.

--------------------------------------------------------------------------

Comments from Reviewers:

Reviewer #3: I greatly appreciate the authors' very thoughtful response - and Dr. Rockwood's personal input here. The paper is much clearer and the additional analyses are very helpful. The point of "acute" vs "critical" illness unmasking frailty, often in those younger than expected is very nicely now made. This will be a very important contribution to the frailty literature.

Reviewer #4: Thank you for this very thoughtful response and further analyses, as well as a helpful outline of the history behind this work (and by extension our field).

The stratified results are very interesting, thank you for including them. While the text also mentions them, they do not appear in the supplement that was included in this revision (S4-S7 per line 362 on page 22 of the manuscript). It would also be helpful for readers to present the hazard ratios of the FI-Lab and UK Biobank FI in a regression of the two together to provide some comparison of the magnitude of association. I hope these points are not troublesome; if addressed I have no further suggestions for this manuscript.

Any attachments provided with reviews can be seen via the following link:

[LINK]

# Author comment: Author response to Decision Letter 4

**DOI:** 10.1371/journal.pmed.1005004.r007

23 Jul 2026

Attachment Submitted filename: Response to Reviewers - July 23.docx

# Editor report: Decision Letter 4

**DOI:** 10.1371/journal.pmed.1005004.r008

**Authors:** Katarzyna Starowicz

24 Jul 2026

Dear Dr Rockwood,

On behalf of my colleagues and the Academic Editor, Sanjay Basu, I am pleased to inform you that we have agreed to publish your manuscript "Evaluation of the FI-Lab, a laboratory-based, automated frailty index for acute care: a multicohort study  " (PMEDICINE-D-26-00743R4) in PLOS Medicine.

Before your manuscript can be formally accepted you will need to complete some formatting changes, which you will receive in a follow up email. Please be aware that it may take several days for you to receive this email; during this time no action is required by you. Once you have received these formatting requests, please note that your manuscript will not be scheduled for publication until you have made the required changes.

In the meantime, please log into Editorial Manager at http://www.editorialmanager.com/pmedicine/, click the "Update My Information" link at the top of the page, and update your user information to ensure an efficient production process.

PRESS

We frequently collaborate with press offices. If your institution or institutions have a press office, please notify them about your upcoming paper at this point, to enable them to help maximise its impact. If the press office is planning to promote your findings, we would be grateful if they could coordinate with medicinepress@plos.org. If you have not yet opted out of the early version process, we ask that you notify us immediately of any press plans so that we may do so on your behalf.

We also ask that you take this opportunity to read our Embargo Policy regarding the discussion, promotion and media coverage of work that is yet to be published by PLOS. As your manuscript is not yet published, it is bound by the conditions of our Embargo Policy. Please be aware that this policy is in place both to ensure that any press coverage of your article is fully substantiated and to provide a direct link between such coverage and the published work. For full details of our Embargo Policy, please visit http://www.plos.org/about/media-inquiries/embargo-policy/.

To enhance the reproducibility of your results, we recommend that you deposit your laboratory protocols in protocols.io, where a protocol can be assigned its own identifier (DOI) such that it can be cited independently in the future. Additionally, PLOS ONE offers an option to publish peer-reviewed clinical study protocols. Read more information on sharing protocols at https://plos.org/protocols?utm_medium=editorial-email&utm_source=authorletters&utm_campaign=protocols

Thank you again for submitting to PLOS Medicine. We look forward to publishing your paper.

Sincerely,

Katarzyna Starowicz, PhD

Associate Editor

PLOS Medicine
